# Biocompatible Organic Coatings Based on Bisphosphonic Acid RGD-Derivatives for PEO-Modified Titanium Implants

**DOI:** 10.3390/molecules25010229

**Published:** 2020-01-06

**Authors:** Lyudmila V. Parfenova, Elena S. Lukina, Zulfia R. Galimshina, Guzel U. Gil’fanova, Veta R. Mukaeva, Ruzil G. Farrakhov, Ksenia V. Danilko, Grigory S. Dyakonov, Evgeny V. Parfenov

**Affiliations:** 1 Institute of Petrochemistry and Catalysis of Russian Academy of Sciences, 141, Prospekt Oktyabrya, 450075 Ufa, Russia; elenlykina@mail.ru (E.S.L.); zgalimshina@mail.ru (Z.R.G.); gilfanova.guzel@gmail.com (G.U.G.); 2 Department of Theoretical Basis of Electrical Engineering, Ufa State Aviation Technical University, 12 Karl Marx Street, 450008 Ufa, Russia; veta_mr@mail.ru (V.R.M.); frg1982@mail.ru (R.G.F.); evparfenov@mail.ru (E.V.P.); 3 Bashkir State Medical University, 3 Lenin Street, 450000 Ufa, Russia; kse-danilko@yandex.ru; 4 Institute of Physics of Advanced Materials, Ufa State Aviation Technical University, 12 Karl Marx Street, 450008 Ufa, Russia; dgr84@mail.ru

**Keywords:** titanium implants, plasma electrolytic oxidation, RGD peptide, bisphosphonic acid, in vitro tests, fibroblasts, mesenchymal stem cells, human osteosarcoma cells

## Abstract

Currently, significant attention is attracted to the problem of the development of the specific architecture and composition of the surface layer in order to control the biocompatibility of implants made of titanium and its alloys. The titanium surface properties can be tuned both by creating an inorganic sublayer with the desired morphology and by organic top coating contributing to bioactivity. In this work, we developed a composite biologically active coatings based on hybrid molecules obtained by chemical cross-linking of amino acid bisphosphonates with a linear tripeptide RGD, in combination with inorganic porous sublayer created on titanium by plasma electrolytic oxidation (PEO). After the addition of organic molecules, the PEO coated surface gets nobler, but corrosion currents increase. In vitro studies on proliferation and viability of fibroblasts, mesenchymal stem cells and osteoblast-like cells showed the significant dependence of the molecule bioactivity on the structure of bisphosphonate anchor and the linker. Several RGD-modified bisphosphonates of β-alanine, γ-aminobutyric and ε-aminocaproic acids with BMPS or SMCC linkers can be recommended as promising candidates for further in vivo research.

## 1. Introduction

Traumatology and orthopedics generate high demand in implants for osseosynthesis, and, according to market forecasts, this demand will increase due to the spread of extreme activities and the aging of the population. Currently, various biomimetic approaches that ensure the biocompatibility of the implants are developed [1]. As a rule, they suggest changes in the architecture and composition of the surface layer so that the devices gain the properties of the bone tissue and cell membranes. In current medical practice, preference is given to implants made of titanium and its alloys, due to their bioinert properties and high corrosion resistance [2]. The modification of the titanium surface is achieved both through inorganic coatings that approximate the phase composition and morphology of the human bone and by introduction of organic molecules containing functional fragments actively interacting with the proteins of the extracellular matrix (ECM). These approaches should provide both biomechanical and biochemical compatibility of the implants, their corrosion resistance, and, therefore, should successfully initiate the metal device osseointegration into the bone tissue.

Currently, significant attention is attracted to the biomimetic coatings based on the method of plasma electrolytic oxidation (PEO) [3,4]. The inorganic parts of the PEO coatings contain stable titania (rutile and anatase) tightly attached to the surface [5]. PEO helps to incorporate the anions and cations of the electrolyte into the coating; this provides Ca-, P- containing bioactive crystalline phases within the coating: hydroxyapatite, tricalciumphosphate, tetracalciumphosphate, perovskite [6]. Nevertheless, medical implants, once introduced into a biological system, get covered by nonspecific proteins within a few seconds. The adsorbed layer initiates a foreign body reaction of the organism; this causes the formation of a vascular fibrous capsule isolating the implant from the target tissue [7]. Consequently, current state-of-the-art includes the development of biocompatible bifunctional organic top coatings, which, on one hand would mask the implant, on the other hand, would initiate the implant osseointegration. These modern materials include: non-fouling coatings based on polysaccharides; hydrophilic self-assembled monolayers or polyethylene glycol, also in combination with oligopeptides (OP); bioactive coatings based on the extracellular matrix proteins (e.g., fibronectin, collagen, laminin, and osteopontin), having both long and short sequences of the amino acids [8,9,10,11,12]. The combination of the PEO inorganic sublayer and the organic top coating appears to be a promising direction for the development of implants with enhanced osseointegration [13,14,15].

Among relatively short bioactive peptide sequences, special attention is paid to RGD (arginine-glycine-aspartic acid), which is a tripeptide fragment appearing in numerous extracellular matrix proteins, and to which the cells attach using specific receptors on their surface [16]. Its discovery led to the identification of a large family of the cell surface adhesion receptors, called integrins, which recognize RGD-specific sequences in certain proteins of the extracellular matrix. The literature survey shows significant research efforts put into the application of the RGD-containing oligopeptides for the titanium surface modification. Various methods of the deposition have been developed: silanization of the surface by aminosilanes (including APTES) followed by the binding with RGD-containing oligopeptide via various linkers [17,18,19,20,21,22,23,24,25,26,27,28,29,30]; oligopeptide- functionalized synthetic polymers [31,32,33,34] and natural biopolymers [35,36,37,38,39]; oligopeptides attachment to the titanium via CDI [40,41] or tresyl chloride [42] surface activation; immobilization of OP on Ti via electrodeposited poly(ethyleneglycol) (PEG) [43,44,45]; physicochemical adsorption of oligopeptides as well as SH-ended derivatives [23,46,47,48,49,50,51] and 3,4-dihydroxyphenylalanine (DOPA) functionalized OP [52] from solutions; physicochemical adsorption of OP with a phosphonate anchor [22,23,36,51,53,54,55,56,57,58]. The strategy of the phosphonate anchors appears to be very attractive when bonding the inorganic oxide sublayer with the organic top coating. As shown elsewhere [59,60,61], phosphonate groups have high affinity to metal ions and their oxides, and they are almost not prone to hydrolytic cleavage, unlike siloxanes [62,63]. The introduction of gem-bisphosphonic group into the molecules increases their affinity to the oxidized surfaces compared to monophosphonates, and this increases their aqueous solubility due to the changing of the molecule polarity [64,65].

Plasma electrolytic oxidation currently is a well-developed electrochemical technique used to produce ceramic coatings with improved adhesion over the surface of valve metals such as titanium, magnesium, aluminum and zirconium [66,67,68]. Due to the unique surface morphology of the porous coating, PEO provides better biocompatibility of the surface and for the cell proliferation [69]. The PEO process is an expansion of the traditional anodizing into high voltages of up to 600 V; these voltages promote microdischarges within the coating; this results in its resoldifying and intensive growth [70]. The process mechanism includes electrochemical oxidation and numerous melting and crystallizing events at the microdischarge sites. This coating formation mechanism helps to develop coatings with regulated porosity, with the pore size from 0.1 to 10 µm and roughness Ra from 1 to 10 µm; the wide network of the pores forms a fractal-like structure, with the pore size increasing to the top of the coating [71]. The high surface area of the PEO coating allows deposition of various functional organic components, and the PEO coating acts as a sublayer increasing adhesion, and as the organic substance carrier [72,73,74,75,76].

Our recent study [15] showed that the combination of the PEO coating and RGD-modified bisphosphonic acid on nano-Ti gives a 45% increase in the number of proliferated cells compared to uncoated nano-Ti, and 66% compared to coarse grain Ti.

In the presented study, we expanded the number of hybrid RGD-functionalyzed molecules in order to uderstand how the structure of the bisphosphonate anchors and linkers (BMPS, EMCS, SMCC) affects the biological activity of the composite coatings based on inorganic porous PEO sublayer on titanium; moreover, the biological effect of the synthesized organic molecules was studied in vitro on a larger set of cell lines (mesenchymal stem cells, fibroblasts and osteoblast-like cells). Therefore, the aim of this work is to synthesize the hybrid molecules by chemical cross-linking of amino acid bisphosphonates with a linear RGD tripeptide through linkers that differ in length and structure for effective use in combination with inorganic porous PEO sublayer on titanium, and to identify the most promising options for the design of the proposed biomimetic coatings.

## 2. Results and Discussion

**Scheme 1:**
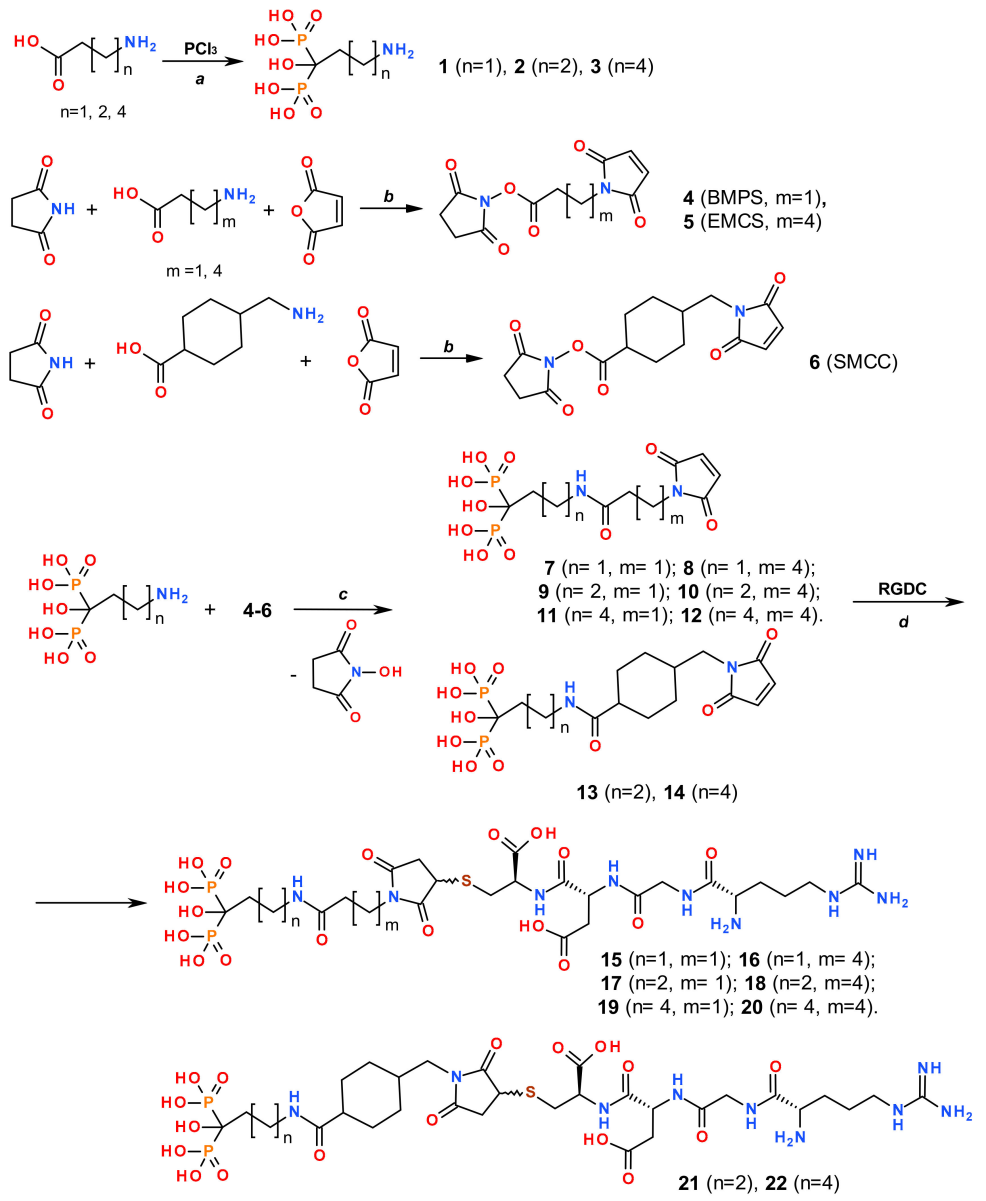
Reagents and conditions: (**a**) MeSO_3_H, 4–5 h, 85–90 °C; (**b**) DMF, 3–16 h, 0→25 °C; (**c**) H_2_O: acetone = 1:1, pH = 8–9, 1 h, 38–40 °C; (**d**) H_2_O, pH = 7, 1–1.5 h, 38–40 °C.

The experimental design concerns the variation of the anchors and linkers to attach the RGD peptide to the titanium implant. For the sake of the clarity, the following sample identification is used: Ti—for the metal substrate; Ti-PEO—for the inorganic PEO coating over the substrate; Ti-PEO-RGD—for the PEO coating soaked in the RGD aqueous solution; Ti-PEO-(number)-RGD—for the PEO coating soaked in the aqueous solution of the RGD-modified bisphosphonic acids, where the (number) indicates the compound in accordance with the reaction Scheme 1 and the corresponding description in Section 3.2. As a result, this sample identification shows the structures of the proposed composite coatings.

### 2.1. Synthesis of Bioactive Bifunctional Molecules

As a base for the design of the RGD-containing self- assembled monolayers on the PEO-modified meal surface, we synthesized aminoacid bisphosphonates **1–3** via the reaction of the corresponding amino acids (β-alanine, γ-aminobutyric and ε-aminocaproic acids) with PCl_3_ in methanesulfonic acid (Scheme 1). We found that carrying out the synthesis at elevated temperature (85–90 °C) decreases the reaction time to 4–5 h, and, along with the separation of the products at pH = 6, increases the resulting yield of the amino bisphosphonates up to 85–89%. The *N*-maleimidosuccinimide linkers (BMPS, EMCS and SMCC) **4**–**6** were obtained in yields of 60–89% via the reaction of maleic anhydride, *N*–hydroxysuccinimide and aminocarboxylic acids in DMF [77,78]. The *N*-maleimido derivatives **7**–**14** for further modification by the oligopeptide were obtained by interaction of amino bisphosphonates with *N*-maleimidosuccinimide linkers. The application of acetone instead of dioxane as the solvent used elsewhere [79] helped to avoid the formation of relatively heavy side products whose appearance is an inevitable consequence of the dioxane cycle modification. Target RGD-modified amino acid bisphosphonates **15**–**22** were obtained by the reaction of the corresponding derivatives **7**–**14** with RGDC in an aqueous medium at pH = 7 by Michael reaction.

The structure of the RGD-modified aminoacid bisphosphonates was confirmed by mass-spectrometry MALDI TOF/TOF, 1D (^1^H, ^13^C, ^31^P) and 2D NMR spectroscopy. In the MALDI TOF/TOF mass spectra of the hybrid molecules, the peaks with m/z corresponding to the molecular ions were detected. The ^31^P-NMR spectra exhibited single resonance lines at δ_P_ 17–18 ppm, characteristic to bisphosphonate groups. Signals corresponding to fragments of amino bisphosphonate, linker, and RGDC were observed in ^1^H- and ^13^C-NMR spectra. The formation of compounds **15**–**22** was accompanied by the disappearance of the signal of double bond protons of the *N*-maleimido fragment in derivatives **7**–**14** at δ_H_ 6–7 ppm in the ^1^H-NMR spectrum.

### 2.2. PEO Coating Characterization

**Figure 1:**
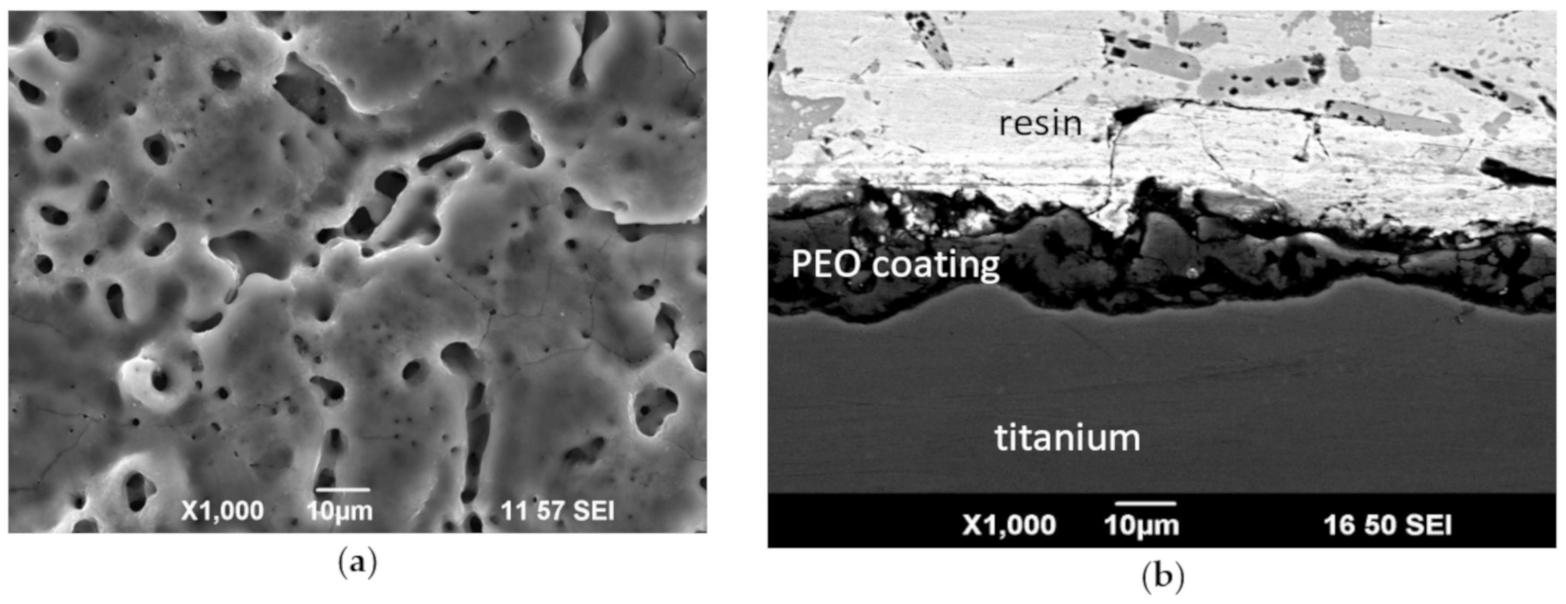
SEM images of the PEO coating: (**a**) Top view; (**b**) Cross-section.

Figure 1 shows the top view and cross-section of the PEO coating. The coating exhibits a porous morphology, with porosity evaluated as 18.2 ± 2.5%. The coating thickness is 19.3 ± 1.5 µm, surface roughness 1.5 ± 0.2 µm. This type of morphology contributes towards cell proliferation, and it supplies a well-developed surface for the introduction of the organic compounds with bisphosphonate anchors into the resulting composite coating [15]. The coating surface porosity is 9.5 ± 0.8%; the real 3D structure of this well-developed network of pores can be seen elsewhere for a similar PEO coating [80]. 

**Figure 2:**
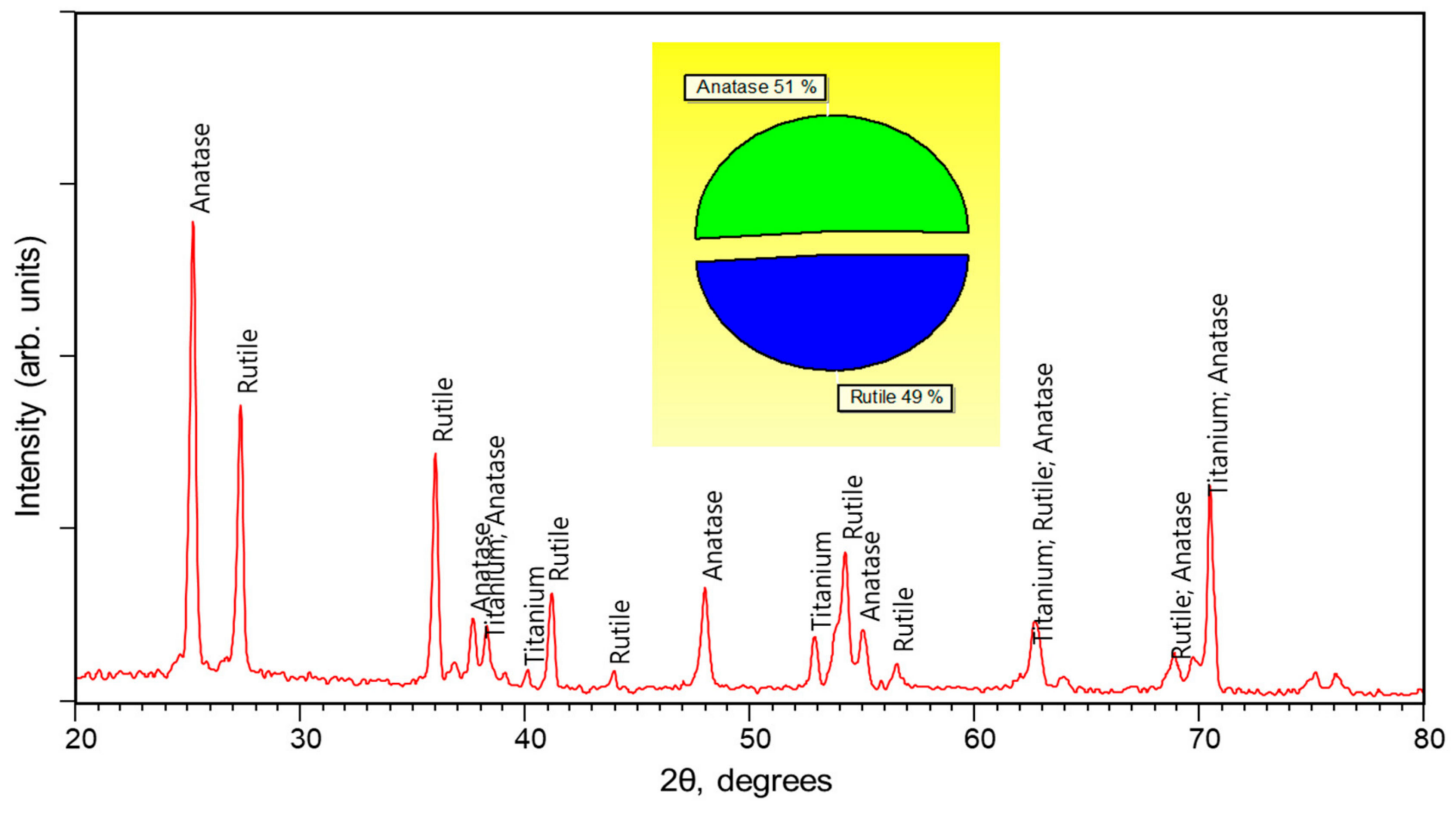
XRD pattern of the PEO coating with the labeled peaks and SemiQuant results.

As follows from Figure 2, the PEO coating consists of crystalline titania in two phase modifications—rutile and anatase (49 and 51% respectively). Also, the XRD pattern shows the peaks belonging to α-Ti substrate. This coating composition and morphology provides sufficient surface passivation for the implant prolonged operation in the corrosive fluids of a human body [81]. Also, this type of coating exhibits exceptional adhesion to the titanium surface as summarized elsewhere [5].

### 2.3. XPS Analysis of the Composite PEO Coating Functionalized with RGD Derivatives

**Figure 3:**
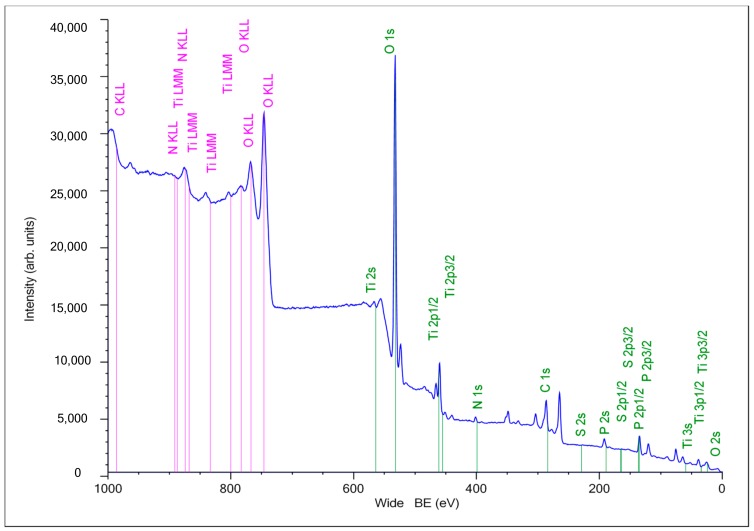
Survey XPS spectra of the Ti-PEO coating.

Since the introduction of the RGD derivatives into the inorganic PEO coating was performed at a low concentration of 10^−3^ M, it falls below the capabilities of the SEM and XRD techniques to detect organic compounds on the titania surface. Therefore, XPS studies were employed in order to verify the presence of the RGD derivatives within the coating; a typical survey spectrum is shown in Figure 3. In details the XPS spectra are presented in the Supplementary Materials. The survey XPS spectra collected from the PEO-coated titanium samples indicate typical Ti 2p, Ti 3s O 1s, C 1s, P 2p, N 1s, O KLL and Ti LMM Auger peaks. The spectra of the RGD-modified samples confirm changes in the surface layer chemistry compared to the unmodified PEO sample.

As follows from the high-resolution XPS spectra, the organic molecules attached to the surface affect the relative content of nitrogen, phosphorus, carbon, oxygen, and sulfur (Table 1). The most notable differences appear for the Ti2p, C1s and N1s peaks. The Ti2p signal decreased after the RGD modification, and the N1s and C1s signals increased. Also, the RGD-modified surface shows the presence of S2p signals, whereas for the Ti-PEO surface this signal does not appear. The changes in the atomic ratio (Ti2p/C1s) and (Ti2p/P2p) show that the titanium signal decreases for all the RGD modified types of the surface. This is consistent with the observations made elsewhere [27,82,83].

### 2.4. Electrochemical Behavior of the Composite PEO Coating Functionalized with RGD Derivatives

**Figure 4:**
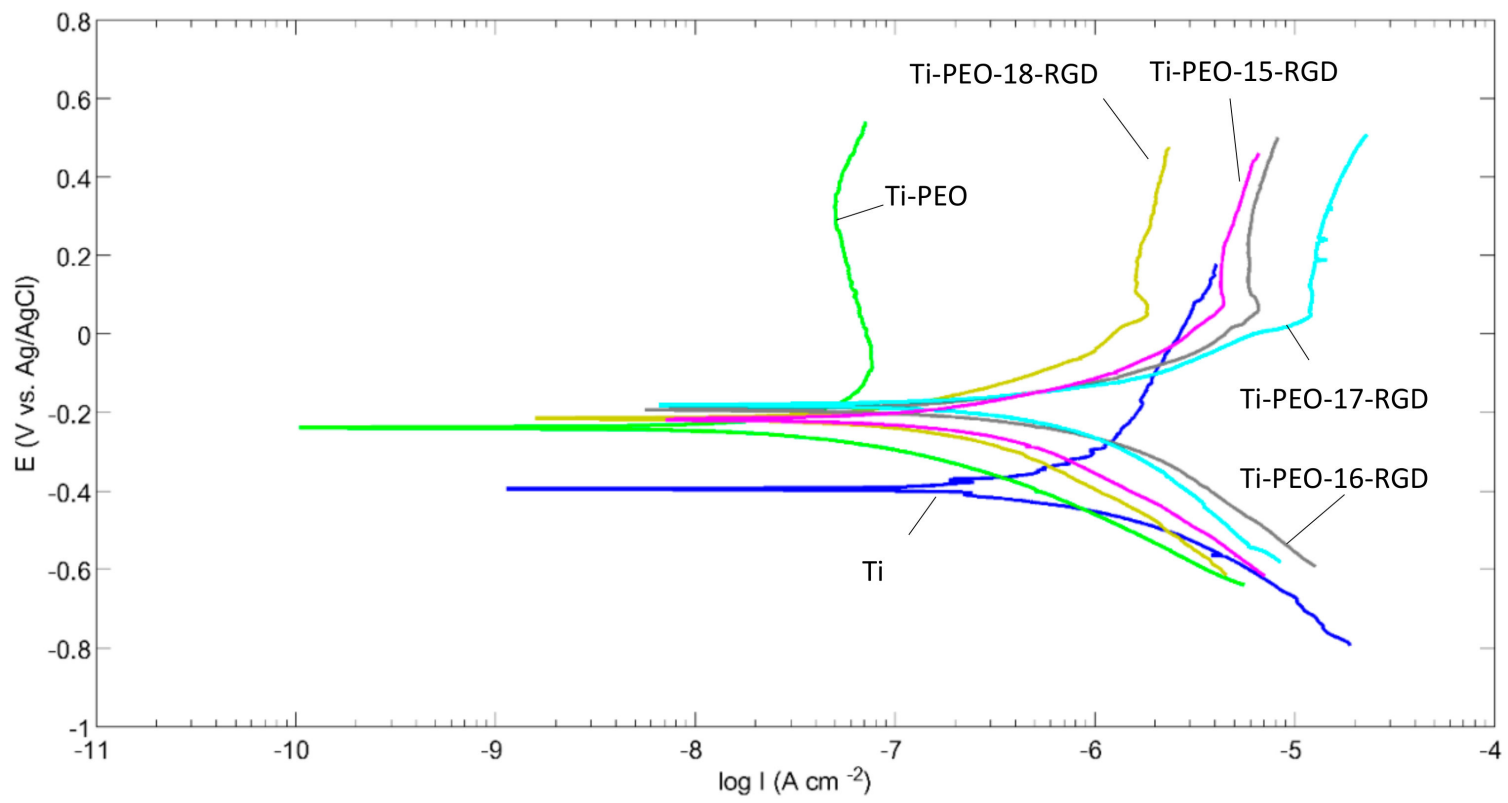
Polarization curves in Ringer’s solution for the Ti samples, with PEO coating, and RGD modification.

**Figure 5:**
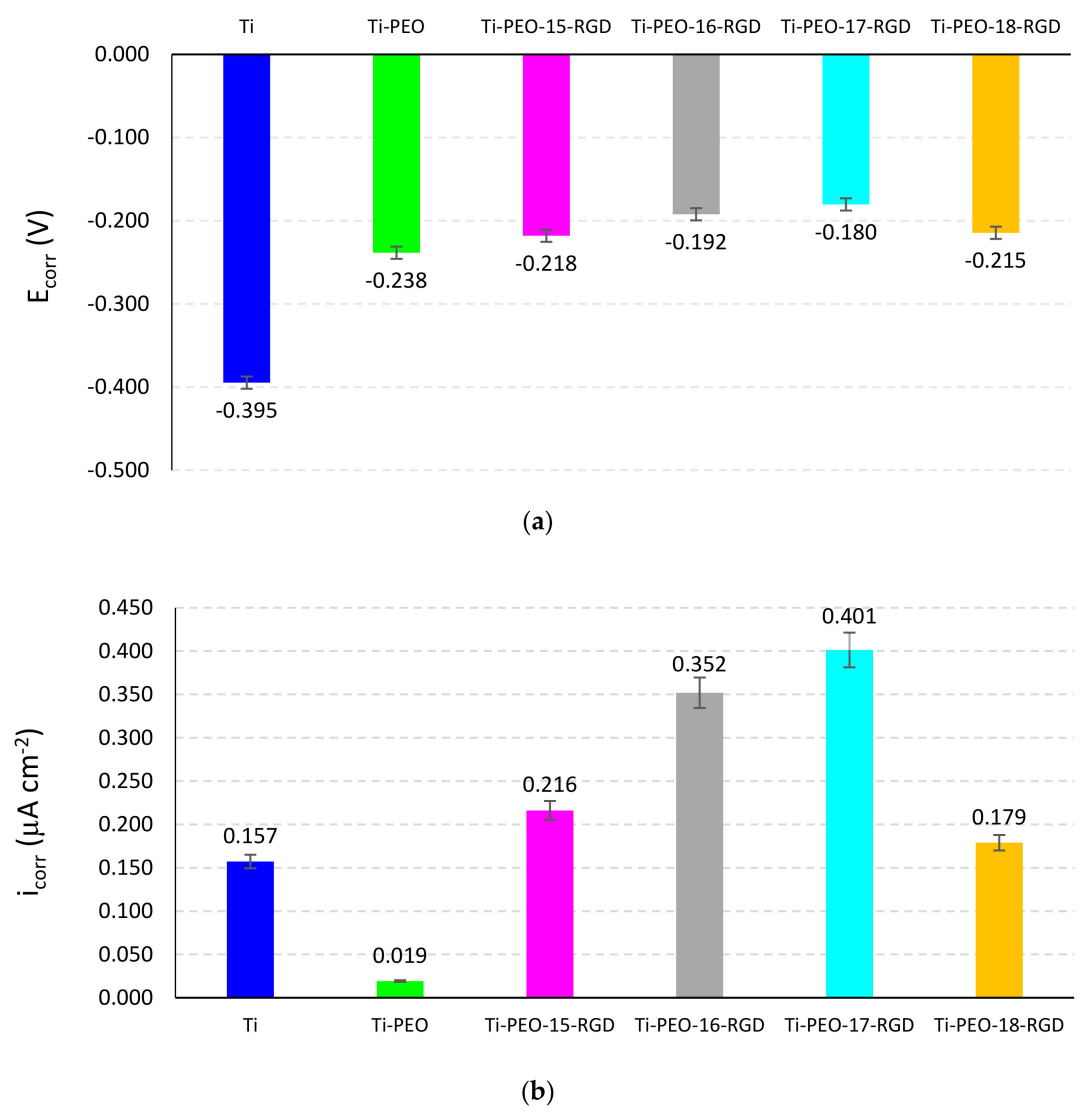

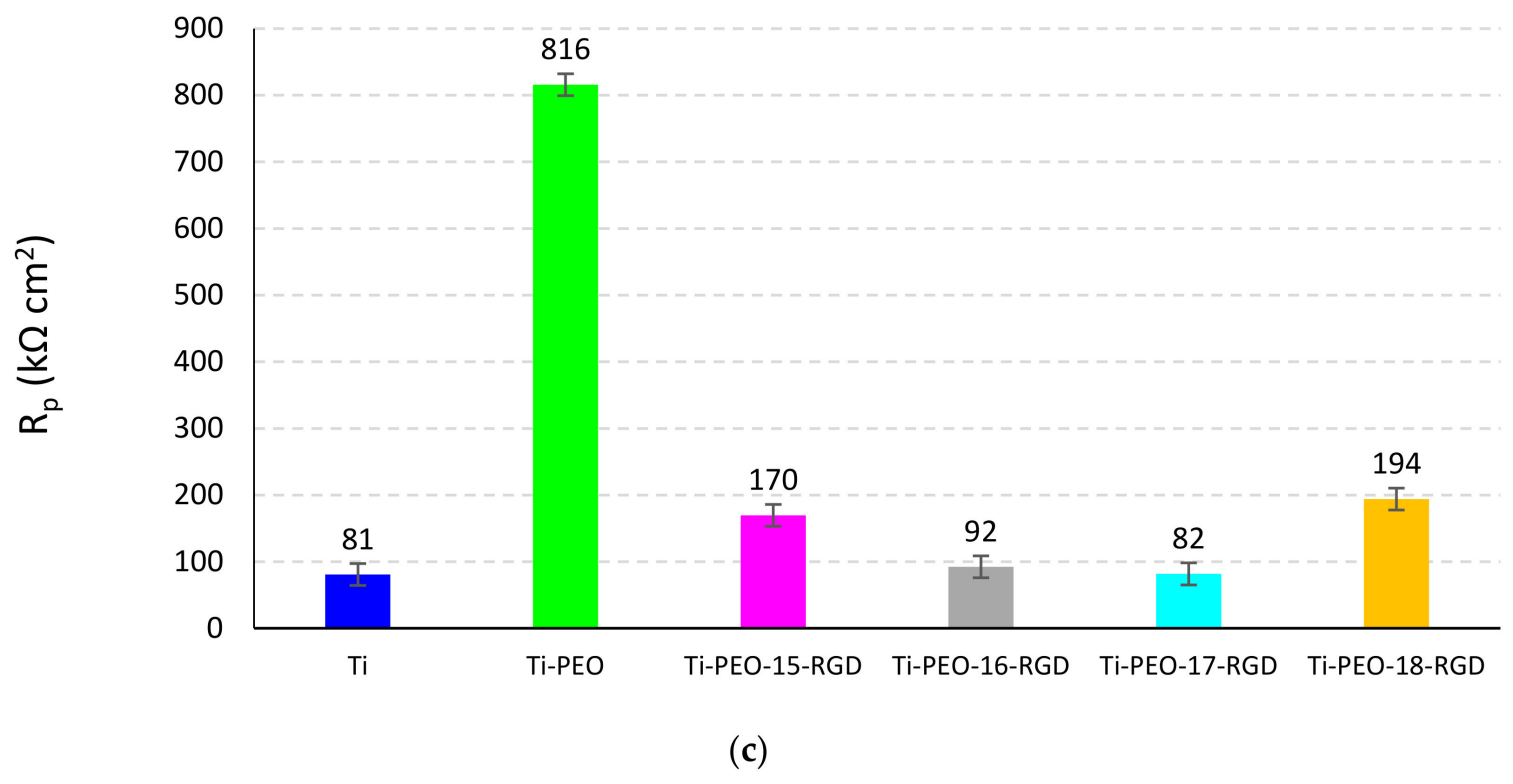
Electrochemical properties in Ringer’s solution for the Ti samples, with PEO coating, and RGD modification: (**a**) Corrosion potential E_corr_; (**b**) Corrosion current i_corr_; (**c**) Polarization resistance R_p_.

The electrochemical behavior of the composite PEO coating functionalized with RGD derivatives was studied using compounds **15**–**18** as examples. The polarization curves (Figure 4) show notable differences among the surface having PEO and various RGD treatments. As expected, after the PEO, the surface passivates, and the E_corr_ becomes nobler; the corrosion current i_corr_ decreases, which is followed by an order of magnitude increase in the polarization resistance R_p_ (Figure 5). All the RGD functionalized coatings show the surface, nobler than that of the Ti-PEO sample. The cathodic parts of the polarization curves do not show significant differences compared to Ti and Ti-PEO samples. This indicates that cathodic corrosion processes are not influenced by this type of coating since the cathodic Tafel parts show almost similar slopes. However, the anodic behavior is significantly influenced by the coating. Ti-PEO sample shows a passivation region above −0.1 V(AgCl). The introduction of the RGD- modified bisphosphonates depassivates the surface; the corrosion current increases compared to that of Ti-PEO and Ti samples (Figure 5). The depassivation region belongs to the range −0.1 to 0.1 V (AgCl) and ends with a pitting current tip followed by a passivation region above 0.1 V (AgCl). The changes in the anodic kinetics rise the E_corr_, and this is followed by the increase of the i_corr_. This indicates that the RGD functionalization increases the exchange currents, and this i_corr_ behavior is supported by the decrease of R_p_.

### 2.5. In Vitro Test Results Supporting The Efficiency of Proposed PEO Coating Functionalized with RGD Derivatives

As follows from the literature analysis, the application of RGD-containing bisphosphonates to the titanium surface leads to the increase in cell adhesion, proliferation and mineralization [22,23,51,53]. The cell adhesion appeared to be sensitive to the anchor used [57,59].

**Figure 6:**
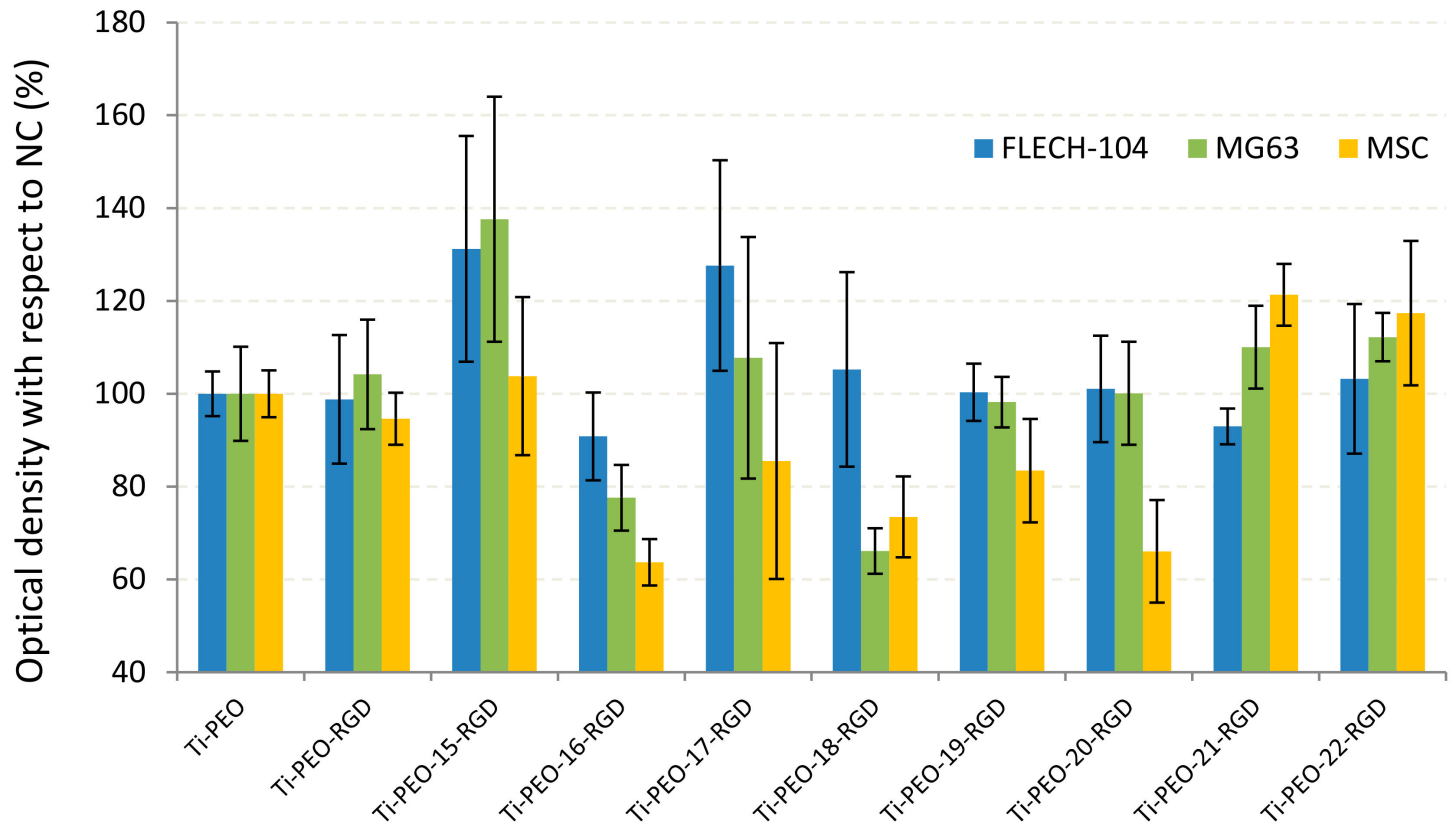
Optical density showing viability and proliferation of fibroblasts (FLECH-104), human osteoblast-like cells (MG-63) and mesenchymal stem cells (MSC) cultured on the surface of Ti-PEO functionalized by RGD-derivatives (**15**–**22**) after 7 days (metal samples were kept for 1 h at room temperature in the solutions of compounds **15**–**22** with concentrations 1.3 × 10^−3^–1.8 × 10^−3^ M/L and then dried).

The in vitro results presented in Figure 6 show that the different cell lines give dissimilar reactions to the same type of coating. The biological response also depends on the structure of the source amino bisphosphonate and the linker. It was shown that RGDC-oligopeptide itself introduced into the PEO coating without the anchor does not affect the cell life activity on the surface.

A significant (up to 30–40%) increase in the fibroblast proliferation was observed when using the organic molecules with relatively short linkers and aminobisphosphonate fragments BMPS-β (Ti-PEO-**15**-RGD) and BMPS-γ (Ti-PEO-**17**-RGD). Moreover, the sample Ti-PEO-**15**-RGD based on β-alanine bisphosphonate with the short BMPS linker and RGD peptide has shown the best adhesion for the osteoblast-like cells MG-63 compared to the other samples.

The derivatives containing EMCS linker (Ti-PEO-**16**-RGD and Ti-PEO-**18**-RGD) lead to a decrease in the proliferation of MG-63 on the PEO-modified surface. The same tendency was found in the case of mesenchymal stem cells then the long-chained molecules containing EMCS linker (Ti-PEO-**16**-RGD, Ti-PEO-**18**-RGD, and Ti-PEO-**20**-RGD) or derivative of ε-aminocaproic acid (Ti-PEO-**19**-RGD) were applied. In contrast, SMCC-linked compounds (Ti-PEO-**21**-RGD and Ti-PEO-**22**-RGD) provide the increasing of the cell growth up to 20% on the surface.

Summarizing the in vitro studies, we can conclude that RGD functionalized hybrid molecules **15**, **17**, **21**, **22** appear to be promising organic top coatings for modulation of the biological activity of the PEO modified surface of titanium implants. The differences in the cell behavior depending on the compound structure can be caused by their cytotoxicity and orientation on the PEO surface during adsorption; this requires further studies that are currently conducted in our research group.

## 3. Materials and Methods

### 3.1. General Information 

The following reagents were used for the synthesis: methanesulfonic acid (98%, Acros Organics, Geel, Belgium), PCl_3_ (98%, Acros Organics), β-alanine (97%, Acros Organics), *γ*-aminobutanoic (99+%, Acros Organics) and *ε*-aminocaproic acid (≥98.5%, Merk, Darmstadt, Germany), maleic anhydride (98+%, Acros Organics), *N*-hydroxysuccinimide (98+%, Acros Organics), dicyclohexylcarbodiimide (DCC, 99%, Acros Organics) and RGDC oligopeptide (Arg-Gly-Asp-Cys trifluoroacetate, Bachem, Bubendorf, Switzerland).

Spectroscopic studies were performed by ^1^H, ^13^C and ^31^P-NMR on an AVANCE-500 spectrometer (Bruker, Rheinstetten, Germany; operating frequency 500.17 MHz (^1^H), 125.78 MHz (^13^C) and 202.48 MHz (^31^P)). D_2_O and CDCl_3_ were used as internal standards and solvents. ^31^P-NMR chemical shifts are given relative to the standard, an 85% solution of H_3_PO_4_ in H_2_O (δ_P_ 0 ppm). Samples were prepared in a standard tube with a diameter of 5 mm. The chemical shifts of carbon and hydrogen atoms are given on the scale δ (ppm) with respect to TMS, the KCCB values (*J*) are given in Hz. One and two-dimensional NMR spectra (COSY HH, HSQC, HMBC, NOESY) were recorded using standard pulse sequences. Mass spectra were obtained on MALDI-TOF/TOF Autoflex III system (Bruker) using 2,5-dihydroxybenzoic acid (2,5-DHB) or α-cyano-4-hydroxycinnamic acid (CHCA) as matrices.

### 3.2. Synthesis of RGD-Functionalized Derivatives

#### 3.2.1. Synthesis of Amino-1-hydroxyalkan-1,1-diyl-bisphosphonic acids **1**–**3**

The synthesis of aminobisphosphonic acids was basd on a literature method [84] somewhat modified by us. A portion of the amino acid (0.025 mol) (β*–*alanine, γ–butanoic acid, ε*–*aminocaproic acid) is dissolved in 10 mL of methanesulfonic acid (MSA) in a flask with intensive stirring. Then, 7.0 mL (0.080 mol) of phosphorus (III) chloride is slowly added dropwise to the solution over 15 min. The contents of the flask are stirred under heating (80–85 °C) for 4–6 h, then cooled to room temperature. After cooling, 12 mL (0.67 mol) of water are added, and the reaction mixture is hydrolyzed at 105 °C for 4 h. After cooling, the pH of the solution is adjusted to 6–7 by adding to the mixture 12 mL of 50% aqueous sodium hydroxide solution. Then the reaction mass precipitated with 40 mL of MeOH. The precipitate is separated on the Schott filter. The crude product is dissolved in 20 mL of water, stirred at 50–70 °C for 30 min, then cooled and precipitated with 40 mL of MeOH. The solid phase is separated using a Schott filter.

*(3-Amino-1-hydroxypropane-1,1-diyl)-bisphosphonic acid* (**1**). Yield 5.3 g (85%). IR (ν, cm^−1^): 3448, 1613 (OH), 1526, 916 (NH_2_), 1377, 1171 (P=O), 2726, 2675, 1281 (CH_2_). ^1^H-NMR (D_2_O) δ: 2.18 (sept, ^3^*J* = 6.1 Hz, 2H, C^2^H_2_), 3.24 (t, ^3^*J* = 6.5 Hz, 2H, C^3^H_2_). ^13^C-NMR (D_2_O) δ: 30.52 (C^2^), 36.12 (C^3^), 72.64 (t, ^1^*J*_C-P_ = 133.8 Hz, C^1^). ^31^P-NMR (D_2_O) δ: 17.04. MALDI-TOF/TOF *m/z* 234.018 [M − H]^+^, calc. for C_3_H_10_NO_7_P_2_ 235.069.

(*4-Amino-1-hydroxybutane-1,1-diyl)-bisphosphonic acid* (**2**). Yield 5.5 g (88%). IR (ν, cm^−1^): 3460, 1651 (OH), 1538, 907 (NH_2_), 1376, 1149 (P=O), 2526, 2435, 1272 (CH_2_). ^1^H-NMR (D_2_O) δ: 1.77–1.94 (m, 4H, C^2^H_2_, C^3^H_2_), 2.82 (t, ^3^*J* = 6.1 Hz, 2H, C^4^H_2_). ^13^C-NMR (D_2_O) δ: 31.90 (C^2^), 23.93 (t, ^3^*J*_C-P_ = 5.5 Hz, C^3^), 40.56 (C^4^), 73.3 (t, ^1^*J*_C-P_ = 134.1 Hz, C^1^). ^31^P-NMR (D_2_O) δ: 17.93. MALDI TOF/TOF *m/z*: 247.086 [M − 2H]^+^, 269.069 [M − 2H + Na]^+^, 285.036 [M − 2H + K]^+^, calc. for C_4_H_13_NO_7_P_2_ 249.016.

*(6-Amino-1-hydroxyhexane-1,1-diyl)-bisphosphonic acid* (**3**). Yield 6.2 g (89%). IR (ν, cm^−1^): 3433, 1636 (OH), 1547, 910 (NH_2_), 1376, 1167 (P=O), 2723, 2671, 1282 (CH_2_). ^1^H-NMR (D_2_O) δ: 1.76–1.89 (m, 2H, C^2^H_2_), 1.63 (quint., ^3^*J* = 7.3 Hz, 2H, C^5^H_2_), 1.33 (quint., ^3^*J* = 7.3 Hz, 2H, C^4^H_2_), 1.47–1.58 (m, 2H, C^3^H_2_), 2.94 (t, ^3^*J* = 7.3 Hz, 2H, C^6^H_2_). ^13^C-NMR (D_2_O) δ: 22.87 (t, ^3^*J*_C-P_ = 5.6 Hz, C^3^), 26.17 (C^4^), 26.33 (C^5^), 33.35 (C^2^), 39.27 (C^6^), 74.07 (t, ^1^*J*_C-P_ = 134.7 Hz, C^1^). ^31^P-NMR (D_2_O) δ: 18.48. MALDI TOF/TOF, *m/z*: 299.193 [M—H + Na]^+^, calc. for C_6_H_17_NO_7_P_2_ 277.048.

#### 3.2.2. Synthesis of (*N*-Maleimidoalkyl)succinimide Esters (**4**–**6**)

The synthesis of compounds was carried out according to the method described in [77,78]. A portion of 3 mmol of an amino acid is added to a solution of maleic anhydride (0.3 g, 3 mmol) in 4 mL of DMF, stirred for 2 h at room temperature. After complete dissolution, at a temperature of 0 °C, 0.43 g (3.75 mmol) of *N*-hydroxysuccinimide (NHS) and 1.24 g (6 mmol) of dicyclohexylcarbodiimide (DCC) are added to the solution. The reaction mass is stirred in an ice bath 5–10 min and then incubated at room temperature for a day. The formation of a white precipitate of dicyclohexylurea is observed. The precipitate is washed with 20 mL of water and 20 mL of methylene chloride. The organic layer is treated with 10 mL of water, extracted with methylene chloride, then 10 mL of 5% NaHCO_3_ are added and extracted with methylene chloride (the procedure is carried out three times). The organic layer was dried over Na_2_SO_4_, the solution was filtered and the solvent was evaporated at the reduced pressure. The residue was purified by column chromatography on SiO_2_ with CHCl_3_/MeOH (from 1:0 to 100:1) as an eluent to give compounds **4**–**6**.

*(2,5-Dioxopyrrolidin-1-yl)-3-(2,5-dioxopyrrol-1-yl)propanoate (BMPS)* (**4**). Yield 0.47 g (60%). ^1^H-NMR spectrum matches the one reported in [85]. ^1^H-NMR (CDCl_3_) δ: 2.84 (s, 4H, NHS), 3.04 (t, *J* = 6.5 Hz, 2H, CH_2_C=O), 3.85 (t, *J* = 6.5 Hz, 2H, CH_2_N), 6.79 (s, 2H, CH=CH).

*(2,5-Dioxopyrrolidin-1-yl)-6-(2,5-dioxopyrrol-1-yl)hexanoate (EMCS)* (**5**). Yield 0.55 g (60%). The ^1^H-NMR spectrum corresponds to the one reported in [86] ^1^H-NMR (CDCl_3_) δ: 1.29 (quint, *J* = 7.6 Hz, 2H, CH_2_), 1.54 (quint, *J* = 7.6 Hz, 2H, CH_2_), 1.67 (quint, *J* = 7.2 Hz, 2H, CH_2_), 2.63 (t, *J* = 7.2 Hz, 2H, CH_2_C=O), 2.86 (s, 4H, NHS), 3.45 (d, *J* = 6.8 Hz, 2H, CH_2_N), 6.75 (s, 2H, CH=CH).

*(2,5-Dioxopyrrolidin-1-yl)-4-[(2,5-dioxopyrrol-1-yl)methyl]cyclohexanecarboxylate (SMCC)* (**6**). Yield 0.89 g (89%). ^1^H-NMR spectrum corresponds to the literature data [85,87]. ^1^H-NMR (CDCl_3_) δ: 1.01–1.14 (m, 2H, cyclohexyl CH_2_^ax^), 1.47–1.61 (m, 2H, cyclohexyl COCHCH_2_^ax^), 1.60–1.75 (m, 1H, cyclohexyl CH), 1.76–1.85 (m, 2H, cyclohexyl CH_2_^eq^), 2.11–2.22 (m, 2H, cyclohexyl COCHCH_2_^eq^), 2.55–2.64 (m, 1H, cyclohexyl COCH), 2.83 (s, 4H, NHS), 3.40 (d, *J* = 7.3 Hz, 2H, CH_2_N), 6.72 (s, 2H, CH=CH).

#### 3.2.3. Synthesis of Conjugates of Aminobisphosphonic Acids with Maleimidosuccinimide Linkers **7**–**14**

The synthesis described in [79] was modified. Bisphosphonates **1**–**3** (0.04 mmol) are dissolved in 0.6 mL of water, the pH of the solution is adjusted to 8–9 with 0.1 N NaOH solution (~150 μL). With good stirring, an equimolar amount of linker **4**–**6** (0.04 mmol) dissolved in 0.6 mL of acetone is added to the resulting solution. The reaction mass is stirred at room temperature for 15–30 min, then neutralized to pH = 7 with a 0.1 N HCl solution and concentrated under reduced pressure. Compounds **7**–**14** are obtained as white powders.

*(3-{[3-(2,5-Dioxo-2,5-dihydro-1H-pyrrol-1-yl)propanoyl]amino}-1-hydroxypropane-1,1-diyl)-bis-(phosphonic acid) (BMPS-*β*)* (**7**). Yield 0.009 g (60%). ^1^H-NMR (D_2_O): 1.93–2.09 (m, 2H, C^2^H_2_), 2.42 (t, ^3^*J* = 6.5 Hz, 2H, C^5^H_2_), 3.35 (t, ^3^*J* = 7.8 Hz, 2H, C^3^H_2_), 3.71 (t, ^3^*J* = 6.0 Hz, 2H, C^6^H_2_), 6.78 (s, HC=CH). ^13^C-NMR (D_2_O): 32.61 (C^2^), 34.37 (C^6^), 34.77 (C^5^), 35.57 (t, ^3^*J_C-P_* = 7.8 Hz, C^6^), 72.87 (t, ^1^*J_C-P_* = 133.7 Hz, C^1^), 134.43 (C^8^), 172.65 (C^4^*)*, 173.01 (C^7^). ^31^P-NMR (D_2_O): 17.68. MALDI-TOF/TOF *m/z* 446.093 [M + Na + K]^+^, 468.063 [M + 2Na + K]^+^, calc. for C_10_H_16_N_2_O_10_P_2_ 386.189.

*(3-{[6-(2,5-Dioxo-2,5-dihydro-1H-pyrrol-1-yl)hexanoyl]amino}-1-hydroxypropane-1,1-diyl)-bis-(phosphonic acid) (EMCS-*β*)* (**8**). *(4-{[3-(2,5-Dioxo-2,5-dihydro-1H-pyrrol-1-yl)propanoyl]amino}-1-hydroxybutane-1,1-diyl)bis(phosphonic acid) (BMPS-γ)* (**9**). Yield 0.014 g (88%). ^1^H-NMR (D_2_O): 1.63–1.76 (m, 2H, C^3^H_2_), 1.77–1.89 (m, 2H, C^2^H_2_), 2.42 (t, ^3^*J* = 6.4 Hz, 2H, C^6^H_2_), 3.06 (t, ^3^*J* = 6.8 Hz, 2H, C^4^H_2_), 3.71 (t, ^3^*J* = 6.6 Hz, 2H, C^7^H_2_), 6.78 (s, HC=CH). ^13^C-NMR (D_2_O): 23.18 (t, ^3^*J_C-P_* = 6.8 Hz, C^3^), 31.03 (C^2^), 34.46 (C^6^), 34.70 (C^7^), 40.14 (C^4^), 73.78 (t, ^1^*J_C-P_* = 134.8 Hz, C^1^), 134.47 (C^9^), 172.66 (C^8^*)*, 173.37 (C^5^). ^31^P-NMR (D_2_O): 18.20. MALDI-TOF/TOF *m/z* 400.080 [M]^+^, calc. for C_11_H_18_N_2_O_10_P_2_ 400.215.

*(4-{[6-(2,5-Dioxo-2,5-dihydro-1H-pyrrol-1-yl)hexanoyl]amino}-1-hydroxybutane-1,1-diyl)bis(phosphonic acid) (EMCS-γ)* (**10**). Yield 0.014 g (79%). ^1^H-NMR (D_2_O): 1.12–1.28 (m, 2H, C^8^H_2_), 1.41–1.58 (m, 4H, C^7^H_2_, C^9^H_2_), 1.66–1.80 (m, 2H, C^3^H_2_), 1.80–1.95 (m, 2H, C^2^H_2_), 2.14 (t, ^3^*J* = 7.5 Hz, 2H, C^6^H_2_), 3.11 (t, ^3^*J* = 6.8 Hz, 2H, C^4^H_2_), 3.42 (t, ^3^*J* = 6.9 Hz, 2H, C^10^H_2_), 6.76 (s, 2H, HC=CH). ^13^C-NMR (D_2_O): 23.36 (C^3^), 24.87 (C^7^), 25.26 (C^8^), 27.43 (C^9^), 31.06 (C^2^), 35.61 (C^6^), 37.34 (C^10^), 39.98 (C^4^), 73.81 (t, ^1^*J_C-P_* = 134.1 Hz, C^1^), 134.26 (C^12^), 173.41 (C^11^), 176.80 (C^5^). ^31^P-NMR (D_2_O): 18.21. MALDI-TOF/TOF *m/z* 440.322 [M − 2H]^+^, calc. for C_14_H_24_N_2_O_10_P_2_ 442.295.

*(6-{[3-(2,5-Dioxo-2,5-dihydro-1H-pyrrol-1-yl)propanoyl]amino}-1-hydroxyhexane-1,1-diyl)bis(phosphonic acid) (BMPS-ε)* (**11**). Yield 0.013g (79%). ^1^H-NMR (D_2_O): 1.11–1.22 (m, 2H, C^4^H_2_), 1.32–1.43 (m, 2H, C^5^H_2_), 1.42–1.56 (m, 2H, C^3^H_2_), 1.76–1.91 (m, 2H, C^2^H_2_), 2.41 (t, ^3^*J* = 6.0 Hz, 2H, C^8^H_2_), 3.04 (t, ^3^*J* = 6.8 Hz, 2H, C^6^H_2_), 3.71 (t, ^3^*J* = 6.4 Hz, 2H, C^9^H_2_), 6.82 (s, 2H, C^11^H_2_), ^13^C-NMR (D_2_O): 23.23 (t, ^3^*J_C-P_* = 5.8 Hz, C^3^), 27.00 (C^4^), 27.97 (C^5^), 33.56 (C^2^), 34.55 (C^9^), 34.83 (C^8^), 39.41 (C^6^), 74.18 (s, ^1^*J_C-P_* = 134.5 Hz, C^1^), 134.53 (C^11^), 172.60 (C^10^), 173.21 (C^7^). ^31^P-NMR (D_2_O): 18.50. MALDI-TOF/TOF *m/z* 428.086 [M]^+^, calc. for C_13_H_22_N_2_O_10_P_2_ 428.270.

*(6-{[6-(2,5-Dioxo-2,5-dihydro-1H-pyrrol-1-yl)hexanoyl]amino}-1-hydroxyhexane-1,1-diyl)bis(phosphonic acid) (EMCS-ε)* (**12**). Yield 0.014 g (75%). ^1^H-NMR (D_2_O): 1.13–1.21 (m, 2H, C^10^H_2_), 1.20–1.31 (m, 2H, C^4^H_2_), 1.38–1.52 (m, 2H, C^5^H_2_), 1.44–1.57 (m, 6H, C^3^H_2_, C^9^H_2_, C^11^H_2_), 1.78–1.92 (m, 2H, C^2^H_2_), 2.13 (t, ^3^*J* = 7.2 Hz, 2H, C^8^H_2_), 3.09 (t, ^3^*J* = 6.7 Hz, 2H, C^6^H_2_), 3.42 (t, ^3^*J* = 6.8 Hz, 2H, C^12^H_2_), 6.76 (s, 2H, C^14^H_2_). ^13^C-NMR (D_2_O): 23.23 (t, ^3^*J_C-P_* = 6.0 Hz, C^3^), 24.90 (C^9^), 25.33 (C^10^), 26.92 (C^4^), 27.26 (C^11^), 28.12 (C^5^), 33.55 (C^2^), 35.57 (C^8^), 37.46 (C^12^), 39.29 (C^6^), 74.21 (t, ^1^*J_C-P_* = 133.6 Hz, C^1^), 134.27 (C^14^), 173.41 (C^13^), 176.66 (C^7^). ^31^P- NMR (D_2_O): 18.52. MALDI-TOF/TOF *m/z*: 470.391 [M]^+^, calc. for C_16_H_28_N_2_O_10_P_2_ 470.348.

*{4-[({4-[(2,5-Dioxo-2,5-dihydro-1H-pyrrol-1-yl)methyl]cyclohexyl}carbonyl)amino]-1-hydroxybutane-1,1-diyl}bis(phosphonic acid) (SMCC-γ)* (**13**). Yield 0.017 g (93%). ^1^H-NMR (D_2_O): 0.86–1.00, 1.60–1.71 (m, 4H, C^8^H_2_), 1.16–1.34, 1.71–1.81 (m, 4H, C^7^H_2_), 1.48–1.65 (m, 1H, C^9^H), 1.64–1.78 (m, 2H, C^3^H_2_), 1.80–1.92 (m, 2H, C^2^H_2_), 2.06–2.16 (m, 1H, C^6^H), 3.10 (t, ^3^*J* = 6.7 Hz, 2H, C^4^H_2_), 3.28 (d, ^3^*J* = 7.0 Hz, 2H, C^10^H_2_), 6.75 (s, 2H, CH=CH). ^13^C-NMR (D_2_O): 23.46 (t, ^3^*J_C-P_* = 6.2 Hz, C^3^), 28.33 (C^7^), 29.14 (C^8^), 31.15 (C^2^), 36.05 (C^9^), 39.95 (C^4^), 43.47 (C^10^), 44.86 (C^6^), 73.83 (t, ^1^*J_C-P_* = 132.3, C^1^), 134.17 (C^12^), 173.58 (C^11^), 179.68 (C^5^). ^31^P-NMR (D_2_O): 18.25.

*{6-[({4-[(2,5-Dioxo-2,5-dihydro-1H-pyrrol-1-yl)methyl]cyclohexyl}carbonyl)amino]-1-hydroxyhexane-1,1-diyl}bis(phosphonic acid) (SMCC-ε)* (**14**). Yield 0.018 g (90%). ^1^H-NMR (D_2_O): 0.86–1.00, 1.61–1.70 (m, 4H, C^10^H_2_), 1.20–1.30 (m, 2H, C^4^H_2_), 1.23–1.34, 1.70–1.80 (m, 2H, C^9^H_2_), 1.38–1.52 (m, 2H, C^5^H_2_), 1.44–1.56 (m, 2H, C^3^H_2_), 1.48–1.64 (m, 1H, C^8^H), 1.77–1.90 (m, 2H, C^2^H_2_), 2.04–2.16 (m, 1H, C^8^H), 3.09 (t, ^3^*J* = 6.8 Hz, 2H, C^6^H_2_), 3.28 (d, ^3^*J* = 7.3 Hz, 2H, C^12^H_2_), 6.75 (s, 2H, CH=CH). ^13^C-NMR (D_2_O): 23.40 (t, ^3^*J_C-P_* = 5.8 Hz, C^3^), 26.90 (C^4^), 28.20 (C^9^), 28.34 (C^5^), 29.14 (C^10^), 33.73 (C^2^), 36.04 (C^11^), 39.18 (C^6^), 43.46 (C^12^), 44.78 (C^8^), 74.26 (t, ^1^*J_C-P_* = 131.3 Hz, C^1^), 134.18 (C^14^), 173.58 (C^13^), 179.56 (C^7^). ^31^P-NMR (D_2_O) 18.60. MALDI-TOF/TOF *m/z*: 499.066 [M + 3H]^+^, calc. for C_18_H_30_N_2_O_10_P_2_ 496.137.

#### 3.2.4. Synthesis of RGDC Derivatives **15**–**22**

According to [79], 5 mg (0.01 mmol) of RGDC was dissolved in 1.45 mL of bidistilled water, the pH was adjusted to 7 by addition of 0.1 N NaOH (~120 μL). An equivalent amount of compounds **7**–**14** (0.01 mmol) was added to the solution. The reaction mixture was stirred for 1–2 h at 38–40 °C until a pinkish-violet tint appeared, and then the solvent was removed under reduced pressure. Compounds **15**–**22** were obtained in quantitative yield as pink-white powders.

*RGD-BMPS-*β (**15**). ^1^H-NMR (D_2_O) δ: 1.55–1.68 (m, 2H, C^23^H_2_), 1.74–1.84 (m, 2H, C^22^H_2_), 1.96–2.10 (m, 2H, C^2^H_2_), 2.38–2.45 (m, 2H, C^5^H_2_), 2.52 (dd, ^3^*J* = 8.4 Hz, ^2^*J* = 16.0 Hz, 1H, C^16^*H*H), 2.66 (dd*,* ^3^*J* = 4.9 Hz, ^2^*J* = 16.0 Hz, 1H, C^16^H*H*), 2.94 (dd, ^3^*J* = 8.5 Hz, ^2^*J* = 14.0 Hz, 2H, C^11^H_2_), 3.11–3.20 (m, 3H, C^24^H_2_, C^11^*H*H), 3.31–3.43 (m, 2H, C^3^H_2_), 3.70 (t, ^3^*J* = 7.0 Hz, 2H, C^6^H_2_), 3.70–3.80 (m, 1H, C^21^H), 3.87 (d, 1H, ^2^*J* = 16.9 Hz, C^19^*H*H), 4.02 (d, ^2^*J* = 16.9 Hz, 1H, C^19^H*H*), 4.40 (dd, ^3^*J* = 4.1 Hz, ^3^*J* = 8.5 Hz, 1H, C^12^H), 4.58–4.70 (m, 1H, C^15^H). ^13^C-NMR (D_2_O): 23.77 (C^23^), 29.49 (C^22^), 32.97 (C^2^), 34.79 (C^5^), 35.84 (C^6^), 36.07 (C^3^), 38.21 (C^16^), 39.95 (C^11^), 40.59 (C^24^), 42.48 (^19^), 51.39 (C^15^), 53.20 (C^21^), 54.39 (C^12^), 156.76 (C^25^), 170.81 (C^18^), 172.54 (C^14^), 172.64 (C^4^), 173.82 (C^20^), 176.43 (C^13^), 178.03 (C^17^), 178.91 (C^7^, C^10^). ^31^P-NMR (D_2_O): 17.93. MALDI-TOF/TOF *m/z*: 836.169 [M + H]^+^, calc. for C_25_H_43_N_9_O_17_P_2_S 835.671.

*RGD-EMCS-*β (**16**). NMR and mass spectrometry data correspond to those reported in [15].

*RGD-BMPS-γ* (**17**). ^1^H-NMR (D_2_O) δ: 1.58–1.70 (m, 2H, C^24^H_2_), 1.66–1.77 (m, 2H, C^3^H_2_), 1.80–1.91 (m, 2H, C^2^H_2_), 1.85–1.94 (m, 2H, C^23^H_2_), 2.40–2.46 (m, 2H, C^6^H_2_), 2.62–2.84 (m, 2H, C^17^H_2_), 2.98 (dd, ^3^*J* = 8.3 Hz, ^2^*J* = 14.0 Hz, 1H, C^12^*H*H), 3.01–3.12 (m, 2H, C^4^H_2_), 3.10–3.20 (m, 1H, C^12^H*H* ), 3.14–3.20 (m, 2H, C^25^H_2_), 3.68–3.75 (m, 2H, C^7^H_2_), 3.85–3.97 (m, 1H, C^20^*H*H), 3.98–4.08 (m, 2H, C^20^H*H*), 3.99–4.09 (m, 1H, C^22^H), 4.39–4.46 (m, 1H, C^13^H), 4.66–4.73 (m, 1H, C^16^H). ^13^C-NMR (D_2_O): 23.22 (C^3^), 23.46 (C^24^), 27.84 (C^23^), 31.01 (C^2^), 33.62 (C^6^), 35.82 (C^7^), 36.60 (C^17^), 40.14 (C^12^), 40.32 (C^4^, C^25^), 42.31 (C^20^), 50.53 (C^16^), 52.82 (C^22^), 54.21 (C^13^), 156.76 (C^26^), 169.98 (C^19^), 172.01 (C^15^, C^21^), 172.89 (C^5^), 175.97 (C^14^), 176.14 (C^18^), 178.31 (C^8^, C^11^). ^31^P-NMR (D_2_O): 18.17. MALDI-TOF/TOF *m/z*: 849.15 [M]^+^, calc. for C_26_H_45_N_9_O_17_P_2_S 849.698.

*RGD-EMCS-γ* (**18**). ^1^H NMR (D_2_O) δ: 1.16–1.31 (m, 2H, C^8^H_2_), 1.38–1.55 (m, 4H, C^7^H_2_, C^9^H_2_), 1.53–1.67 (m, 2H, C^27^H_2_), 1.61–1.77 (m, 2H, C^26^H_2_), 1.66–1.77 (m, 2H, C^3^H_2_), 1.77–1.90 (m, 2H, C^2^H_2_), 2.05–2.20 (m, 2H, C^6^H_2_), 2.52 (dd, ^3^*J* = 8.4 Hz, ^2^*J* = 16.2 Hz, 1H, C^20^*H*H), 2.65 (dd, ^3^*J* = 4.8 Hz, ^2^*J* = 16.2 Hz, 1H, C^20^H*H*), 2.93 (dd, ^3^*J* = 8.5 Hz, ^2^*J* = 14.0 Hz, 1H, C^15^*H*H), 3.16 (dd, ^3^*J* = 4.0 Hz, ^2^*J* = 14.0 Hz, 1H, C^15^H*H*), 3.08–3.15 (m, 2H, C^4^H_2_), 3.10–3.18 (m, 2H, C^28^H_2_), 3.42 (t, ^3^*J* = 7.0 Hz, 2H, C^10^H_2_), 3.54 (t, ^3^*J* = 6.1 Hz, 1H, C^25^H), 3.86 (d, ^3^*J* = 16.8 Hz, 1H, C^23^*H*H), 4.01 (d, ^3^*J* = 16.8 Hz, 1H, C^23^H*H*), 4.40 (dd, ^3^*J* = 4.0 Hz, ^3^*J* = 8.5 Hz, 1H, C^16^H), 4.59–4.68 (m, 1H, C^19^H). ^13^C-NMR (D_2_O): 23.73 (C^3^), 23.98 (C^27^), 25.45 (C^8^), 25.67 (C^7^), 27.73 (C^9^), 30.53 (C^26^), 31.48 (C^2^), 35.70 (C^6^), 38.20 (C^20^), 38.72 (C^10^), 39.75 (C^4^), 39.82 (C^15^), 40.71 (C^28^), 42.45 (C^23^), 51.37 (C^19^), 53.79 (C^25^), 54.32 (C^16^), 156.75 (C^29^), 170.95 (C^22^), 172.61 (C^18^), 175.85 (C^17^), 176.44 (C^24^), 176.96 (C^5^), 178.01 (C^21^). ^31^P-NMR (D_2_O): 18.24. MALDI-TOF/TOF *m/z*: 910.533 [M − 4H + Na]^+^, 926.498 [M − 4H + K]^+^, calc. for C_29_H_51_N_9_O_17_P_2_S 891.778.

*RGD-BMPS-ε* (**19**). ^1^H-NMR (D_2_O) δ: 1.18–1.32 (m, 2H, C^4^H_2_), 1.38–1.46 (m, 2H, C^5^H_2_),1.46–1.58 (m, 2H, C^3^H_2_), 1.57–1.72 (m, 2H, C^26^H_2_), 1.80–1.93 (m, 2H, C^2^H_2_), 1.84–1.96 (m, 2H, C^25^H_2_), 2.42 (t, ^3^*J* = 6.7 Hz, 2H, C^8^H_2_), 2.72–2.89 (m, 2H, C^19^H_2_), 2.94–3.02 (m, 1H, C^14^*H*H), 3.00–3.10 (m, 2H, C^6^H_2_), 3.10–3.20 (m, 1H, C^14^H*H*), 3.14–3.20 (m, 2H, C^27^H_2_), 3.67–3.75 (m, 2H, C^9^H_2_), 3.87–4.09 (m, 2H, C^22^H_2_), 3.98–4.08 (m, 1H, C^24^H), 4.40–4.51 (m, 1H, C^15^H), 4.65–4.77 (m, 1H, C^18^H). ^13^C-NMR (D_2_O): 23.19 (C^3^), 23.42 (C^26^), 26.99 (C^4^), 27.89 (C^5^, C^25^), 33.58 (C^2^), 33.71 (C^8^), 35.75 (C^9^), 35.93 (C^19^), 39.50 (C^6^, C^14^), 40.35 (C^27^), 42.29 (C^22^), 50.32 (C^18^), 52.81 (C^24^), 53.88 (C^15^), 156.76 (C^28^), 170.08 (C^23^), 170.74 (C^21^), 171.93 (C^17^), 172.75 (C^7^), 174.32 (C^16^), 174.80 (C^20^), 177.71 (C^10^, C^13^). ^31^P-NMR (D_2_O): 18.65. MALDI-TOF/TOF *m/z:* 879.242 [M + 2H]^+^, calc. for C_28_H_49_N_9_O_17_P_2_S 877.751.

*RGD-EMCS-ε* (**20**). ^1^H-NMR (D_2_O) δ: 1.15–1.30 (m, 4H, C^4^H_2_, C^10^H_2_), 1.41–1.53 (m, 2H, C^5^H_2_), 1.42–1.50 (m, 2H, C^3^H_2_), 1.45–1.61 (m, 4H, C^9^H_2_, C^11^H_2_), 1.54–1.65 (m, 2H, C^29^H_2_), 1.64–1.74 (m, 2H, C^28^H_2_), 1.74–1.90 (m, 2H, C^2^H_2_), 2.13 (t, ^3^*J* = 7.4 Hz, 2H, C^8^H_2_), 2.48–2.72 (m, 2H, C^22^H_2_), 2.88–2.99 (m, 1H, C^17^*H*H), 3.12–3.29 (m, 1H, C^17^H*H*), 3.06–3.17 (m, 2H, C^6^H_2_), 3.10–3.18 (m, 2H, C^30^H_2_), 3.42 (t, ^3^*J* = 7.10 Hz, 2H, C^12^H_2_), 3.45–3.52 (m, 1H, C^27^H), 3.82–3.92(m, 1H, C^25^*H*H), 3.93–4.04 (m, 1H, C^25^H*H*), 4.40 (dd, ^3^*J* = 4.0 Hz, ^3^*J* = 8.6 Hz, 1H, C^18^H), 4.56–4.72 (m, 1H, C^21^H). ^13^C-NMR (D_2_O): 23.55 (t, ^3^*J_C-P_* = 6.0 Hz, C^3^), 24.03 (C^29^), 25.48 (C^9^), 26.15 (C^10^), 26.36 (C^4^, C^11^), 30.89 (C^28^), 33.97 (C^2^), 35.61 (C^8^), 38.23 (C^22^), 38.94 (C^12^), 39.40 (C^6^, C^17^), 40.72 (C^30^), 42.40 (C^25^), 51.43 (C^21^), 53.93 (C^27^), 54.64 (C^18^), 74.37 (C^1^), 156.74 (C^31^), 171.03 (C^24^), 172.61 (C^20^), 177.92 (C^23^), 176.46 (C^19^), 176.56 (C^7^, C^26^), 180.08 (C^13^, C^16^). ^31^P-NMR (D_2_O): 18.71. MALDI-TOF/TOF *m/z*: 920.398 [M + H]^+^, calc. for C_31_H_55_N_9_O_17_P_2_S 919.831.

*RGD-SMCC-γ* (**21**). ^1^H-NMR (D_2_O) δ: 0.83–0.99 (m, 2H, C^8^*H*H), 1.19–1.30 (m, 2H, C^7^*H*H), 1.37–1.50 (m, 1H, C^9^H), 1.52–1.65 (m, 2H, C^27^H_2_), 1.55–1.72 (m, 2H, C^26^H_2_), 1.67–1.77 (m, 2H, C^3^H_2_), 1.68–1.77 (m, 2H, C^8^H*H*), 1.77–1.85 (m, 2H, C^7^H*H*), 1.78–1.87 (m, 2H, C^2^H_2_), 1.96–2.06 (m, 1H, C^6^H), 2.53 (dd, ^3^*J* = 8.2 Hz, ^2^*J* = 16.0 Hz, 1H, C^22^*H*H), 2.66 (dd, ^3^*J* = 4.8 Hz, ^2^*J* = 16.0 Hz, 1H, C^22^H*H*), 2.99–3.05 (m, 2H, C^10^H_2_), 2.93 (dd, ^3^*J* = 8.6 Hz, ^2^*J* = 14.0 Hz, 1H, C^15^*H*H), 3.17 (dd, ^3^*J* = 4.1 Hz, ^2^*J* = 14.0 Hz, 1H, C^15^H*H*), 3.08–3.12 (m, 2H, C^4^H_2_), 3.11–3.16 (m, 2H, C^28^H_2_), 3.41 (t, ^3^*J* = 6.0 Hz, 1H, C^25^H), 3.85 (d, ^2^*J* = 16.8 Hz, 1H, C^23^*H*H), 4.00 (d, ^2^*J* = 16.8 Hz, 1H, C^23^H*H*), 4.40 (dd, ^3^*J* = 4.1 Hz, ^3^*J* = 8.6 Hz, 1H, C^16^H), 4.63 (dd, ^3^*J* = 4.8 Hz, ^3^*J* = 8.2 Hz, 1H, C^19^H). ^13^C-NMR (D_2_O): 23.86 (C^3^), 24.13 (C^27^), 29.25 (C^7^), 29.53 (C^8^), 29.56 (C^2^), 31.25 (C^26^), 36.71 (C^9^), 38.27 (C^20^), 39.71 (C^15^), 40.17 (C^4^), 40.83 (C^28^), 42.45 (C^23^), 45.41 (C^10^), 46.98 (C^6^), 51.36 (C^19^), 54.06 (C^25^), 54.32 (C^16^), 156.74 (C^29^), 171.11 (C^22^), 172.63 (C^18^), 176.49 (C^17^), 177.91 (C^21^), 178.01 (C^24^). MALDI-TOF/TOF *m/z*: 941.786 [M + H + Na]^+^, calc. for C_31_H_53_N_9_O_17_P_2_S 917.275.

*RGD-SMCC-ε* (**22**). ^1^H-NMR (D_2_O) δ: 0.84–1.00 (m, 2H, C^10^*H*H), 1.18–1.32 (m, 2H, C^9^*H*H), 1.20–1.30 (m, 2H, C^4^H_2_), 1.37–1.52 (m, 1H, C^11^H), 1.41–1.52 (m, 2H, C^5^H_2_), 1.48–1.56 (m, 2H, C^3^H_2_), 1.55–1.66 (m, 2H, C^29^H_2_), 1.66–1.80 (m, 2H, C^10^H*H*), 1.66–1.82 (m, 2H, C^28^H_2_), 1.79–1.85 (m, 2H, C^9^H*H*), 1.77–1.87 (m, 2H, C^2^H_2_), 1.96–2.06 (m, 1H, C^8^H), 2.53 (dd, ^3^*J* = 8.4 Hz, ^2^*J* = 16.0 Hz, 1H, C^22^*H*H), 2.67 (dd, ^3^*J* = 5.0 Hz, ^2^*J* = 16.0 Hz, 1H, C^22^H*H*), 2.99–3.05 (m, 2H, C^12^H_2_), 2.94 (dd, ^3^*J* = 8.5 Hz, ^2^*J* = 14.0 Hz, 1H, C^17^*H*H), 3.18 (dd, ^3^*J* = 4.1 Hz, ^2^*J* = 14.0 Hz, 1H, C^17^H*H*), 3.10 (t, ^3^*J* = 6.7 Hz, 2H, C^6^H_2_), 3.13–3.19 (m, 2H, C^30^H_2_), 3.58–3.66 (m, 1H, C^27^H), 3.87 (d, ^2^*J* = 16.9 Hz, 1H, C^25^*H*H), 4.02 (d, ^2^*J* = 16.9 Hz, 1H, C^25^H*H*), 4.41 (dd, ^3^*J* = 4.1 Hz, ^3^*J* = 8.5 Hz, 1H, C^18^H), 4.64 (dd, ^3^*J* = 50 Hz, ^3^*J* = 8.4 Hz, 1H, C^21^H). ^13^C-NMR (D_2_O): 23.61 (C^3^), 23.92 (C^29^), 27.01 (C^4^), 28.29 (C^5^), 29.26 (C^9^), 29.54 (C^10^), 30.19 (C^28^), 34.22 (C^2^), 36.77 (C^11^), 38.25 (C^22^), 39.26 (C^6^), 39.86 (C^17^), 40.69 (C^30^), 42.48 (C^25^), 45.45 (C^12^), 46.92 (C^8^), 51.38 (C^21^), 53.67 (C^27^), 54.36 (C^18^), 156.75 (C^31^), 172.59 (C^20^), 175.44 (C^26^), 176.46 (C^19^), 178.03 (C^23^), 179.85 (C^7^). ^31^P-NMR (D_2_O): 18.77.

### 3.3. Metal Sample Preparation and PEO Coating

The metal samples 10 mm in diameter were cut using a wire-EDM out of a commercially pure Cp-Ti sheet, 0.5 mm thick. Further, the disk samples were polished on a SiC grit to achieve the surface roughness Ra < 0.1 µm. Before the plasma electrolytic oxidation, the samples were washed with deionized water and ultrasonically cleaned in isopropanol for 5 min. To achieve a good contact both the for the PEO and consequent electrochemical tests, a copper wire was attached into a small hole drilled at the sample edge. The contact place and the copper wire were coated with an epoxy resin which could withstand the electrochemical treatments. In order to produce the samples for the in vitro tests, a titanium screw holder was used to supply current to the sample. The holder was PEO coated to form a dielectric layer over its surface except the contact points prior to the treatment of the samples.

The plasma electrolytic oxidation was processed in a 10-L glass vessel equipped with a stainless steel heat exchanger arranged around its walls inside the electrolyte which comprised 20 g/L aqueous solution of Na_3_PO_4_·12H_2_O of puris grade. The electrolyte temperature was maintained at 20 ± 1 °C using microcontroller regulation. The PEO process was run in a pulsed bipolar mode under the voltage regulation; the positive pulse was 470 V, negative 40 V; the frequency was 300 Hz. The positive pulse duration was 1.7 ms, the negative pulse was 0.87 ms; both separated with pauses of 0.38 ms. The PEO treatment duration was 5 min.

### 3.4. Surface Characterization

The PEO coating was studied using a JSM-6490LV scanning electron microscope (JEOL, Tokyo, Japan) at accelerating voltage of 10 kV. Both top view and cross-sections were examined. The coating thickness was measured using a Positector 6000 eddy current gauge (Defelsko, Ogdensburg, NY, USA) of N-type. The surface roughness was measured with TR-220 profilometer. In order to evaluate the coating porosity, the SEM images were processed using ImageJ software using the technique described elsewhere [88]. By adjusting the threshold in the grayscale images, the relative surface area taken by the objects of interest being darker than the neighboring places was calculated by the program in percents. The phase composition of the surface layer was characterised by an Ultima IV X-ray diffractometer (Rigaku, Tokyo, Japan) in Cu Kα radiation at 40 kV and 40 mA using 0.02 deg. step scan with 2 s exposure, from 20 to 80 degrees 2θ. Further, the XRD spectra were processed using the X’Pert Highscore Plus software (Philips) with PDF2 pattern database and a built-in SemiQuant algorithm was employed to quantify the amounts of the crystalline phases in the coating.

XPS spectra were obtained using a JEOL JPS 9010MX spectrometer equipped with an (Mg K_α_) X-ray source. The pressure in the analytical chamber during the spectral acquisition was less than 7 × 10^−8^ Pa. The survey spectra were collected from 0 to 1000 eV with a pass energy of 50 eV, and high-resolution spectra were collected for each detected element of interest (C, N, O, P, S, and Ti) with a pass energy of 10 eV. The JEOL SpecSurf Program V. 1.9.2 was used to identify the characteristic peaks, to calculate the elemental compositions, and to fit the peaks of the high-resolution spectra.

### 3.5. Electrochemical Tests

The electrochemical tests were carried out in Ringer’s solution (0.86 wt% NaCl, 0.03 wt% KCl, 0.033 wt% CaCl2, pH 7.4) at temperature 37.0 ± 0.2 °C using a P-5X electrochemical system (Elins, Moscow, Russia). After the open circuit potential (OCP) was settled within ±20 mV for at least 30 min, the potentiodynamic polarization (PDP) test was run from −350 mV to +350 mV with respect to the settled OCP at a rate of 0.25 mV/s. The reference electrode was a silver chloride electrode filled with 3.5M KCl. The counter electrode was a platinum rod. The PDP results were processed using Tafel analysis [89]. For the PDP curves having linear Tafel regions at ±200 mV overpotentials, both anodic and cathodic slopes were used; for those having only cathodic linear Tafel regions, only cathodic slopes were used. The corrosion current i_corr_ was evaluated at the intersection of cathodic and anodic (if available) tangent lines with the level of E_corr_. The polarization resistance Rp was evaluated as a slope of the polarization curve at±10 mV overpotentials for all the samples [90].

### 3.6. In Vitro Assessment

The PEO coated Ti samples were ultrasonically cleaned for 10 min in 95% ethanol and finally washed with deionized water, dried on air and sterilized by autoclaving at 134 °C. This temperature does not affect the PEO coating. In order to deposit the organic pore filler, the PEO coated Ti samples were put into a Petri dish with 10^−3^ M solutions of the RGD-derivatives, which were preliminarily sterilized by filtration with CA 0.22 µm filter. In 1 h the samples were dried on air in a laminar box. Then, all the samples were put into a plastic 24-well tissue culture plate.

Human embryonic lung fibroblasts (FLECH-104) and mesenchymal stem cells of human fat tissue (MSC) were purchased from BIOLOT (Saint Petersburg, Russia), human osteosarcoma cells (MG-63) were obtained from Russian cellular collection, Institute of Cytology RAS (Saint Petersburg, Russia). The cells were cultured in Dulbecco’s modified Eagle’s medium (DMEM) (Sigma) containing 10% fetal bovine serum (FBS) (BioWest) and gentamicin, in 25-cm^2^ culture flasks (SPL life Sciences, Pocheon-si, South Korea) in a humidified 5% CO_2_ atmosphere. The medium was changed twice a week. After reaching a monolayer, the cells were detached using 0.25% trypsine solution (PanEco, Moscow, Russia) and counted using automated cell counter TC20 (BioRad, Hercules, CA, USA).

The FLECH-104, MSC or MG-63 cells suspension was placed into each well of the plate with the samples (0.8 mL containing 20·10^3^ cells). The cells in the wells with uncoated Ti+PEO samples were treated as a control. The culture plates were incubated for 7 days in the standard conditions (37 °C, 5 vol% CO_2_). The culture plate itself (polystyrene) was used as a blank.

The cell proliferation was determined by EZ4U assay (Biomedica, Vienna, Austria), a modification of MTT test, which evaluates the cell metabolic activity is proportional to the number of adherent cells. Three samples of each type were transferred after the incubation into another 24-well plate with 0.8 mL of fresh DMEM medium. Then 80 µL of activated EZ4U solution was added to every well and incubated at 37 °C, 5 vol% CO_2_ for 3.5 h. Optical absorbance was measured using microplate reader (Spark10M, Tecan, Männedorf, Switzerland) at 450 nm with a reference wavelength of 620 nm. The optical density per mm^2^ was calculated as:

(1) $$\mathrm{OD}\left(\%\right)=\frac{\left(a-a_{blank}\right)}{\left(A-a_{blank}\right)}\cdot 100\%$$

where a—absorbance of the test sample at 450 nm minus absorbance at 620 nm; A—absorbance of the control sample at 450 nm minus absorbance at 620 nm; $a_{blank}$—the absorbance of the blank solution of DMEM with no cells at 450 nm and at 620 nm.

The mean value and the standard deviation for four measurements of the optical density were calculated with respect to the control.

## 4. Conclusions

We have synthesized a set of RGD-derivatives of amino bisphosphonates, obtained from β-alanine, γ-aminobutyric and ε-aminocaproic acids, containing various linkers (BMPS, EMSC, SMCC) and used them as organic top coatings on porous PEO layer on titanium. The compounds can be introduced into the PEO coating pores due to the presence of bisphosphonate groups that facilitate physicochemical adsorption. Electrochemical studies showed that after RGD functionalization, the PEO surface gets slightly nobler, but the corrosion currents notably increase; also, the anodic PDP curves of the PEO surface lose the passivation region after the introduction of the RGD-derivatives, so their presence can be assessed by indirect electrochemical tests. The presence of organic molecules in the coating was confirmed by XPS spectroscopy. The appearance of compounds **15**–**20** on the surface provided a decrease in the Ti 2p line intensity and an increase of N1s and C1s line intensities; in addition, a significant decrease in the ratio of Ti 2p/C 1s and Ti 2p/P 2p was observed.

In vitro studies on proliferation and viability of fibroblasts, mesenchymal stem cells and osteoblast-like cells showed the dependence of the molecule bioactivity on the structure of the anchor and linker. In particular, RGD derivatives with relatively short bisphosphonate anchors and BMPS linker, as well as molecules containing a linker with a cyclohexyl fragment, increase cell proliferation on the surface of PEO-modified titanium. RGDC without anchor on Ti-PEO does not affect the cell proliferation. RGD-functionalyzed β-BMPS, γ-BMPS, γ-SMCC, ε-SMCC can be recommended as promising candidates for further in vivo research.

## Figures and Tables

**Table 1:** Atomic composition and atomic ratio derived from XPS high-resolution spectra for the Ti-PEO samples with and without RGD modification.

*Sample*	XPS Atomic Composition (at %)						Atomic Ratio	
	N1s	P2p	C1s	O1s	Ti2p	S2p	Ti2p/C1s	Ti2p/P2p
Ti-PEO	1.48	3.57	7.00	70.49	17.36	0.00	2.48	4.86
Ti-PEO-**15**-RGD	5.43	6.96	13.34	67.25	6.72	0.30	0.50	0.97
Ti-PEO-**16**-RGD	4.87	4.60	13.54	60.59	16.39	0.00	1.21	3.56
Ti-PEO-**17**-RGD	2.37	6.06	15.48	65.20	10.79	0.00	0.70	1.78
Ti-PEO-**18**-RGD	5.86	3.01	25.92	57.20	7.50	0.50	0.29	2.49
Ti-PEO-**19**-RGD	2.88	3.52	14.32	67.96	11.10	0.21	0.78	3.15
Ti-PEO-**20**-RGD	2.35	6.00	18.03	67.36	6.27	0.00	0.35	1.05

## Supplementary Materials

The following are available online. Figures S1–S27: NMR and mass-spectra of compounds **1**–**22**, Figure S28: XPS spectra of Ti-PEO modified by compounds **15**–**20**.
