# Supplementary material for: Biocompatible Organic Coatings Based on Bisphosphonic Acid RGD-Derivatives for PEO-Modified Titanium Implants
Source: Molecules. 2020 Jan 6;25(1):229. doi: 10.3390/molecules25010229 (PMC6982944; doi:10.3390/molecules25010229)

Biocompatible organic coatings based on bisphosphonic acid RGD-derivatives for PEO-modified titanium implants

Lyudmila V. Parfenova[1,*], Elena S. Lukina[1], Zulfia R. Galimshina[1], Guzel U. Gil'fanova[1], Veta R. Mukaeva[2], Ruzil G. Farrakhov[2], Ksenia V. Danilko[3], Grigory S. Dyakonov[4], Evgeny V. Parfenov[2]

[1]Institute of Petrochemistry and Catalysis of Russian Academy of Sciences, 141, Prospekt Oktyabrya, Ufa 450075, Russian Federation
[2]Department of Theoretical Basis of Electrical Engineering, Ufa State Aviation Technical University, 12 Karl Marx Street, Ufa 450008, Russian Federation
[3]Bashkir State Medical University, 3 Lenin Street, Ufa 450000, Russian Federation
[4]Institute of Physics of Advanced Materials, Ufa State Aviation Technical University, 12 Karl Marx Street, Ufa 450008, Russian Federation
Correspondence: luda_parfenova@ipc-ras.ru

Supporting Information

Figure S1. ^{1}H, ^{13}C and ^{31}P NMR of compound (**1**)........ 3
Figure S2. ^{1}H, ^{13}C and ^{31}P NMR of compound (**2**)........ 5
Figure S3. ^{1}H, ^{13}C and ^{31}P NMR of compound (**3**)........ 7
Figure S4. ^{1}H NMR of compound (**4**). 9
Figure S5. ^{1}H NMR of compound (**5**). 10
Figure S6. ^{1}H NMR of compound (**6**). 11
Figure S8. ^{1}H, ^{13}C and ^{31}P NMR of compound (**8**)........ 14
Figure S9. ^{1}H, ^{13}C and ^{31}P NMR of compound (**9**)........ 16
Figure S10. ^{1}H, ^{13}C and ^{31}P NMR of compound (**10**)........ 18
Figure S11. ^{1}H, ^{13}C and ^{31}P NMR of compound (**11**)........ 20
Figure S12. ^{1}H, ^{13}C and ^{31}P NMR of compound (**12**)........ 22
Figure S13. ^{1}H, ^{13}C and ^{31}P NMR of compound (**13**)........ 24
Figure S14. ^{1}H, ^{13}C and ^{31}P NMR of compound (**14**)........ 26
Figure S15. ^{1}H and ^{31}P NMR of compound (**15**)........ 28
Figure S16. MALDI TOF/TOF of compound (**15**). 29
Figure S17 ^{1}H and ^{31}P NMR of compound (**17**)........ 30
Figure S18. MALDI TOF/TOF of compound (**17**). 31
Figure S19. ^{1}H and ^{31}P NMR of compound (**18**)........ 32
Figure S20. MALDI TOF/TOF of compound (**18**). 33

Figure S21. ^{1}H and ^{31}P NMR of compound (**19**). 34
Figure S22. MALDI TOF/TOF of compound (**19**). 35
Figure S23. ^{1}H and ^{31}P NMR of compound (**20**). 36
Figure S24. MALDI TOF/TOF of compound (**20**). 37
Figure S25. ^{1}H and ^{31}P NMR of compound (**21**). 38
Figure S26. MALDI TOF/TOF of compound (**21**). 39
Figure S27. ^{1}H and ^{31}P NMR of compound (**22**). 40
Figure S28. XPS spectra of Ti-PEO modified by compounds **15**-**20**.......... 42

Figure S1. ^{1}H, ^{13}C and ^{31}P NMR of compound (**1**).

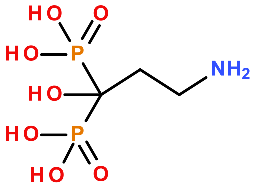

^{1}H NMR

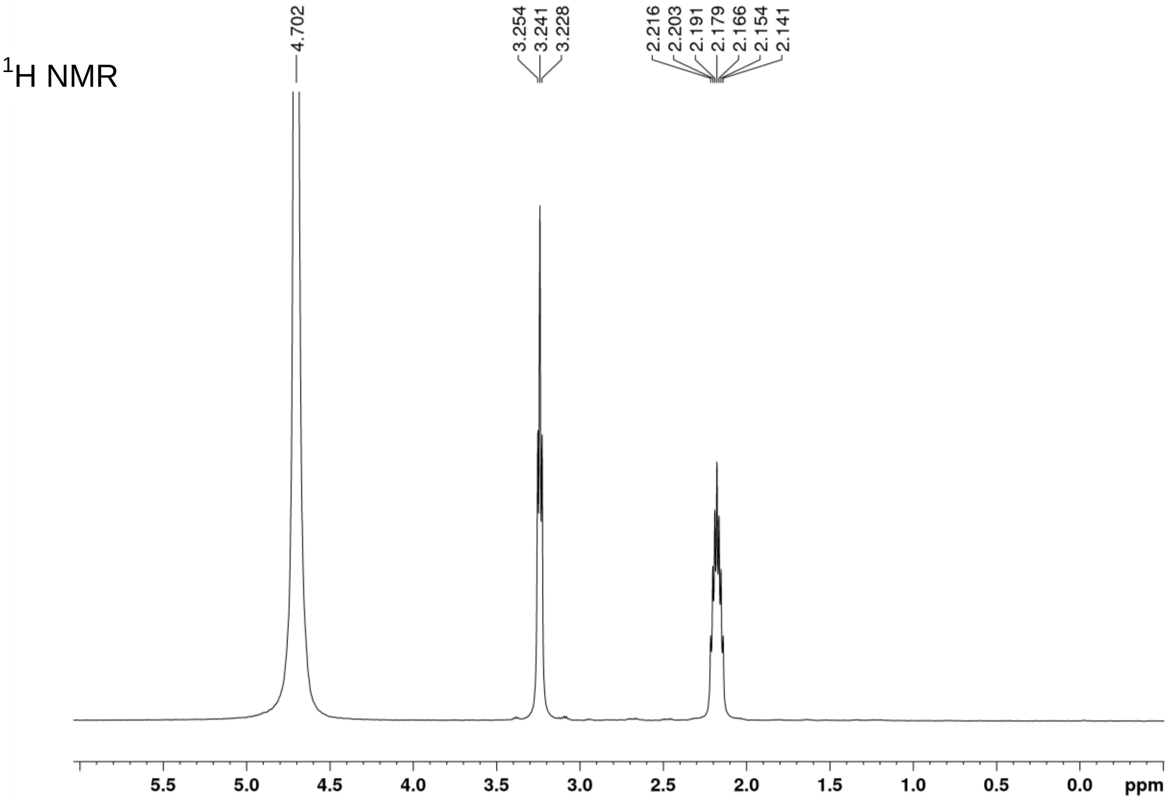

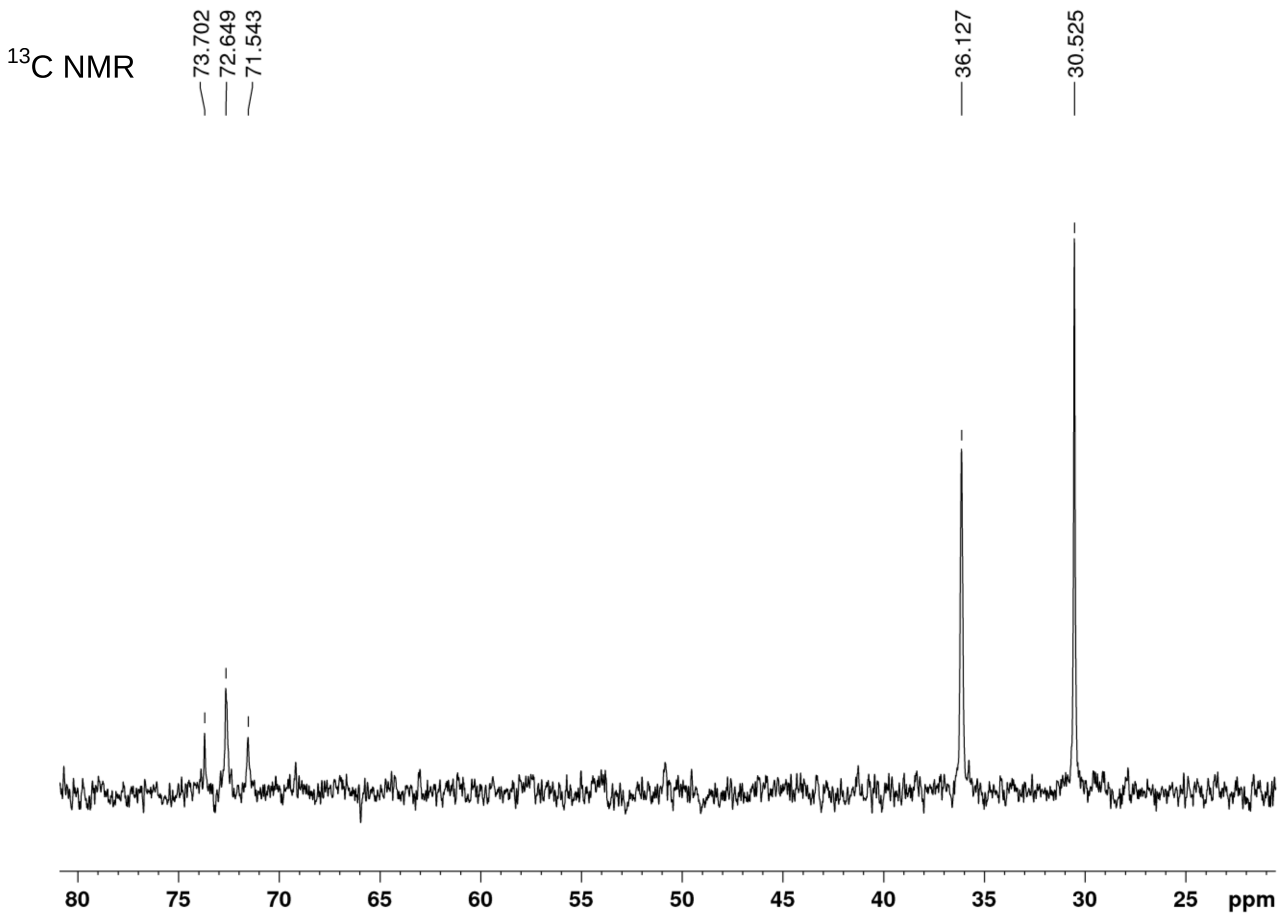

^{13}C NMR
73.702
72.649
71.543
36.127
30.525
80
75
70
65
60
55
50
45
40
35
30
25
ppm

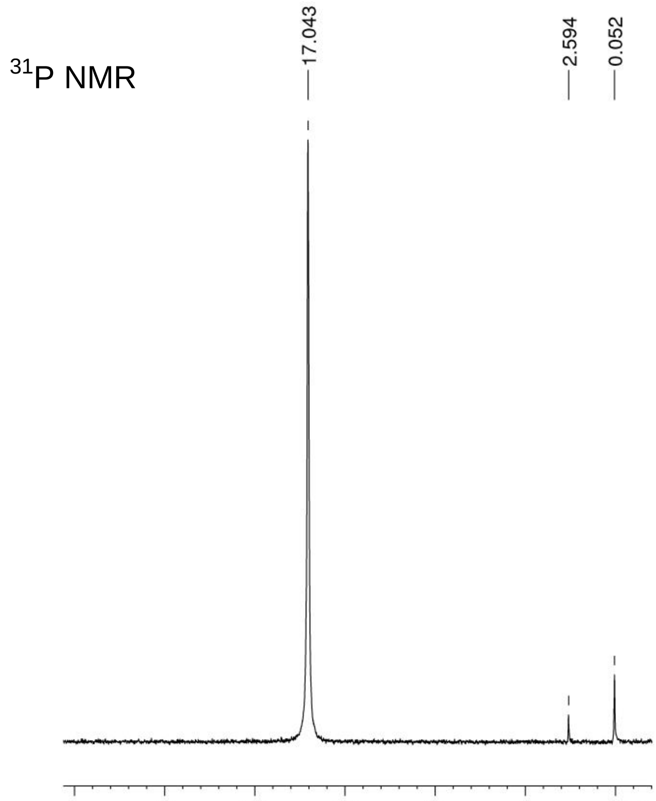

^{31}P NMR
17.043
2.594
0.052
25
20
15
10
5
ppm

Figure S2. ^{1}H, ^{13}C and ^{31}P NMR of compound (**2**).

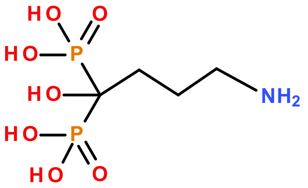

^{1}H NMR

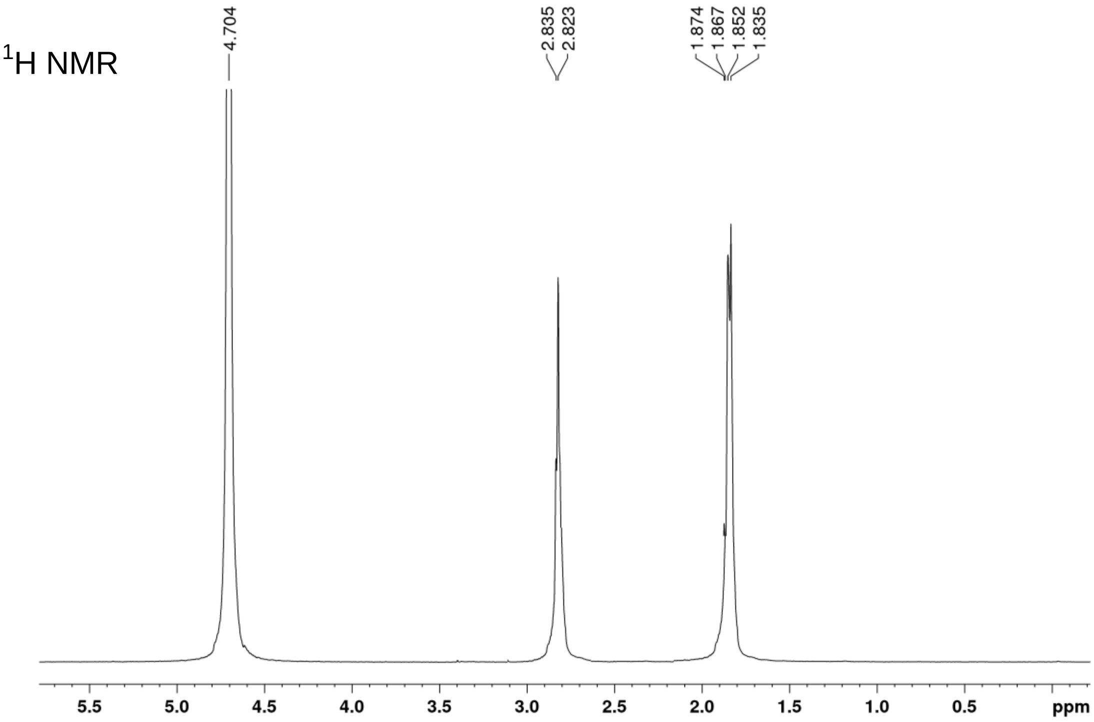

^{13}C NMR

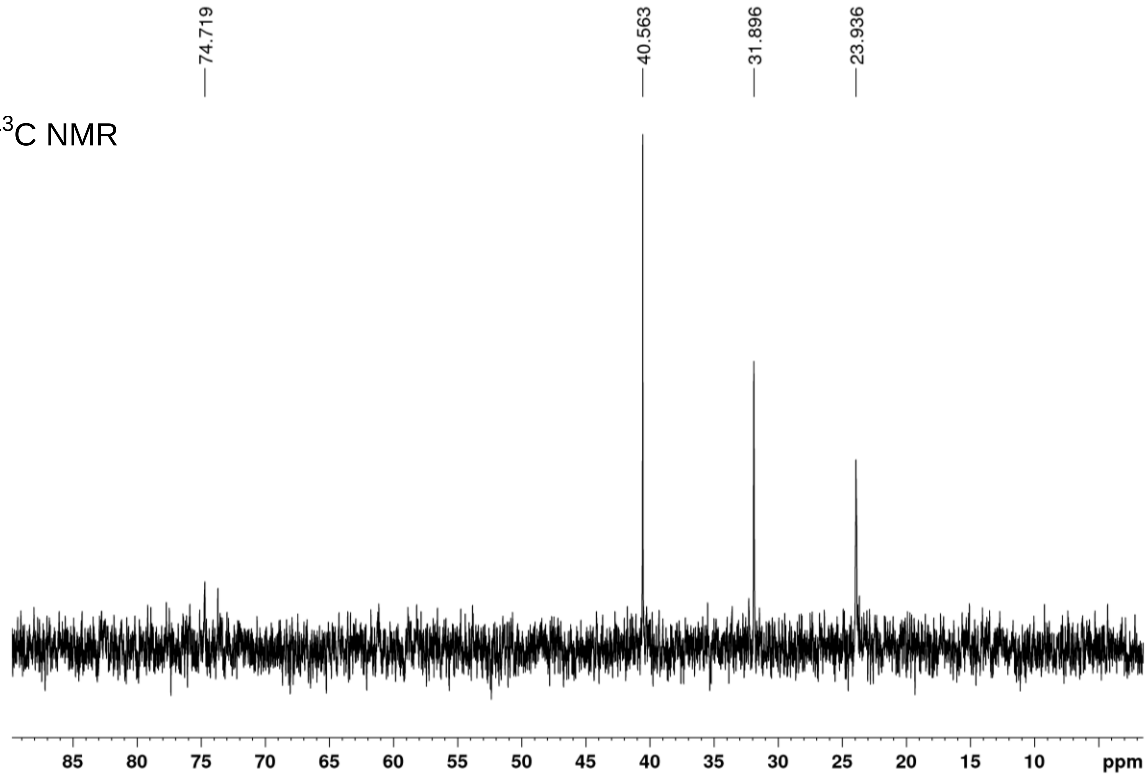

^{31}P NMR

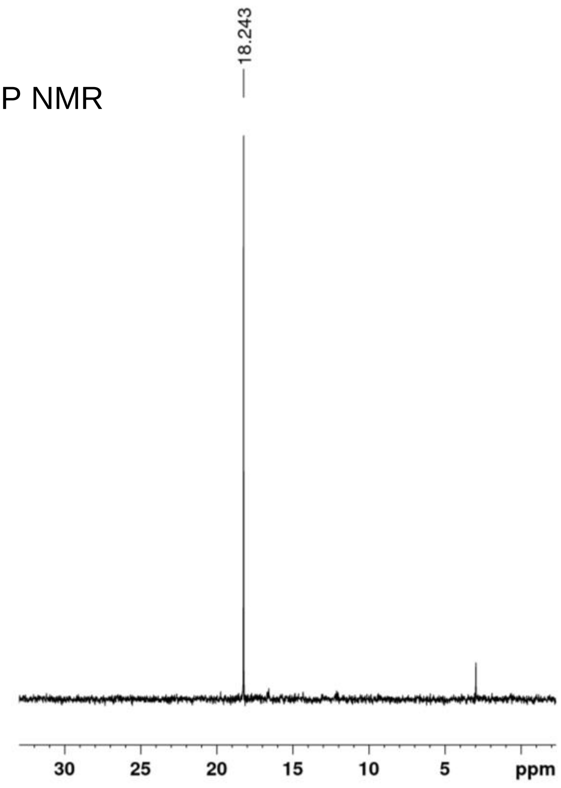

Figure S3. ^{1}H, ^{13}C and ^{31}P NMR of compound (**3**).

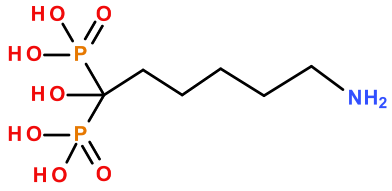

^{1}H NMR

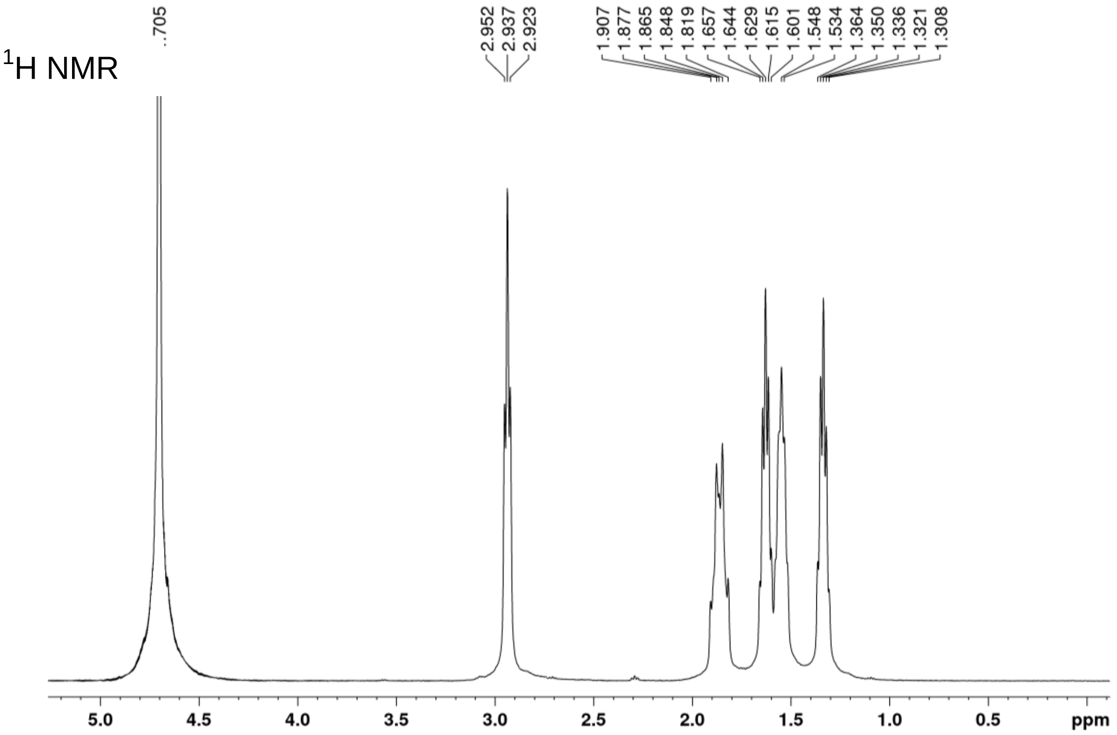

^{13}C NMR

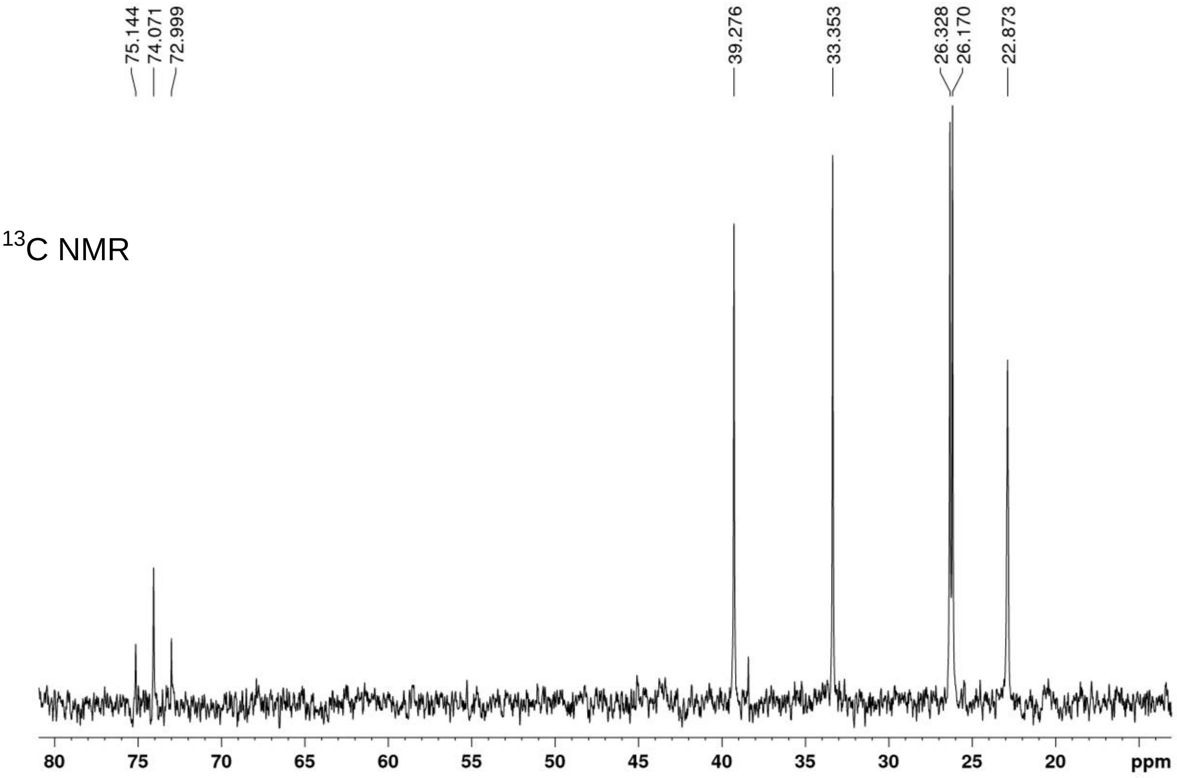

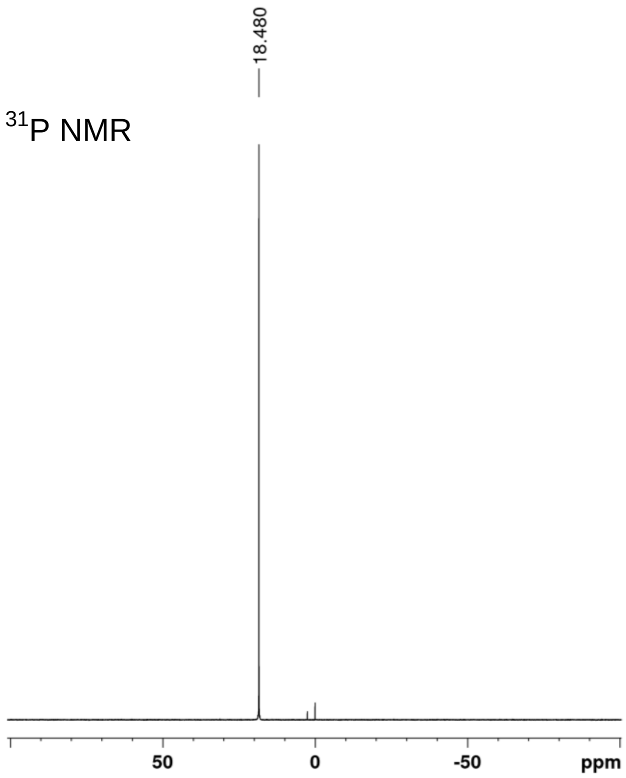
18.480
31P NMR
50
0
-50
ppm

Figure S4. ^{1}H NMR of compound (**4**).

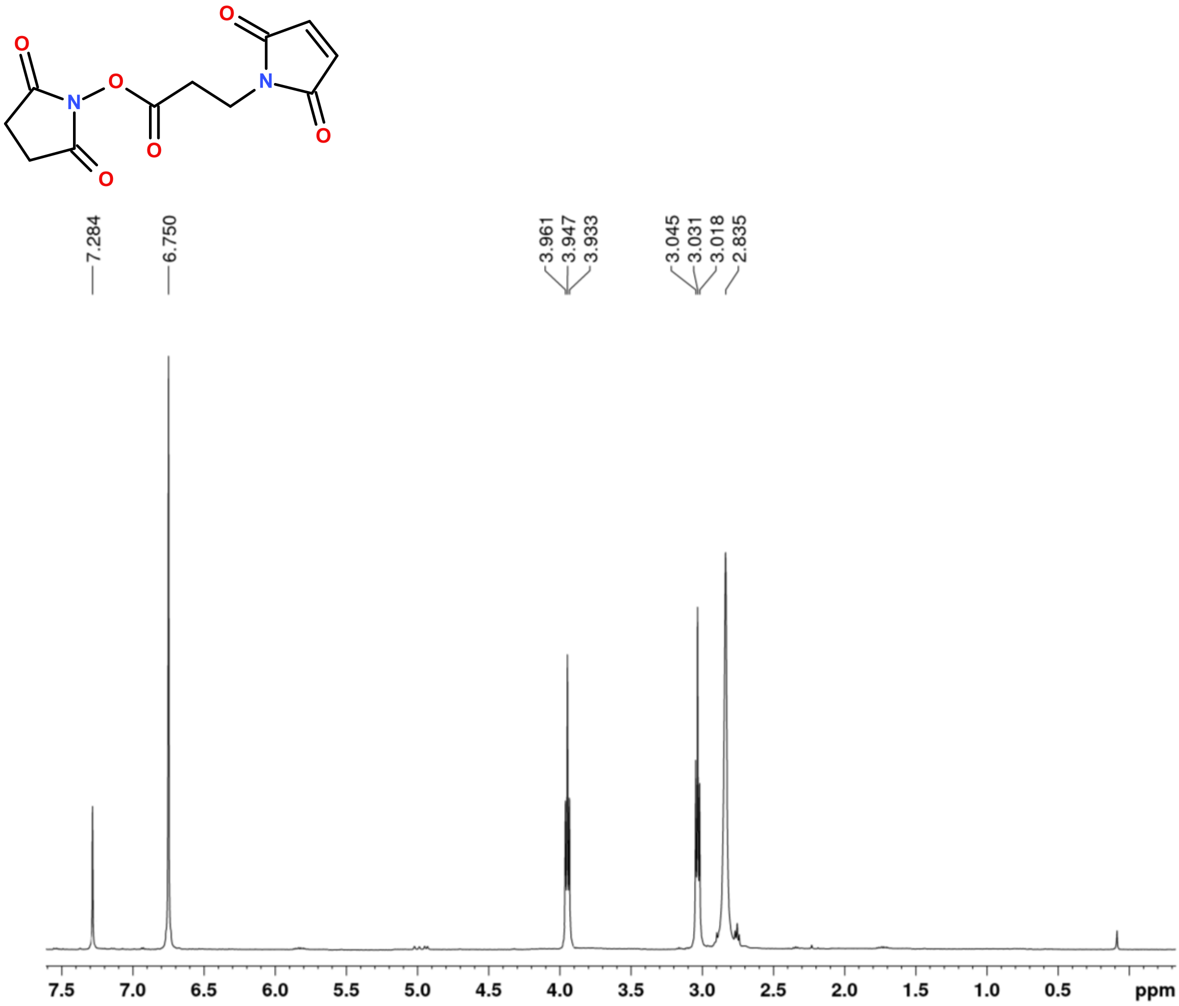

Figure S5. ^{1}H NMR of compound (**5**).

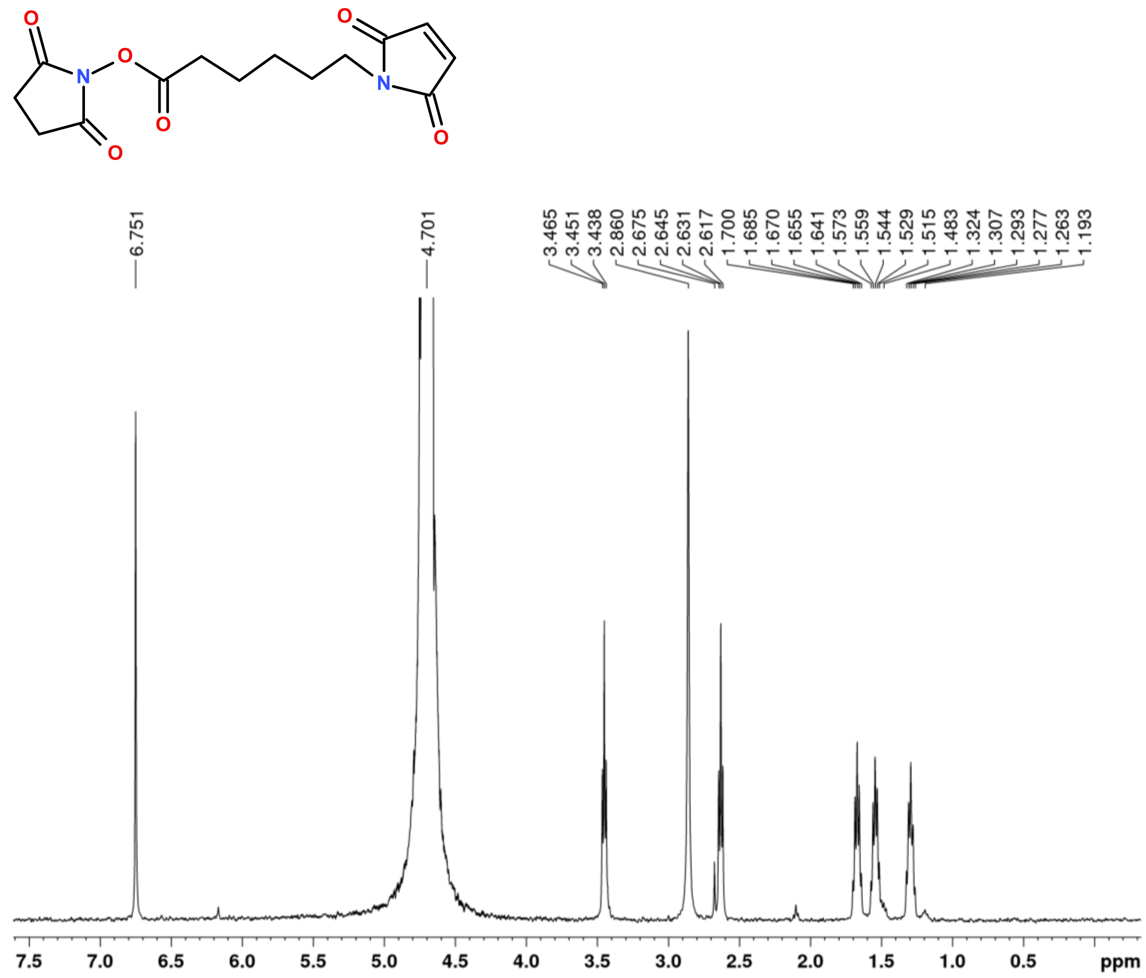

Figure S6. ^{1}H NMR of compound (**6**).

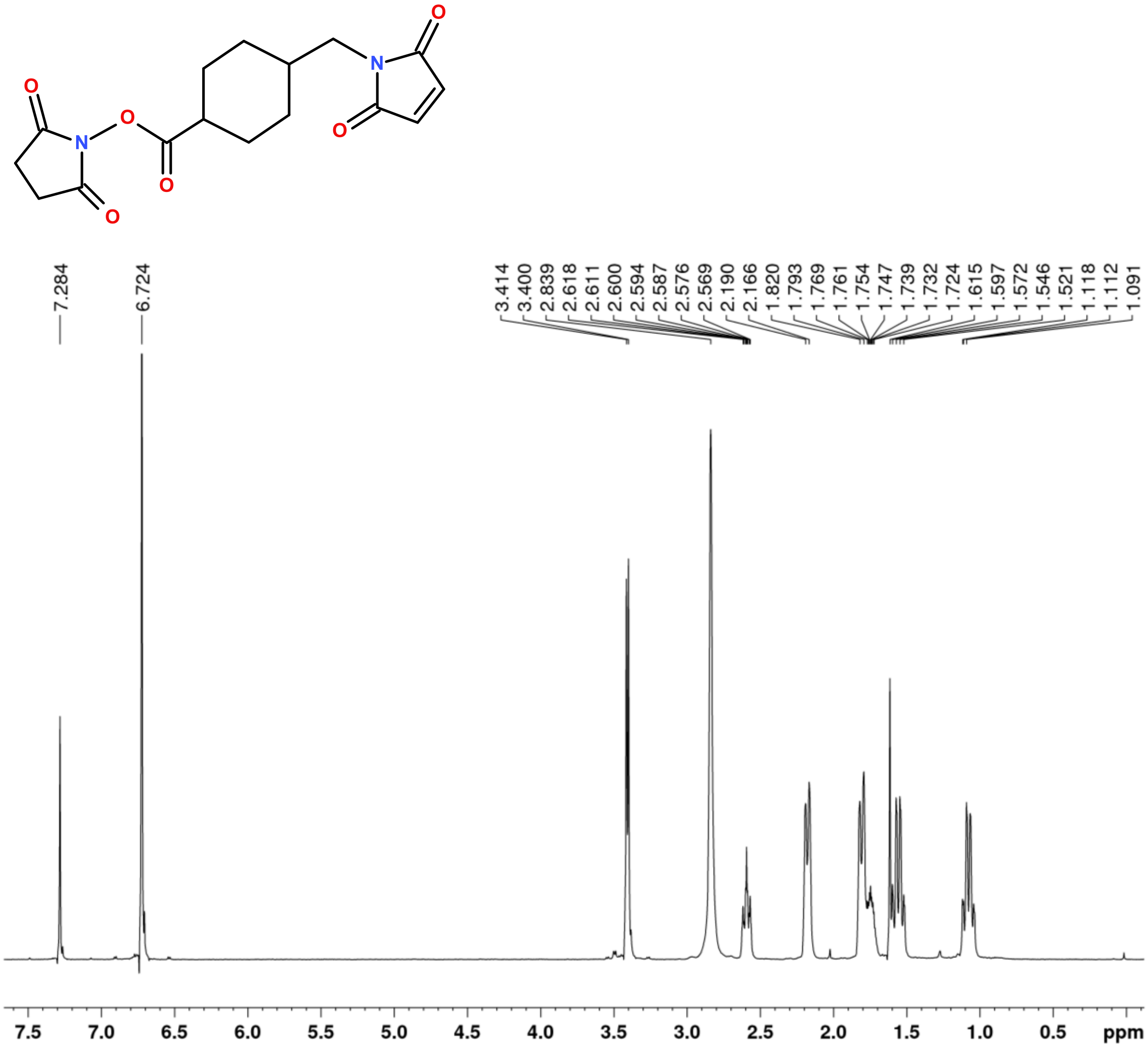

Figure S7. ^{1}H, ^{13}C and ^{31}P NMR of compound (**7**).

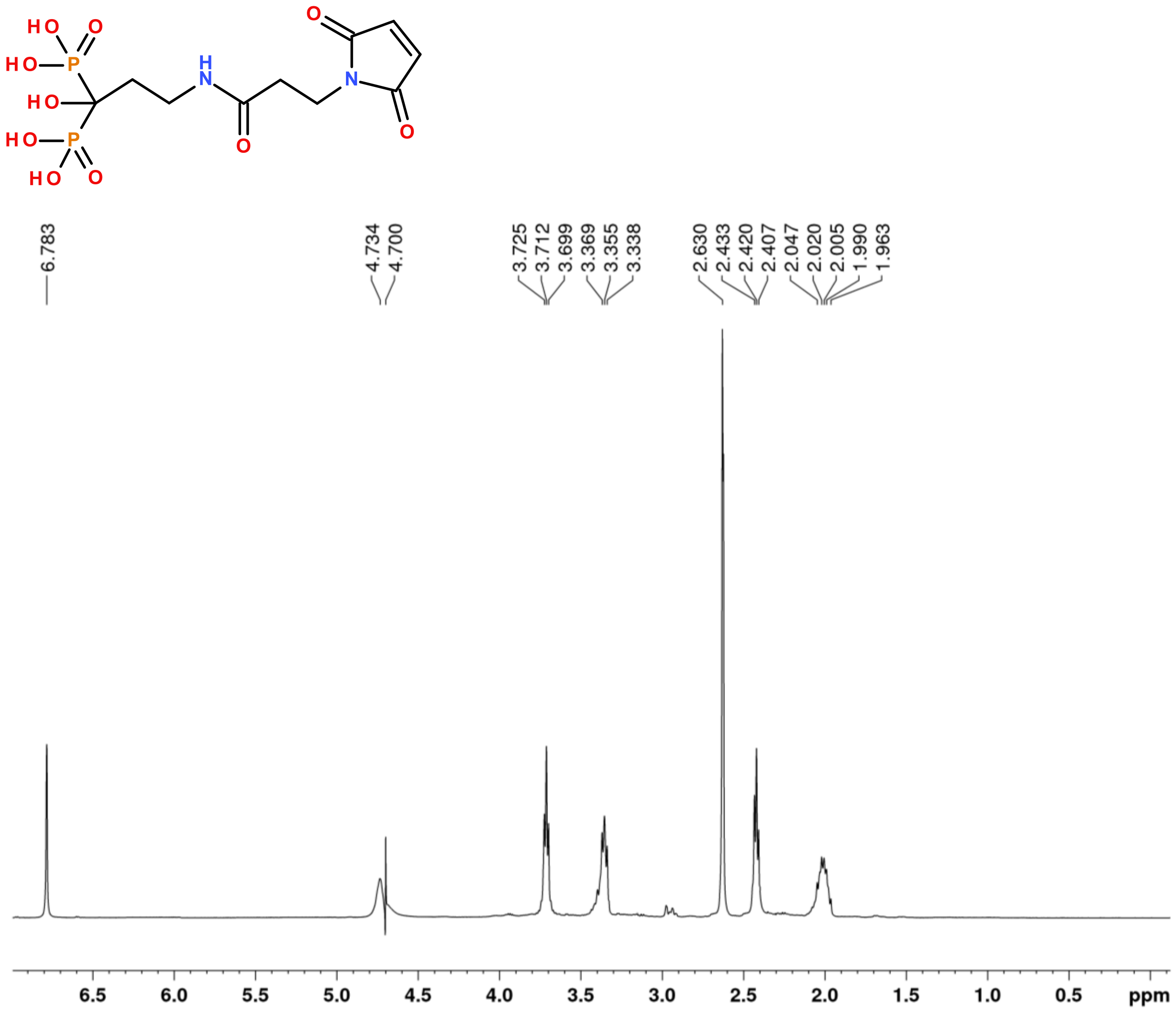

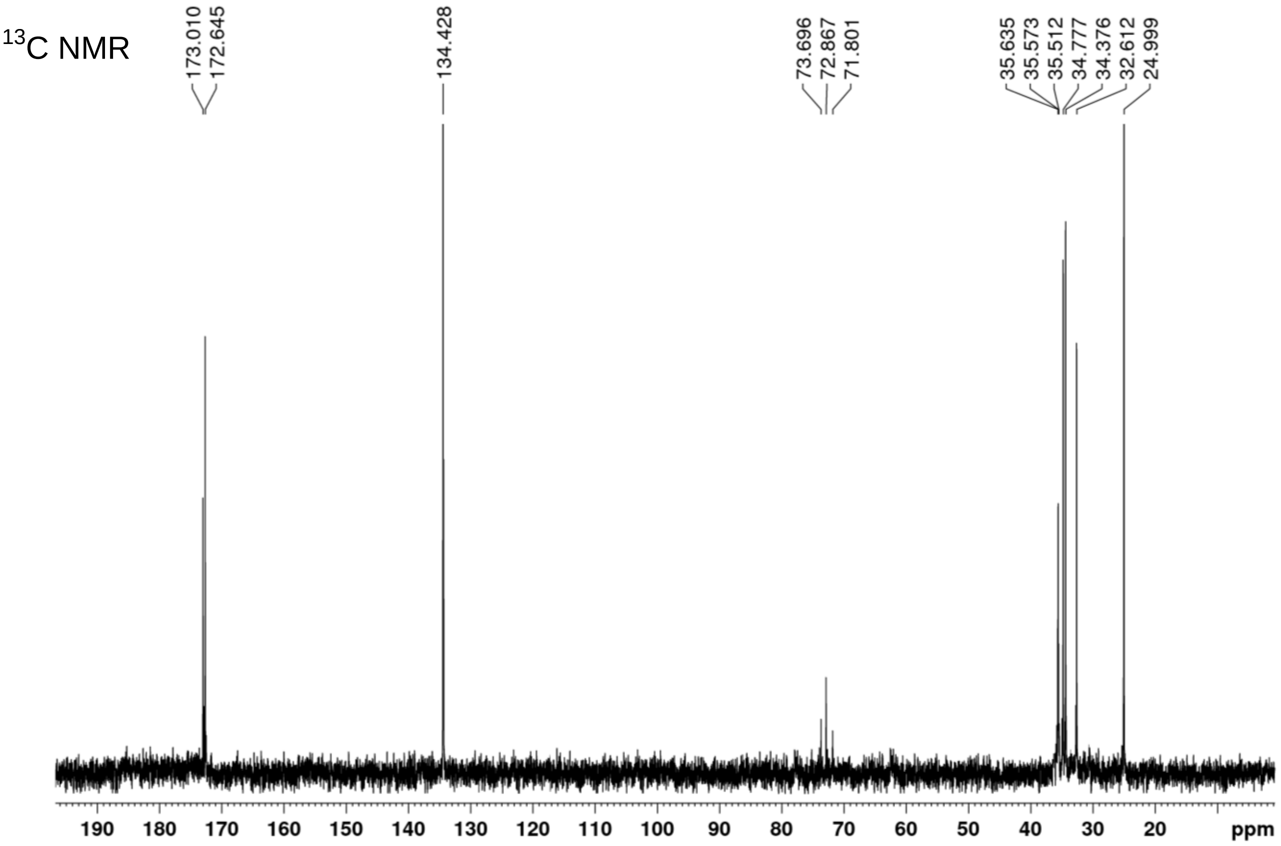

13C NMR
173.010
172.645
134.428
73.696
72.867
71.801
35.635
35.573
35.512
34.777
34.376
32.612
24.999
190 180 170 160 150 140 130 120 110 100 90 80 70 60 50 40 30 20 ppm

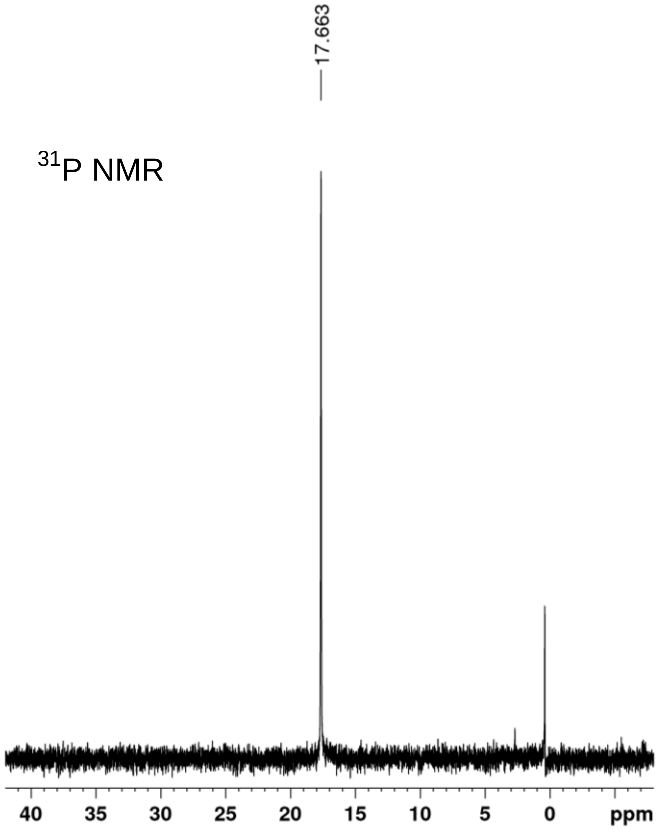

31P NMR
17.663
40 35 30 25 20 15 10 5 0 ppm

Figure S8. ¹H, ¹³C and ³¹P NMR of compound (**8**).

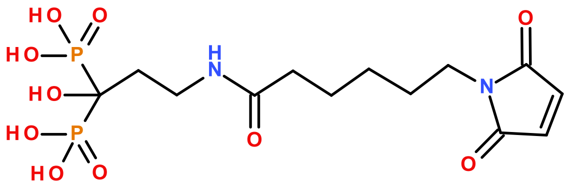

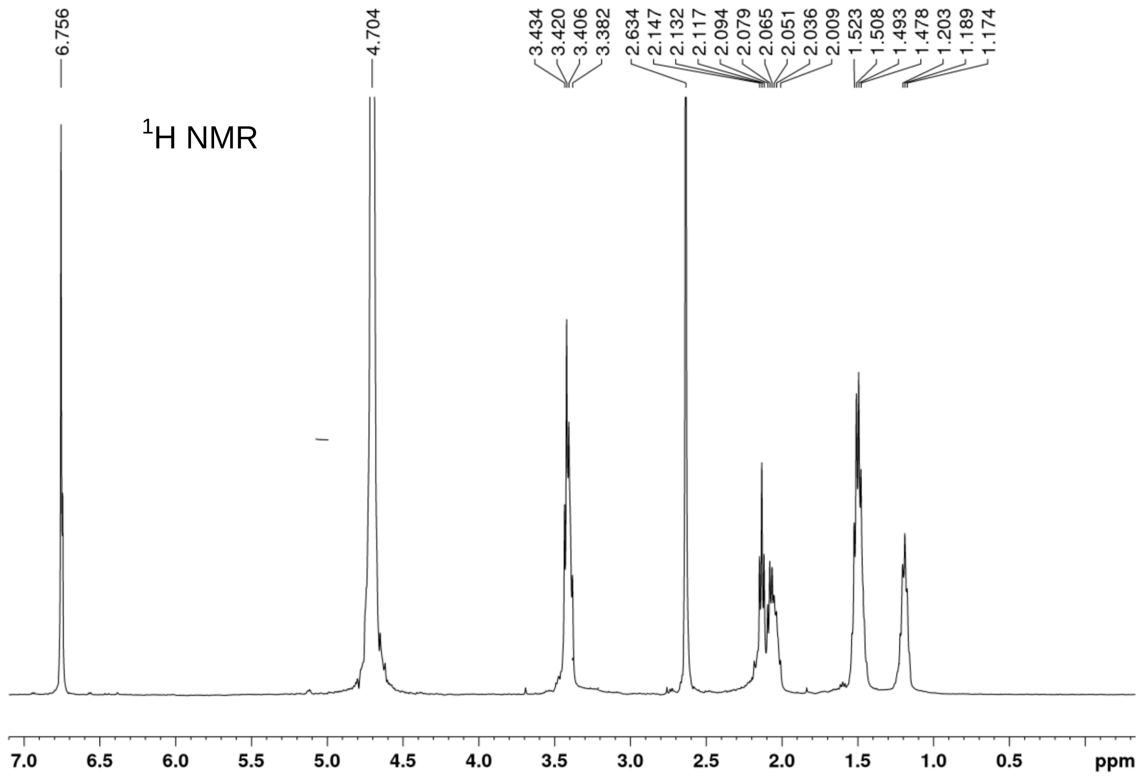

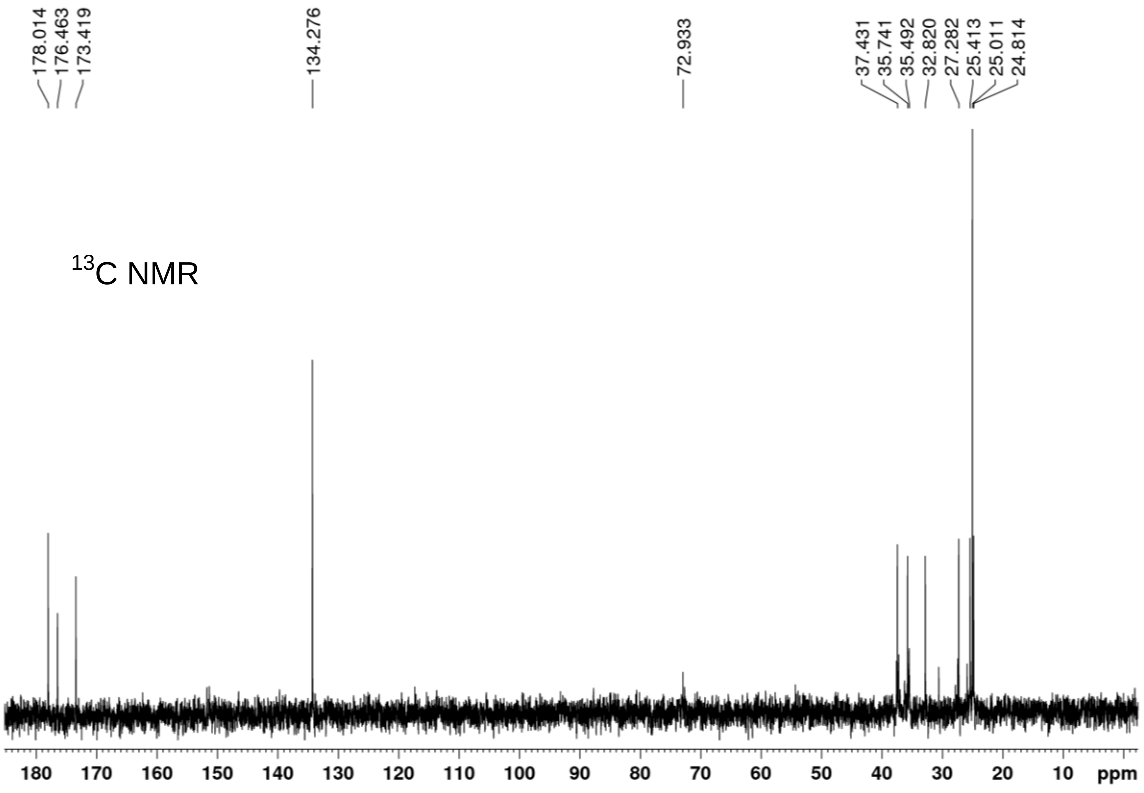

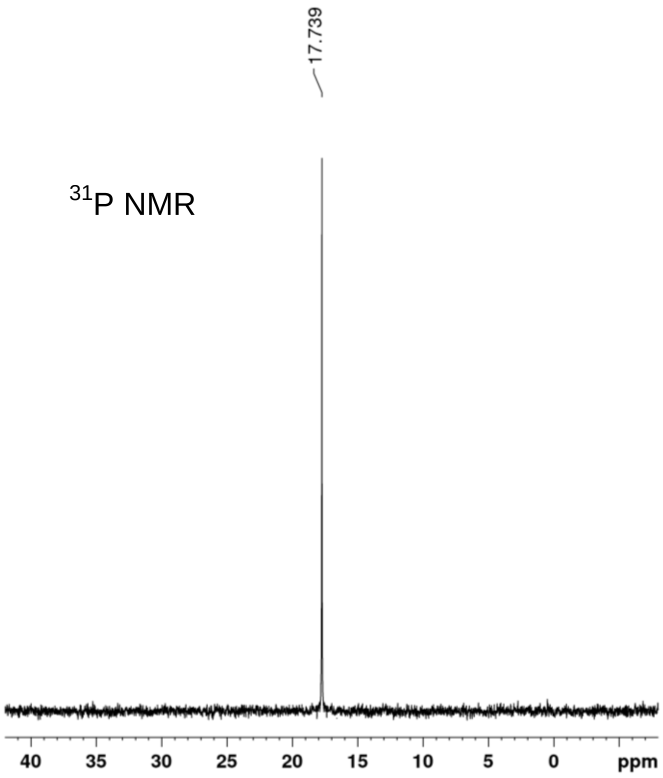
17.739
31P NMR
40
35
30
25
20
15
10
5
0
ppm

Figure S9. ^{1}H, ^{13}C and ^{31}P NMR of compound (**9**).

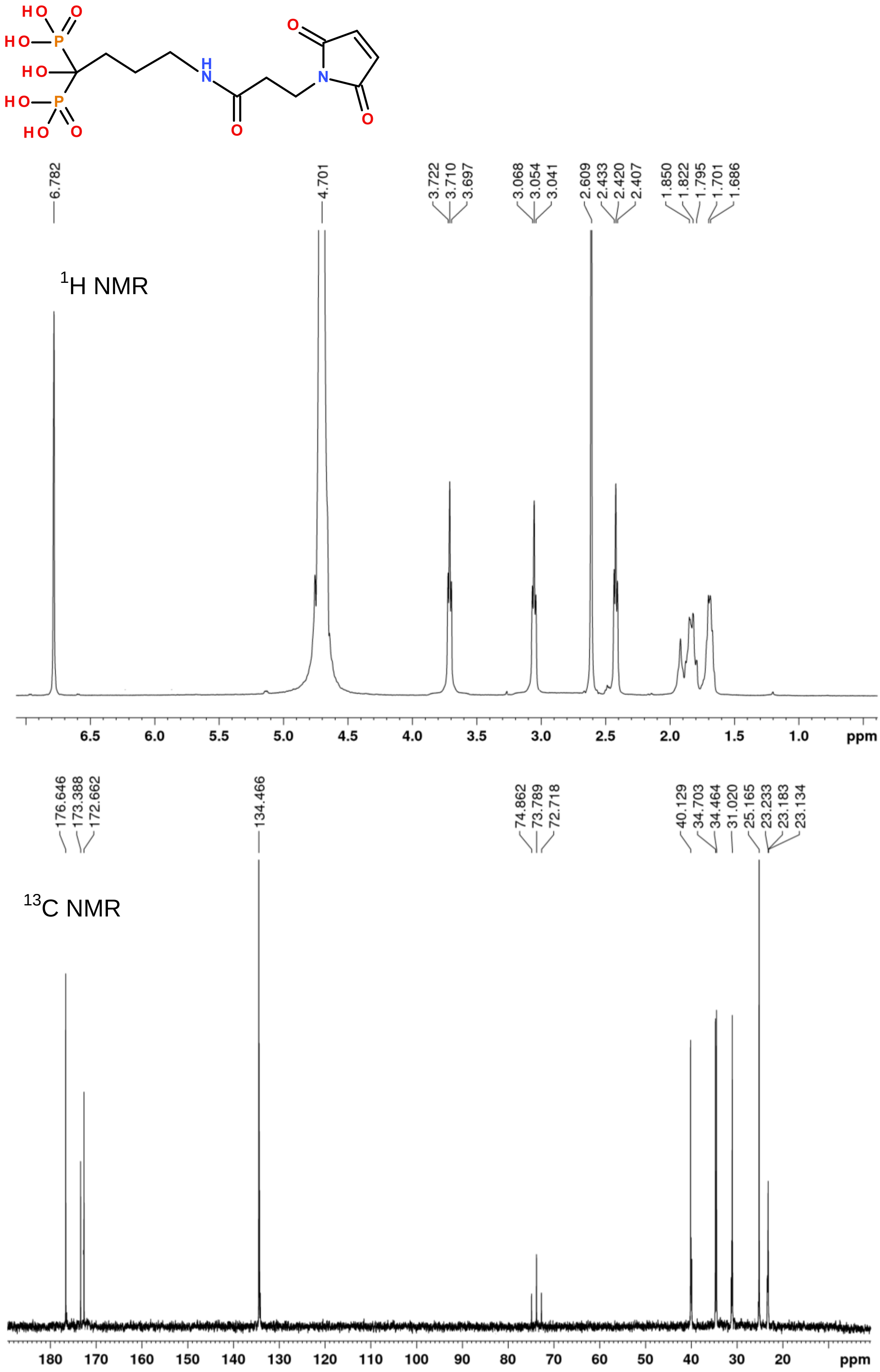

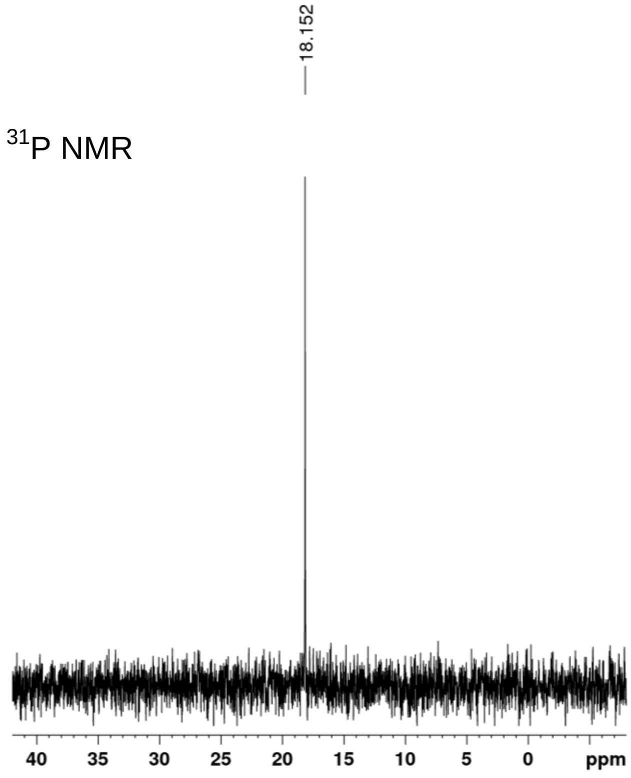
18.152
^{31}P NMR
40
35
30
25
20
15
10
5
0
ppm

Figure S10. ^{1}H, ^{13}C and ^{31}P NMR of compound (**10**).

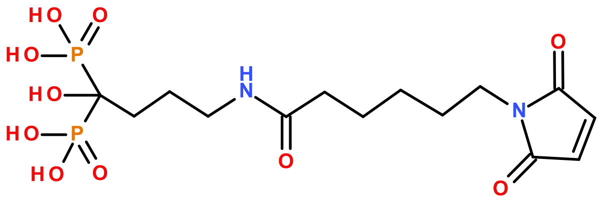

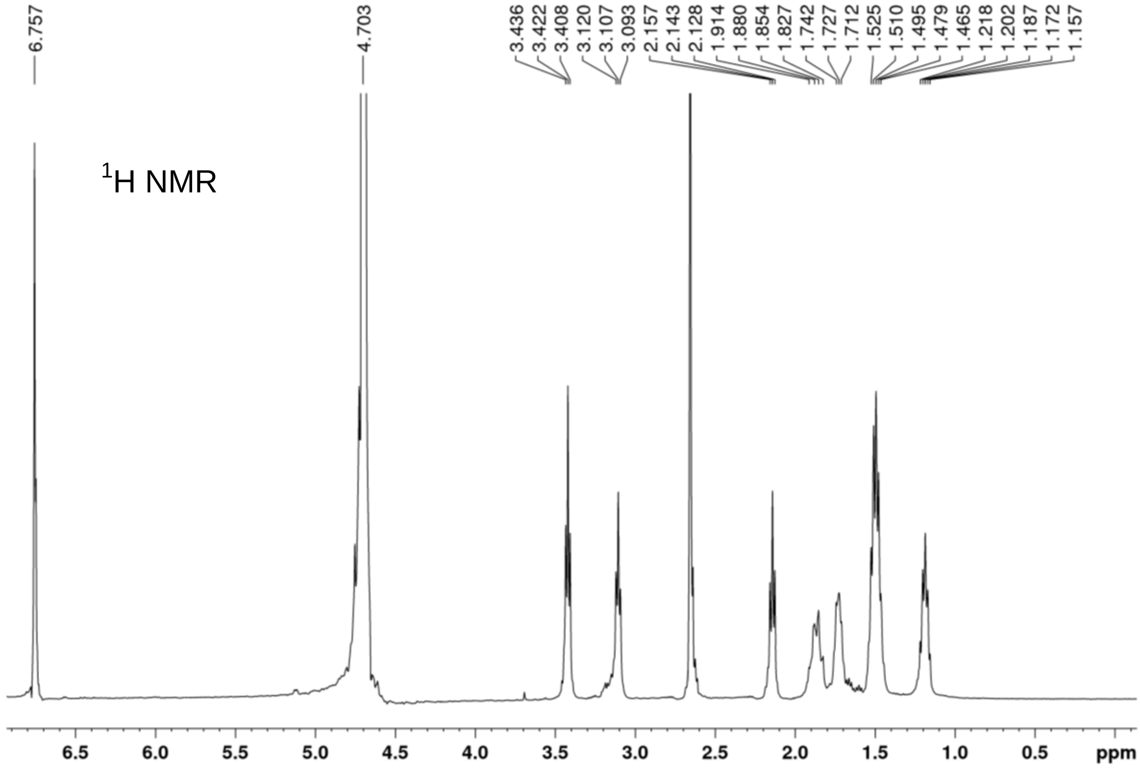

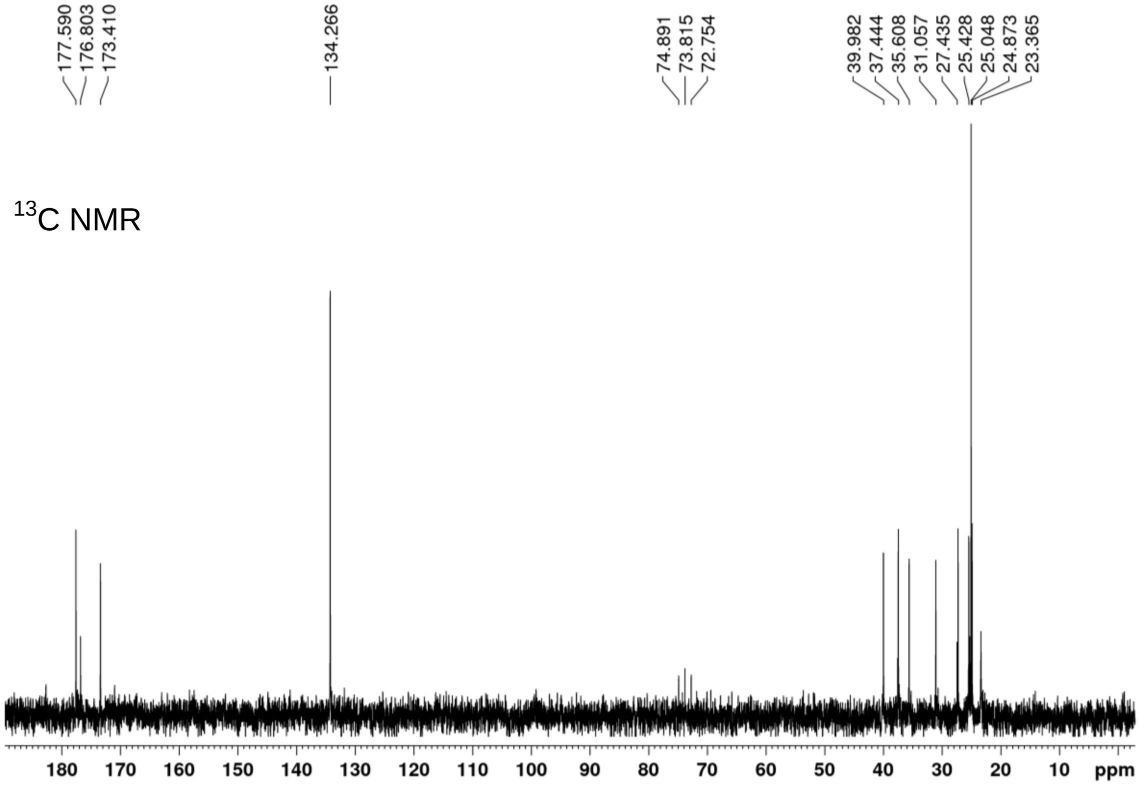

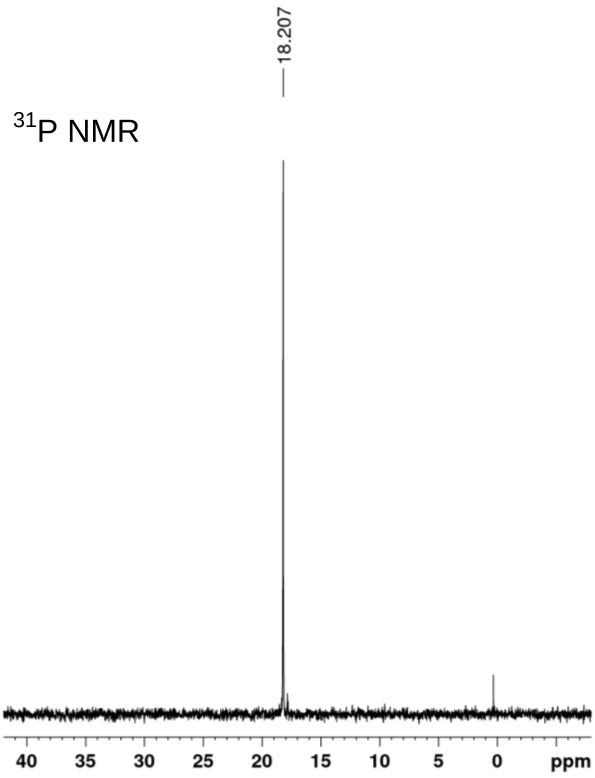
31P NMR
18.207
40 35 30 25 20 15 10 5 0 ppm

Figure S11. ^{1}H, ^{13}C and ^{31}P NMR of compound (**11**).

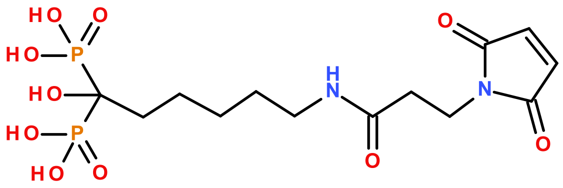

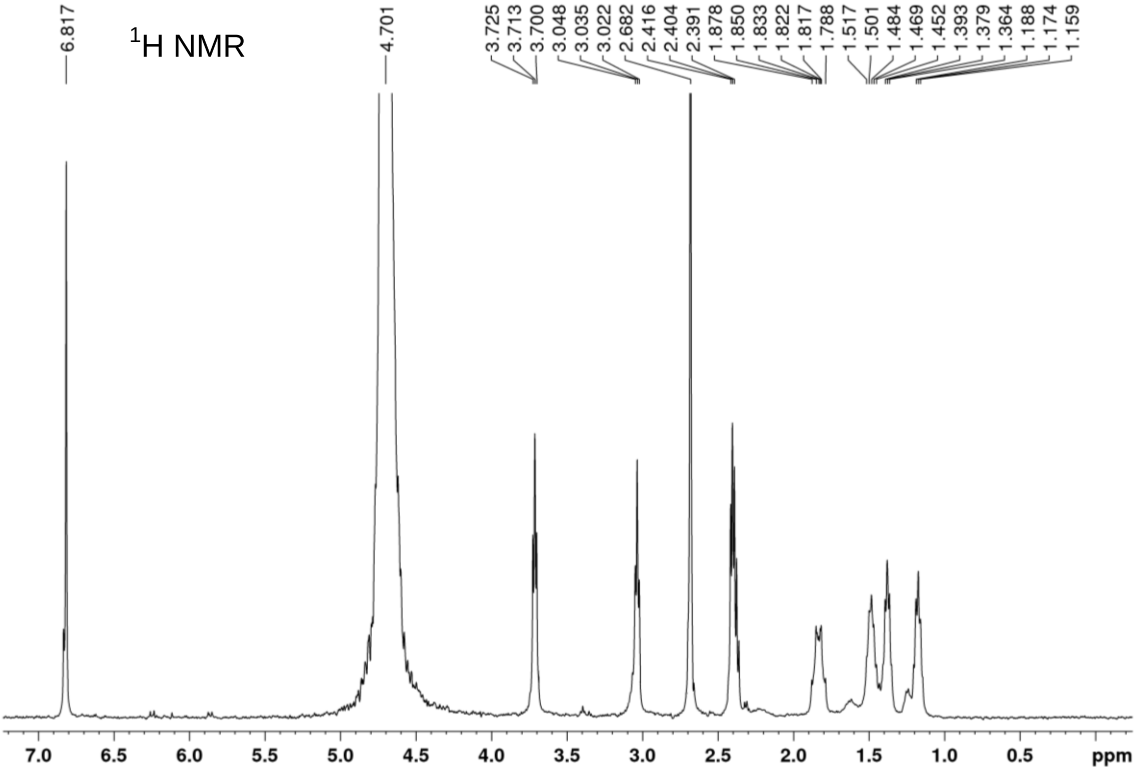

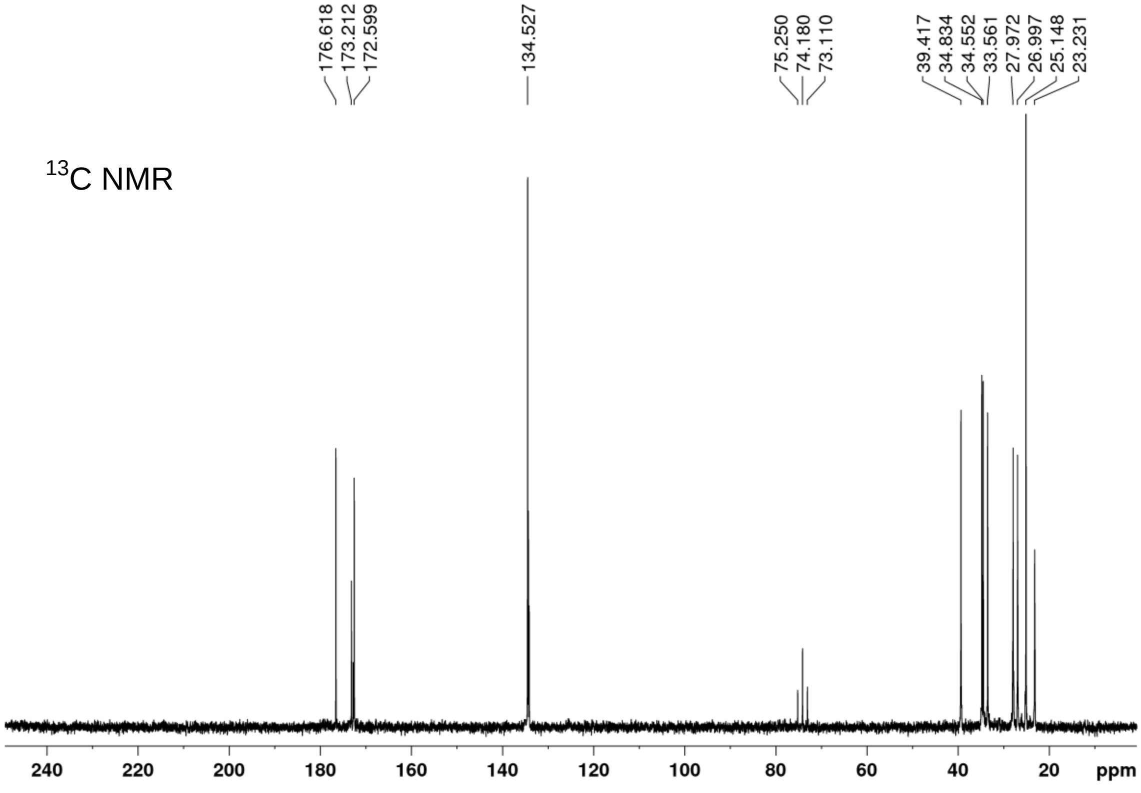

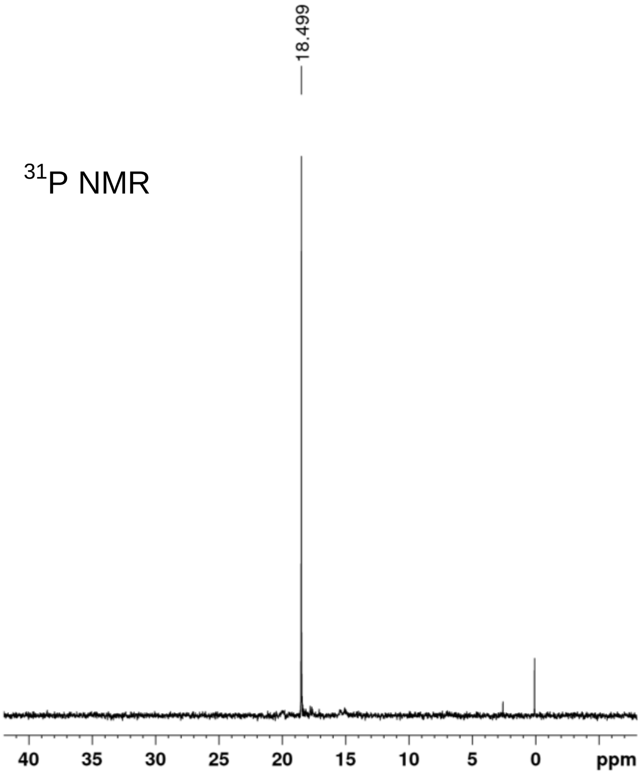

18.499
31P NMR
40
35
30
25
20
15
10
5
0
ppm

Figure S12. ^{1}H, ^{13}C and ^{31}P NMR of compound (**12**).

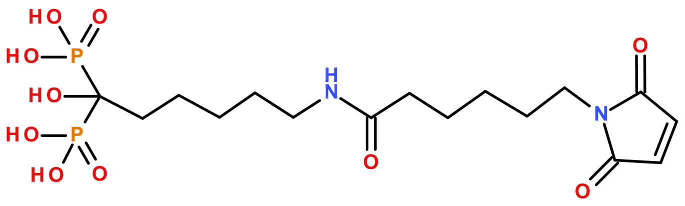

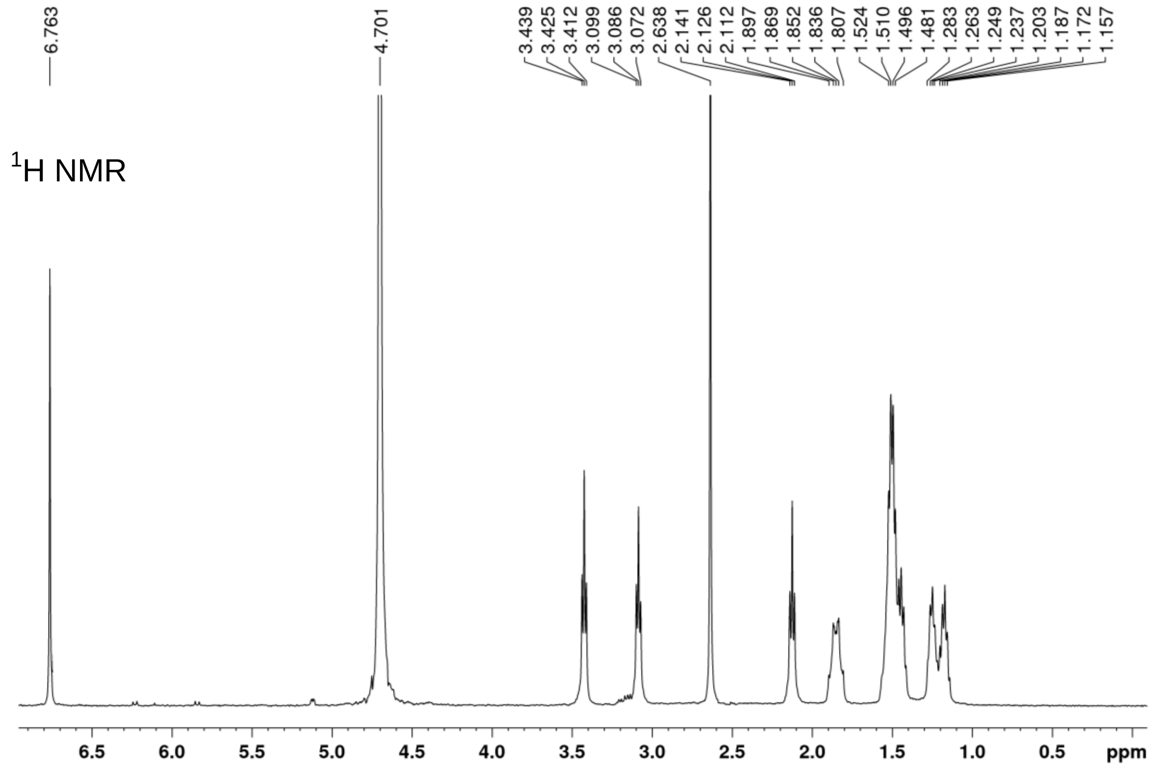

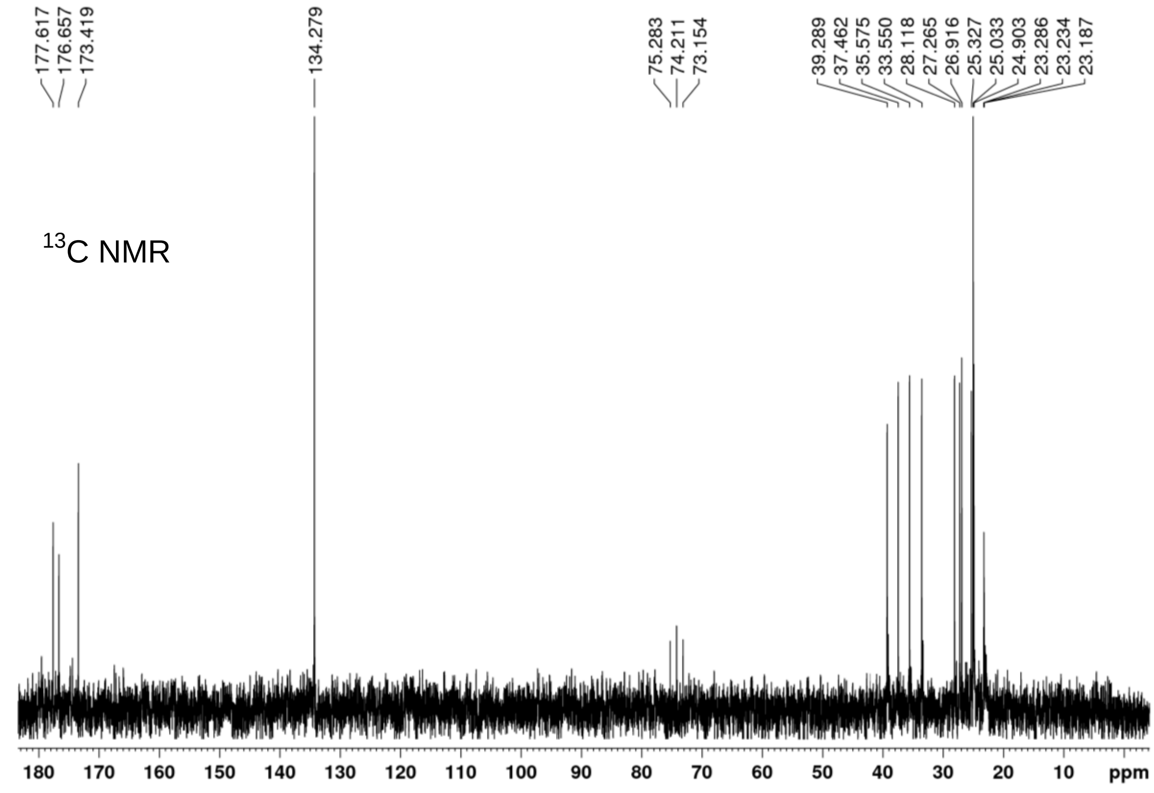

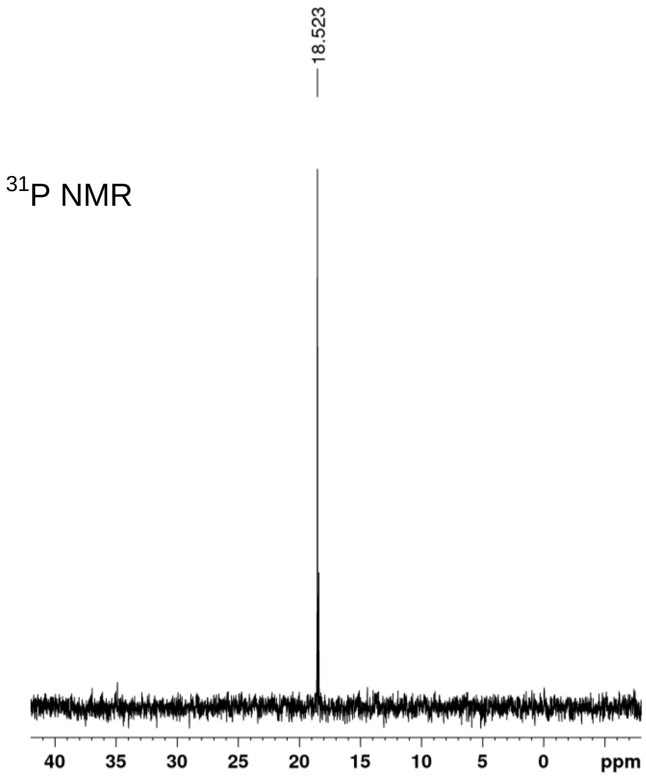
18.523
³¹P NMR
40
35
30
25
20
15
10
5
0
ppm

Figure S13. ^{1}H, ^{13}C and ^{31}P NMR of compound (**13**).

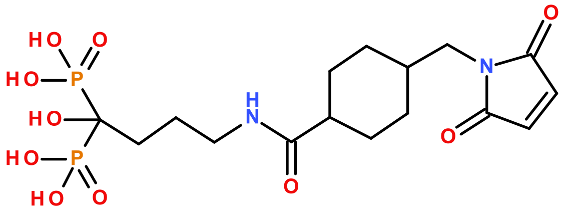

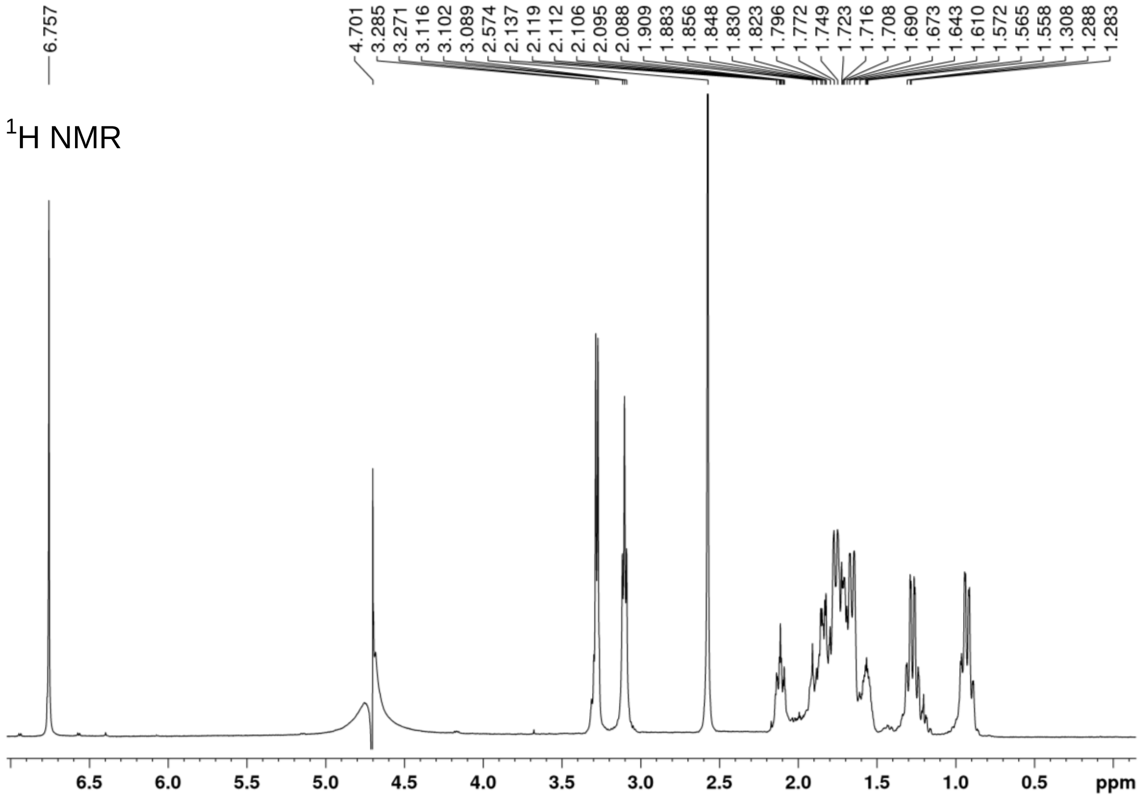

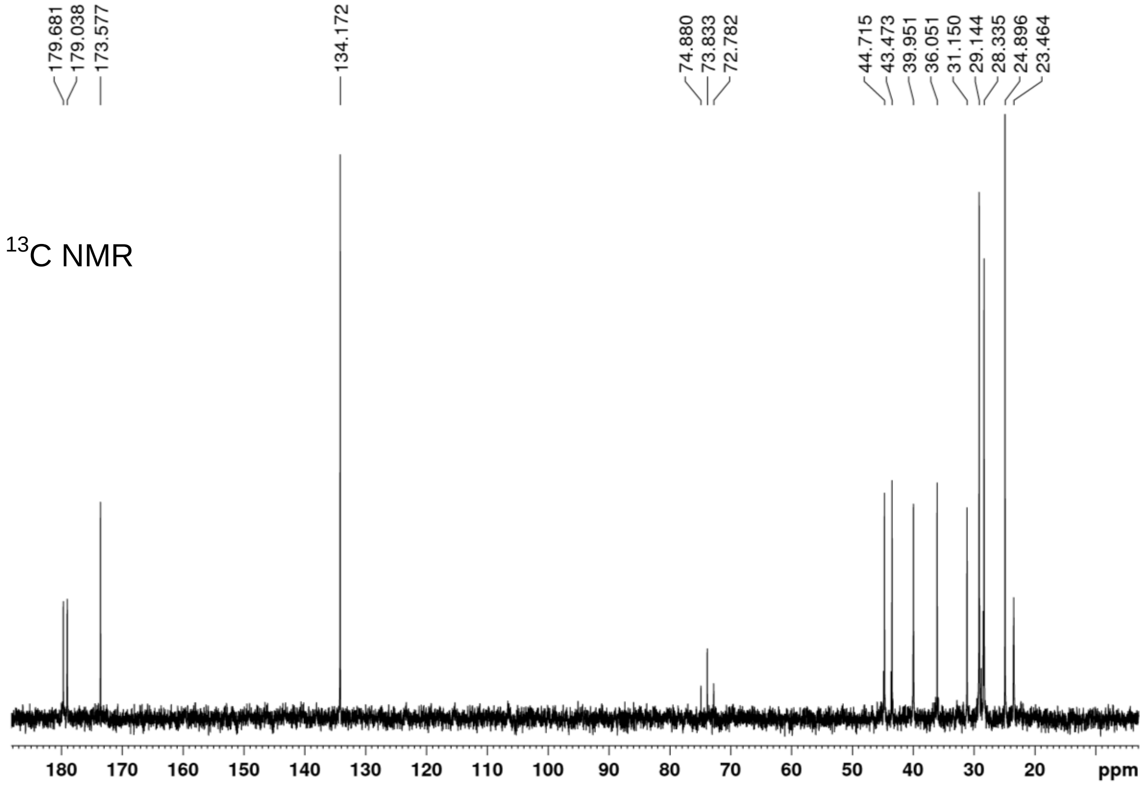

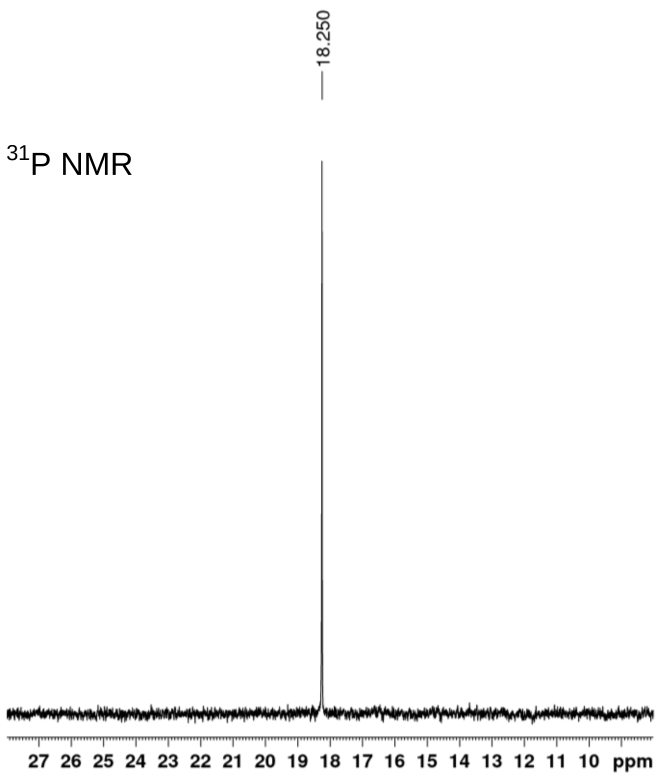
18.250
^{31}P NMR
27 26 25 24 23 22 21 20 19 18 17 16 15 14 13 12 11 10 ppm

Figure S14. ^{1}H, ^{13}C and ^{31}P NMR of compound (**14**).

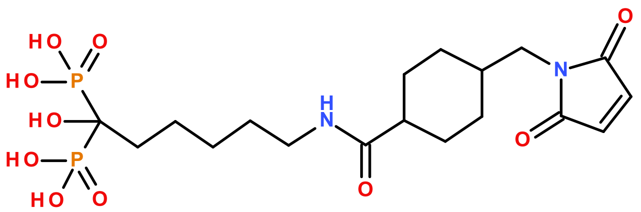

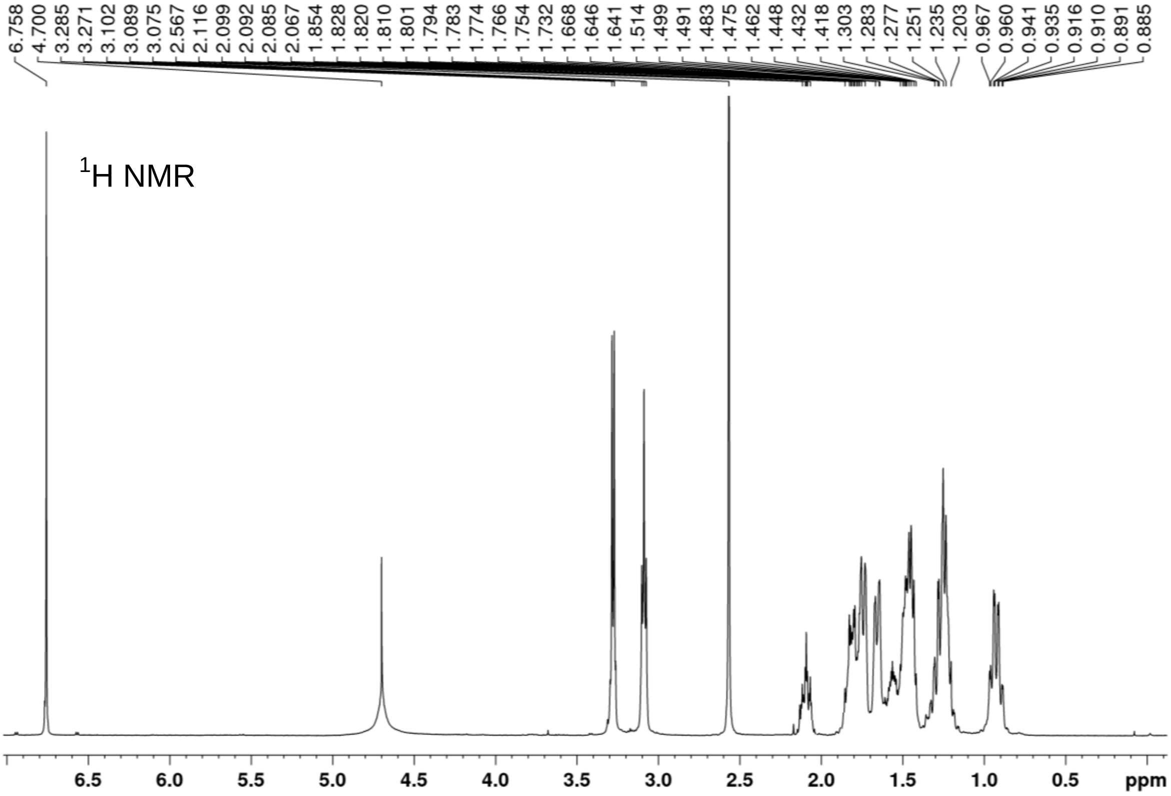

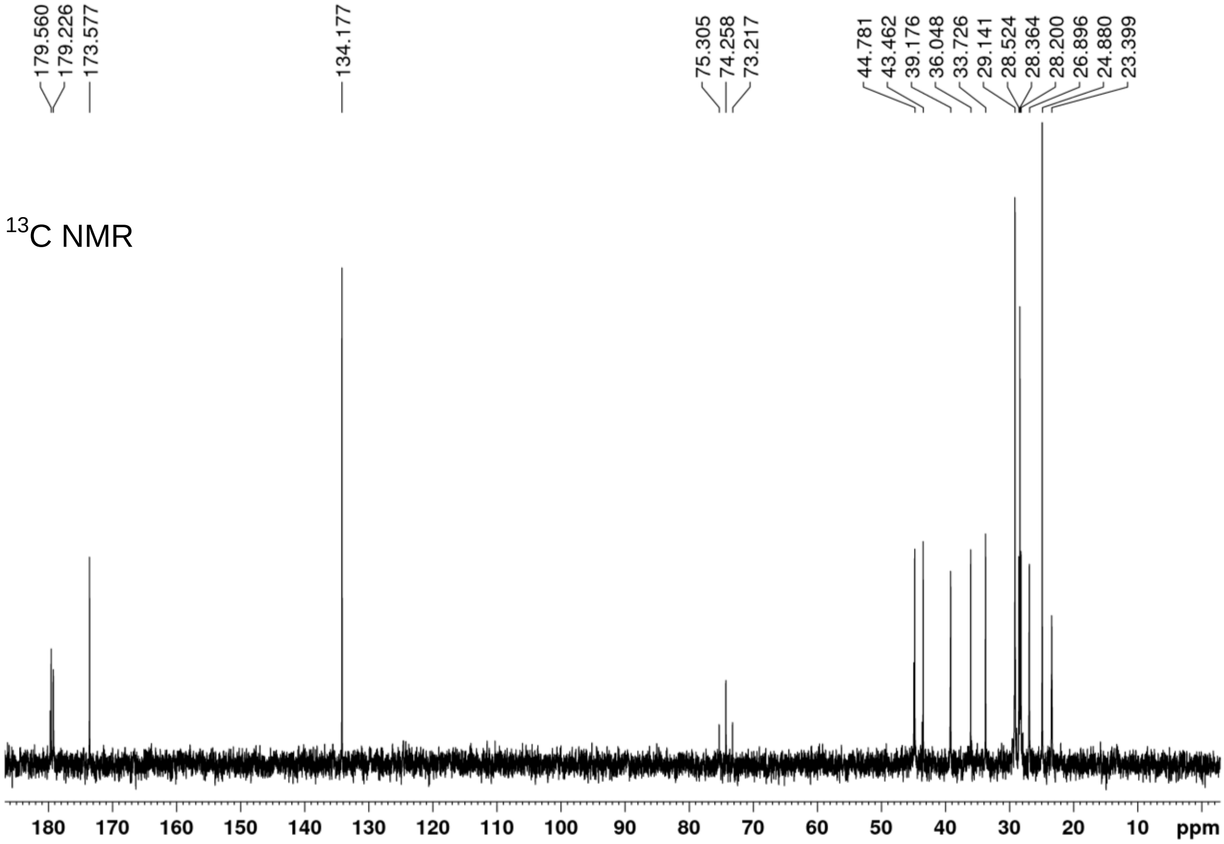
179.560
179.226
173.577
134.177
75.305
74.258
73.217
44.781
43.462
39.176
36.048
33.726
29.141
28.524
28.364
28.200
26.896
24.880
23.399
^{13}C NMR
180 170 160 150 140 130 120 110 100 90 80 70 60 50 40 30 20 10 ppm

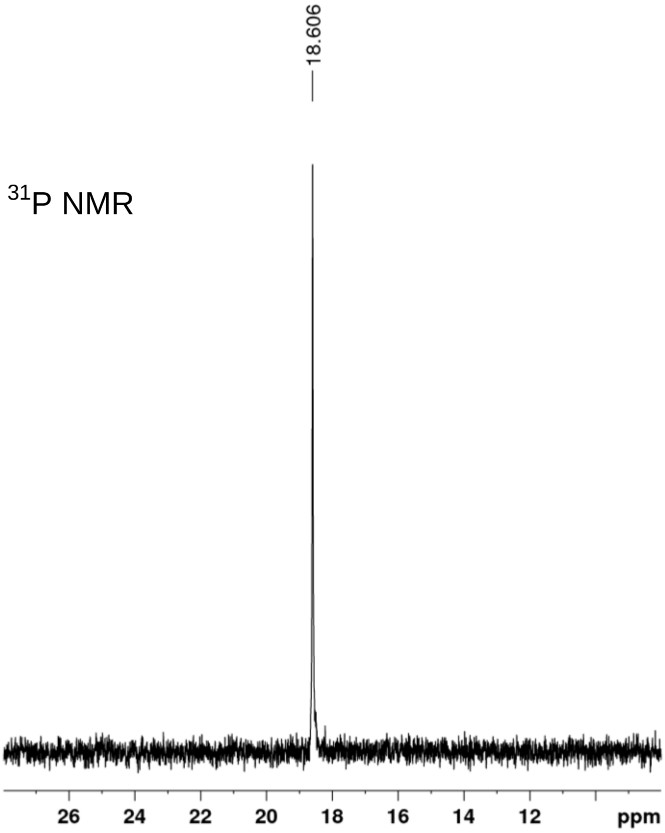
18.606
^{31}P NMR
26 24 22 20 18 16 14 12 ppm

Figure S15. ^{1}H and ^{31}P NMR of compound (**15**).

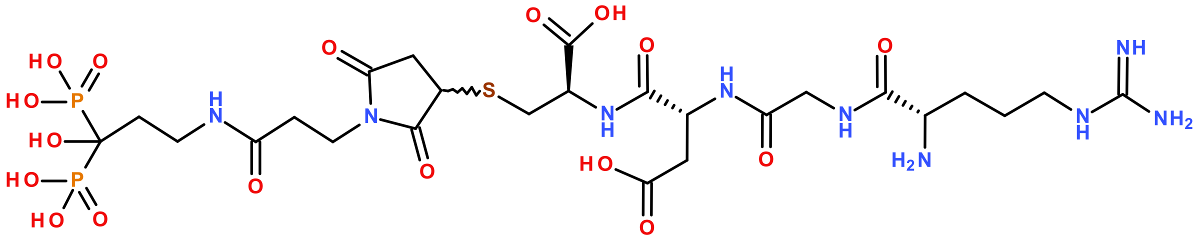

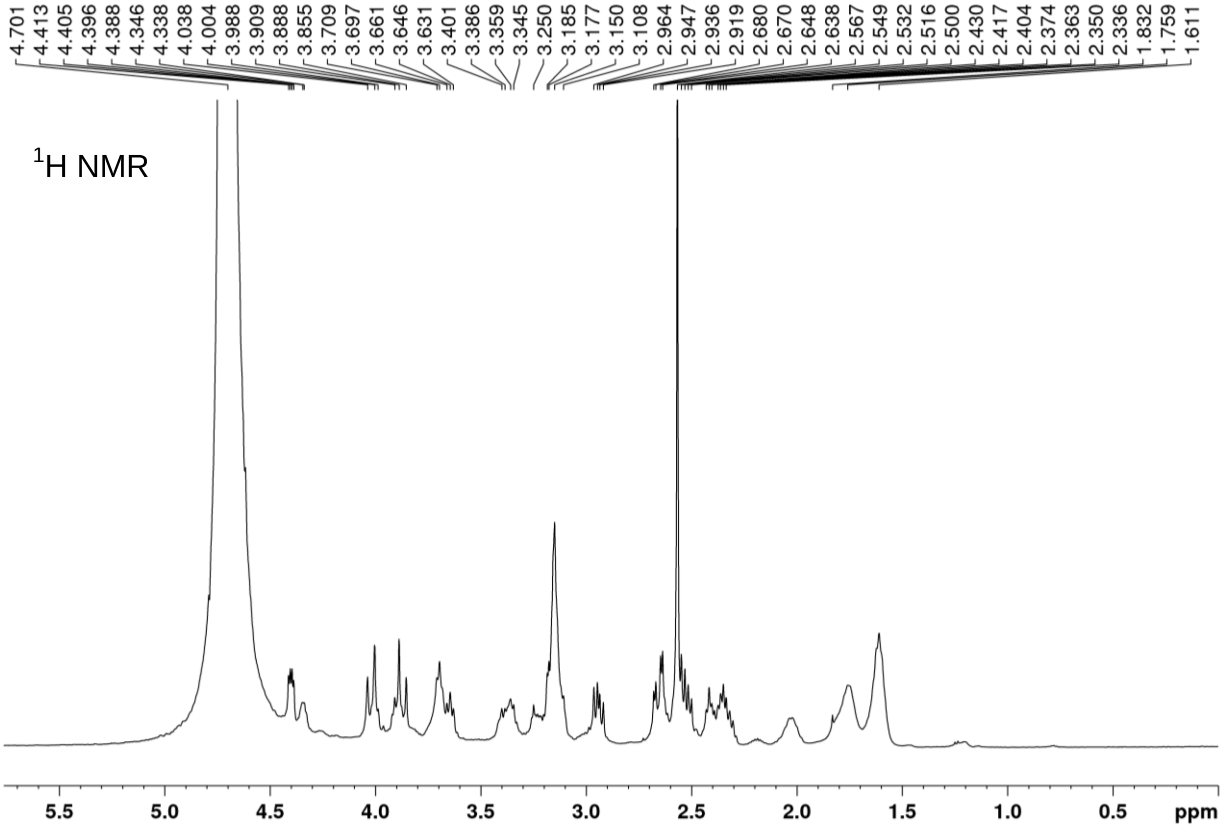

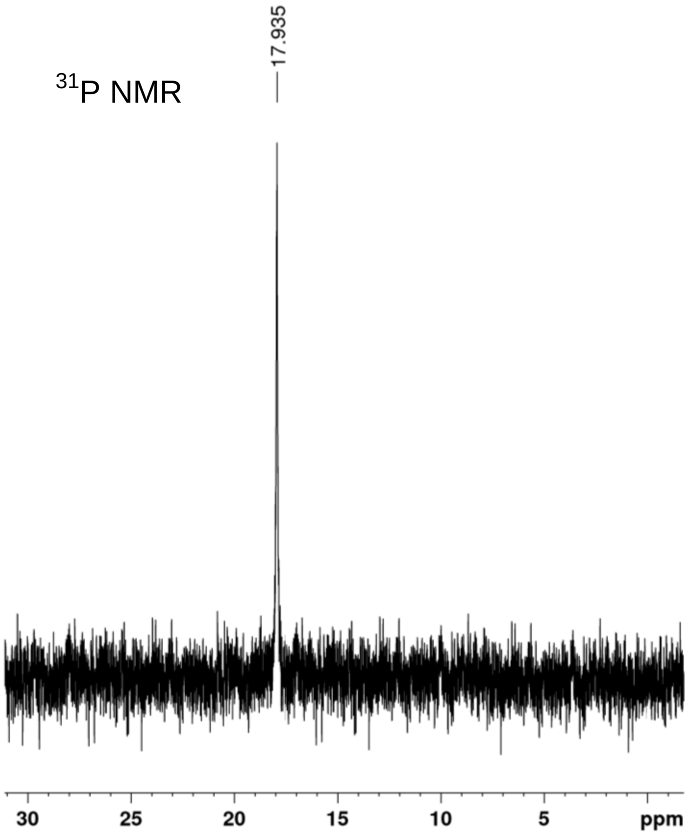

Figure S16. MALDI TOF/TOF of compound (**15**).

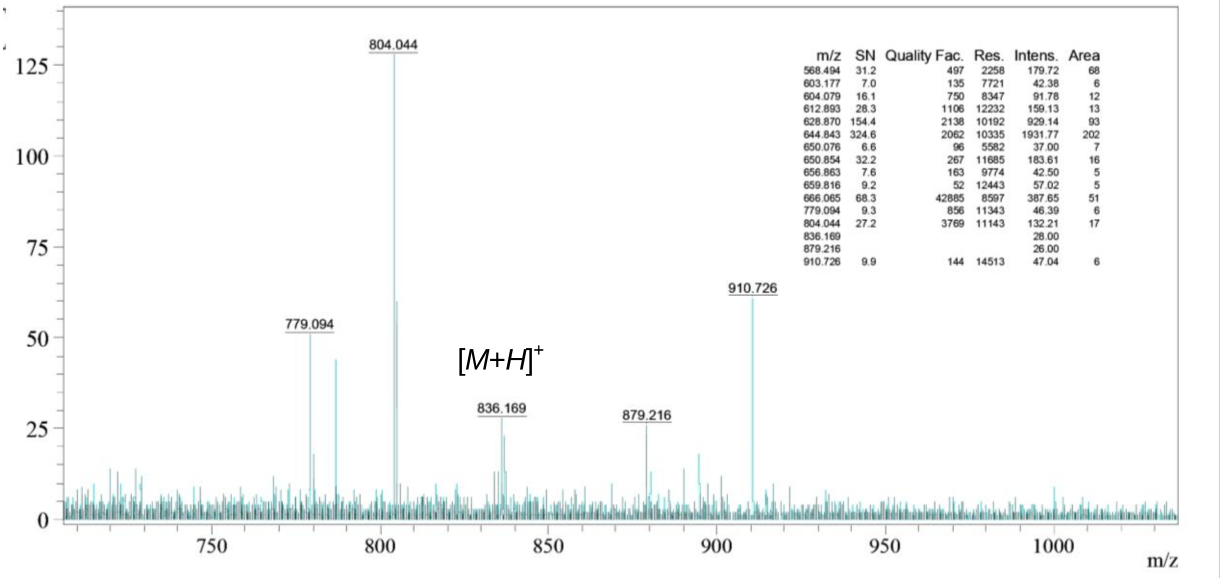

m/z	SN	Quality Fac.	Res.	Intens.	Area
568.494	31.2	497	2258	179.72	68
603.177	7.0	135	7721	42.38	6
604.079	16.1	750	8347	91.78	12
612.893	28.3	1106	12232	159.13	13
628.870	154.4	2138	10192	929.14	93
644.843	324.6	2062	10335	1931.77	202
650.076	6.6	96	5582	37.00	7
650.854	32.2	267	11685	183.61	16
656.863	7.6	163	9774	42.50	5
659.816	9.2	52	12443	57.02	5
666.065	68.3	42885	8597	387.65	51
779.094	9.3	856	11343	46.39	6
804.044	27.2	3769	11143	132.21	17
836.169				28.00	
879.216				26.00	
910.726	9.9	144	14513	47.04	6

Figure S17 ^{1}H and ^{31}P NMR of compound (**17**).

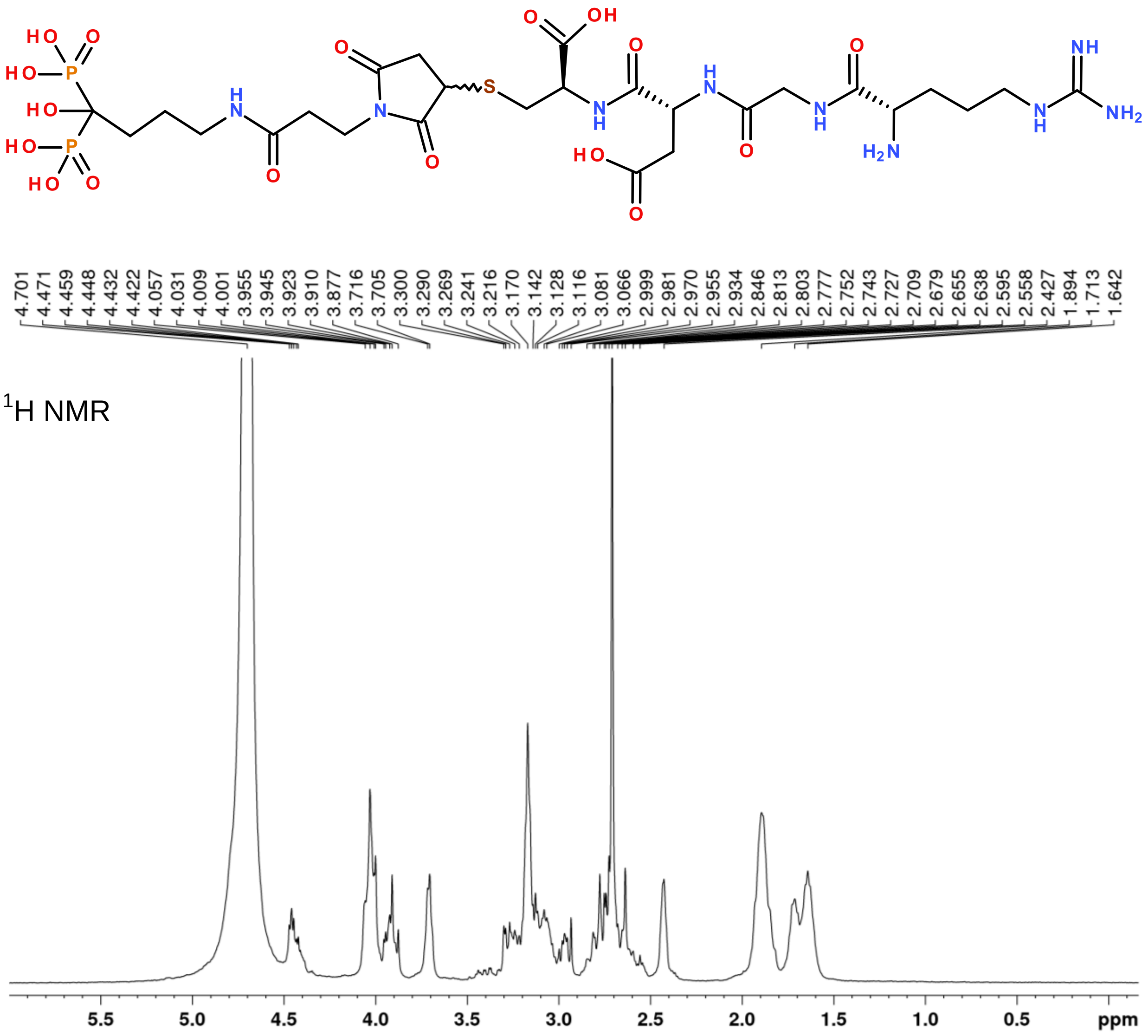

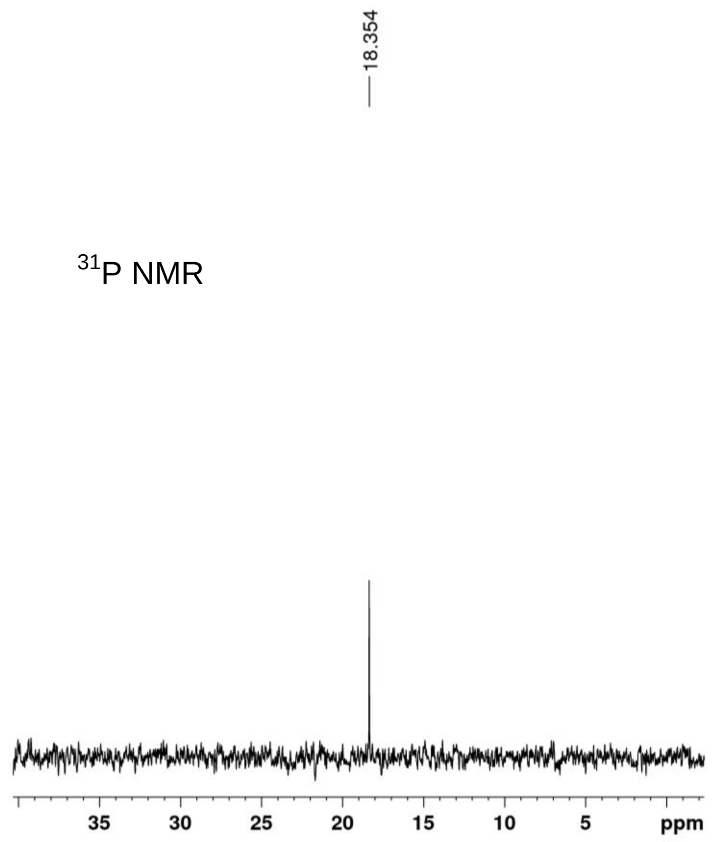

Figure S18. MALDI TOF/TOF of compound (**17**).

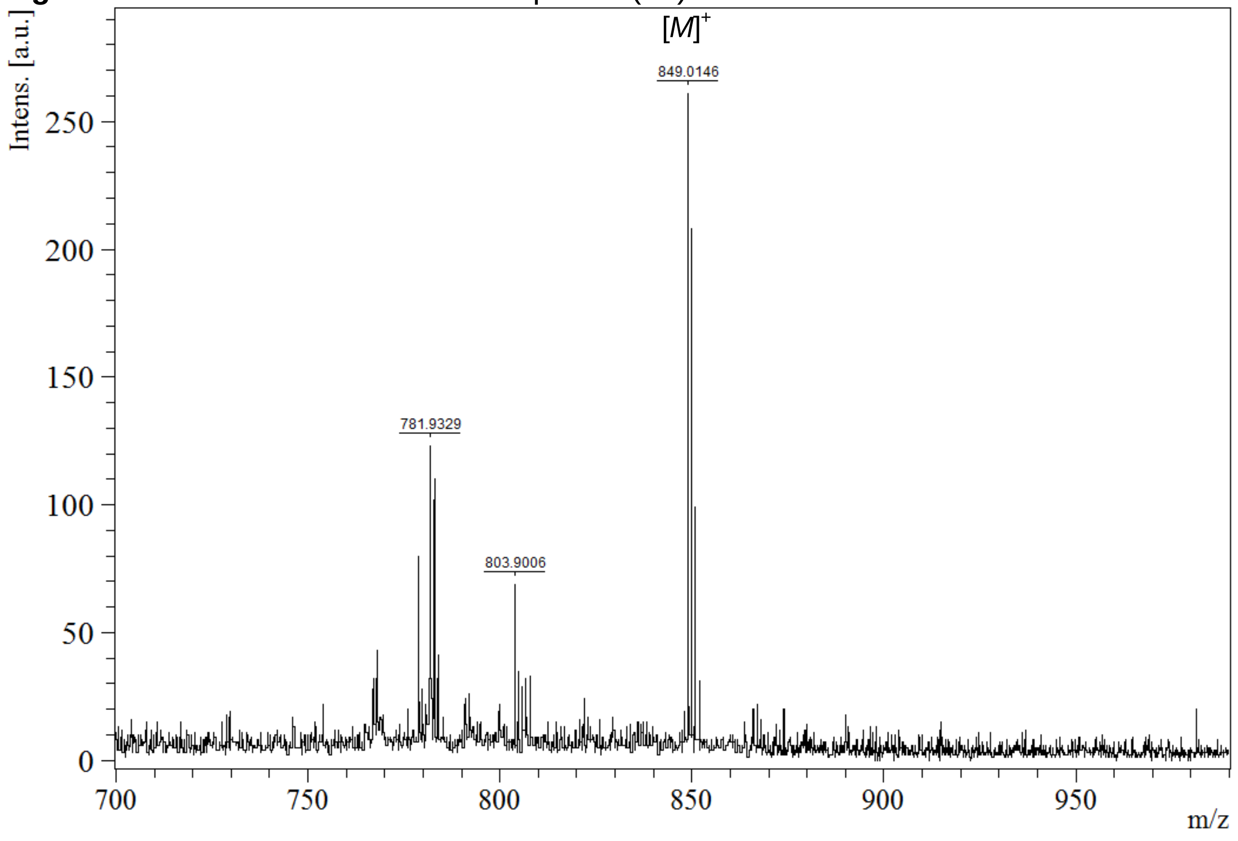

Figure S19. ^{1}H and ^{31}P NMR of compound (**18**).

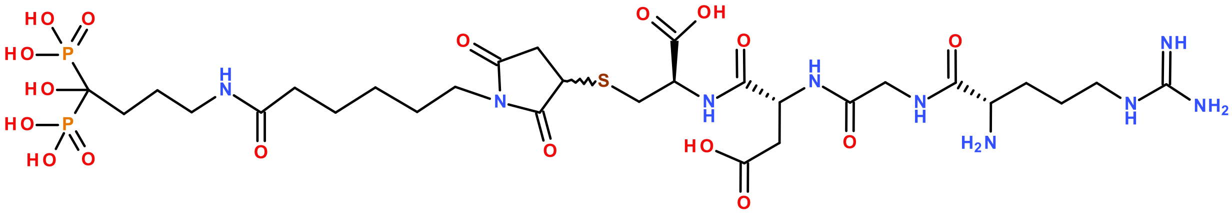

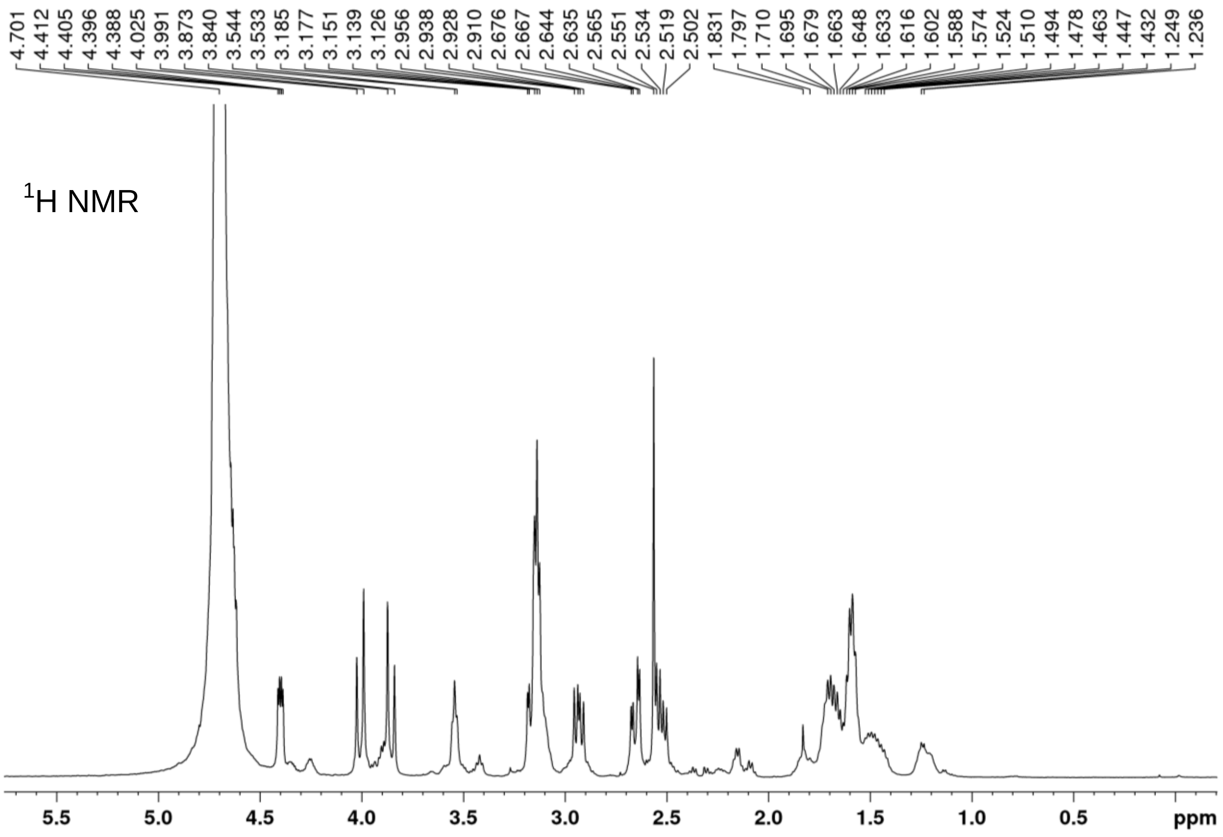

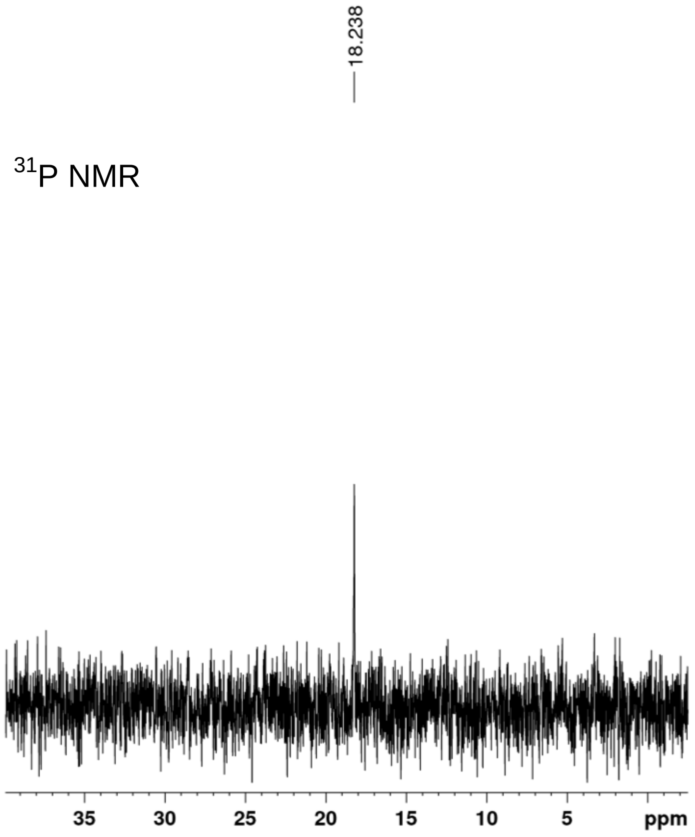

Figure S20. MALDI TOF/TOF of compound (**18**).

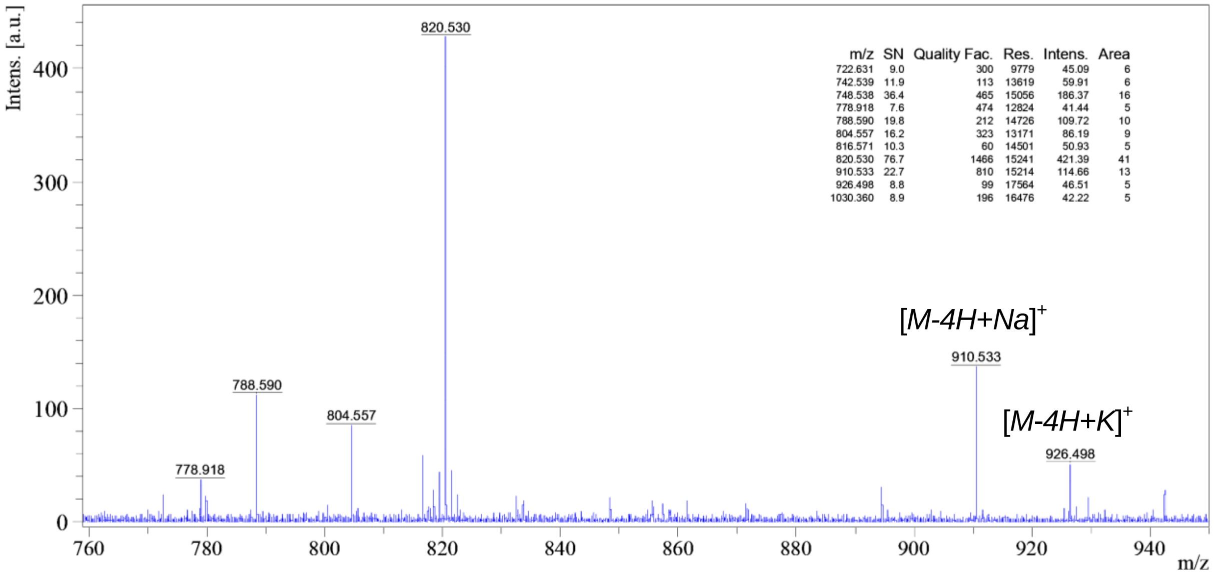

m/z	SN	Quality Fac.	Res.	Intens.	Area
722.631	9.0	300	9779	45.09	6
742.539	11.9	113	13619	59.91	6
748.538	36.4	465	15056	186.37	16
778.918	7.6	474	12824	41.44	5
788.590	19.8	212	14726	109.72	10
804.557	16.2	323	13171	86.19	9
816.571	10.3	60	14501	50.93	5
820.530	76.7	1466	15241	421.39	41
910.533	22.7	810	15214	114.66	13
926.498	8.8	99	17564	46.51	5
1030.360	8.9	196	16476	42.22	5

Figure S21. ^{1}H and ^{31}P NMR of compound (**19**).

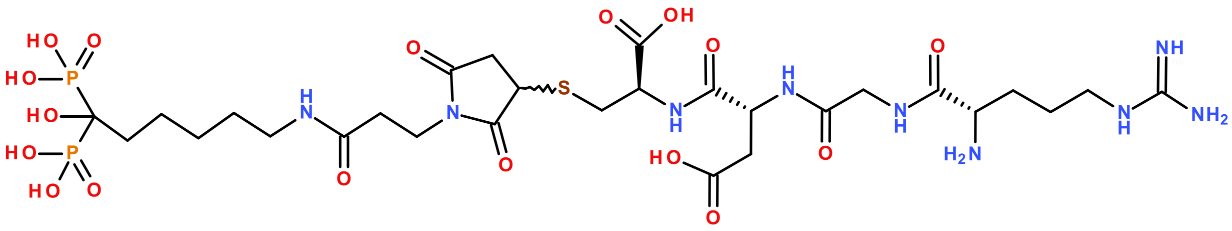

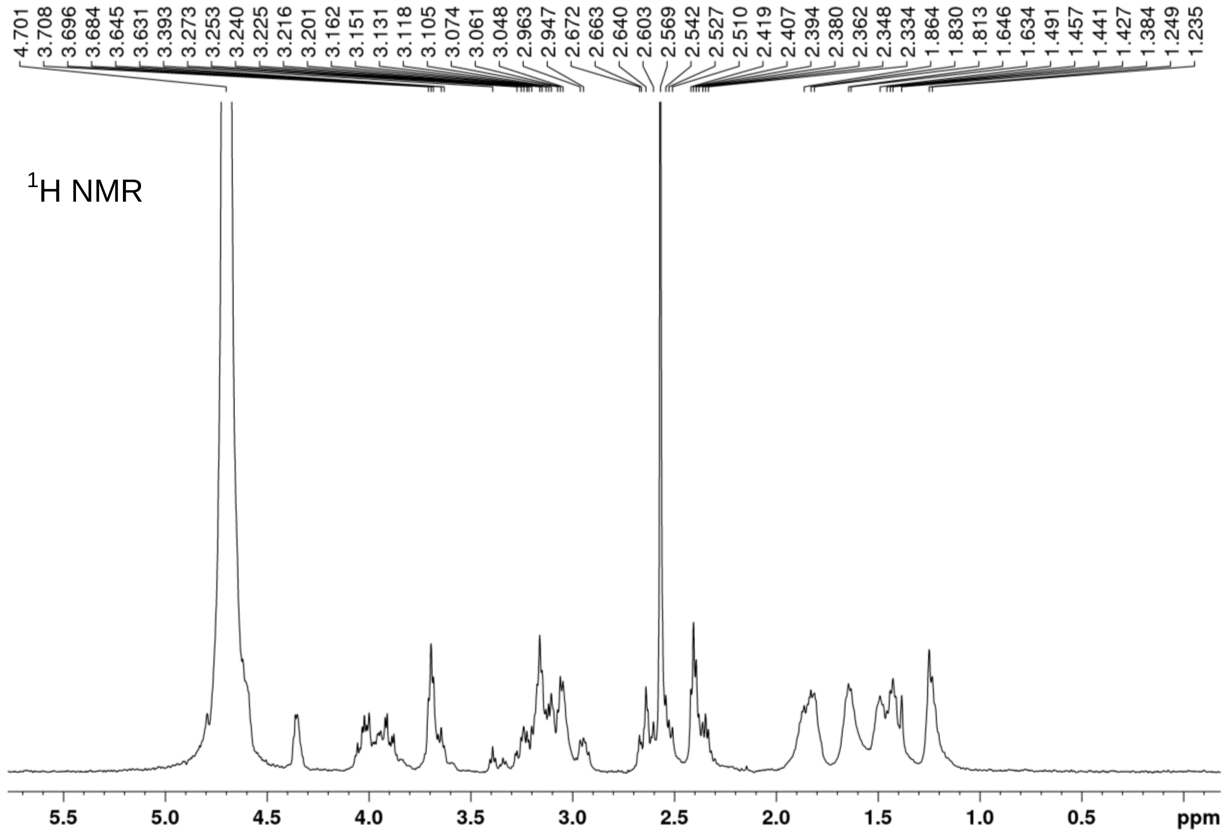

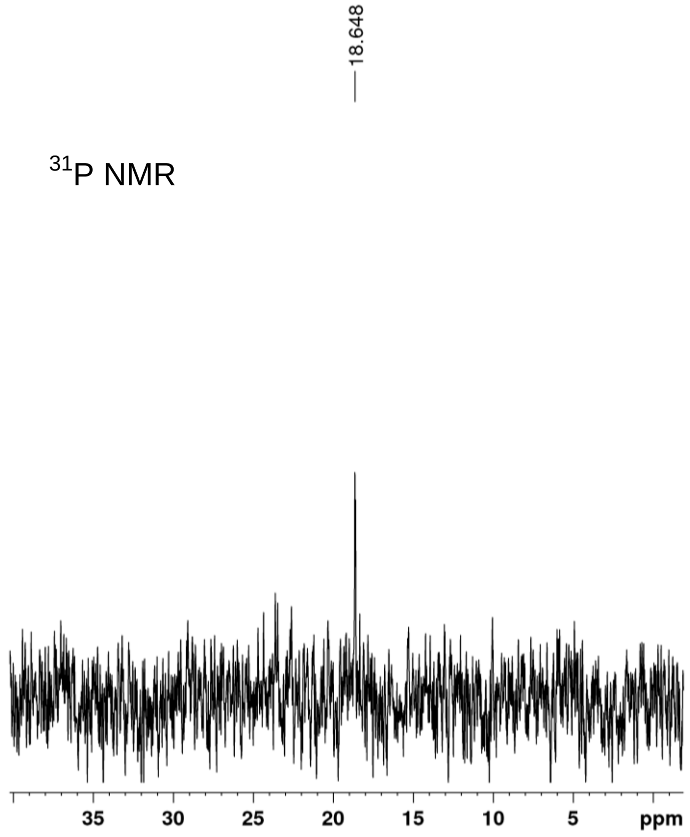

Figure S22. MALDI TOF/TOF of compound (**19**).

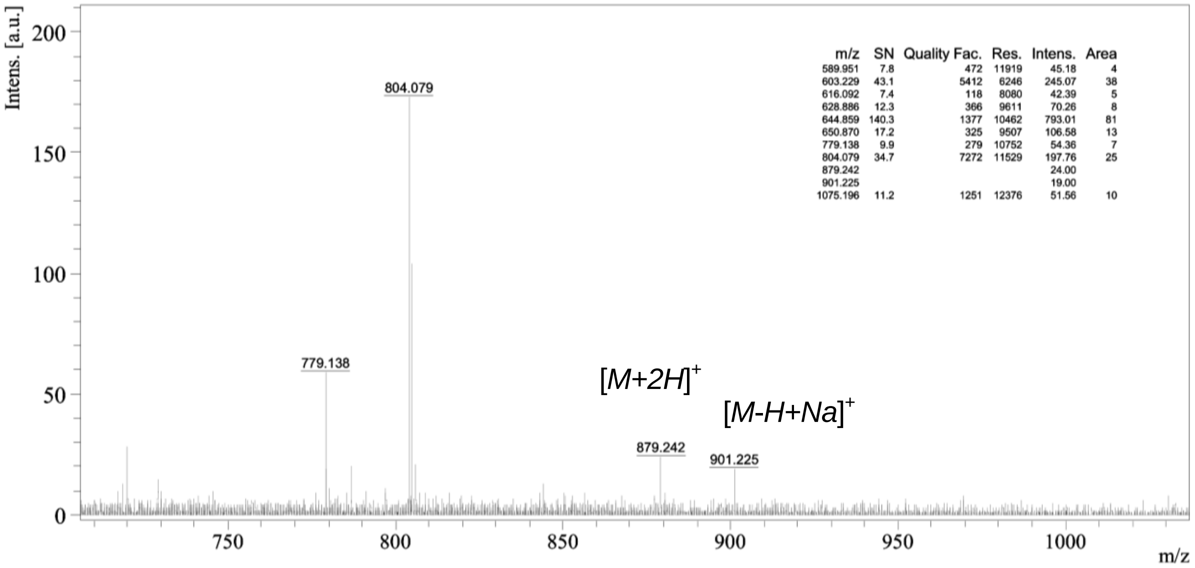

m/z	SN	Quality Fac.	Res.	Intens.	Area
589.951	7.8	472	11919	45.18	4
603.229	43.1	5412	6246	245.07	38
616.092	7.4	118	8080	42.39	5
628.886	12.3	366	9611	70.26	8
644.859	140.3	1377	10462	793.01	81
650.870	17.2	325	9507	106.58	13
779.138	9.9	279	10752	54.36	7
804.079	34.7	7272	11529	197.76	25
879.242				24.00	
901.225				19.00	
1075.196	11.2	1251	12376	51.56	10

Figure S23. ^{1}H and ^{31}P NMR of compound (**20**).

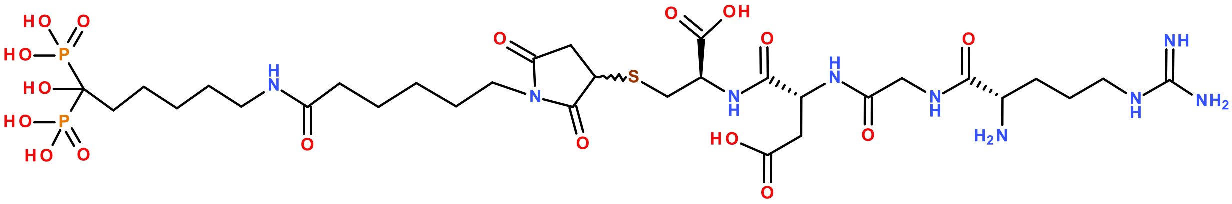

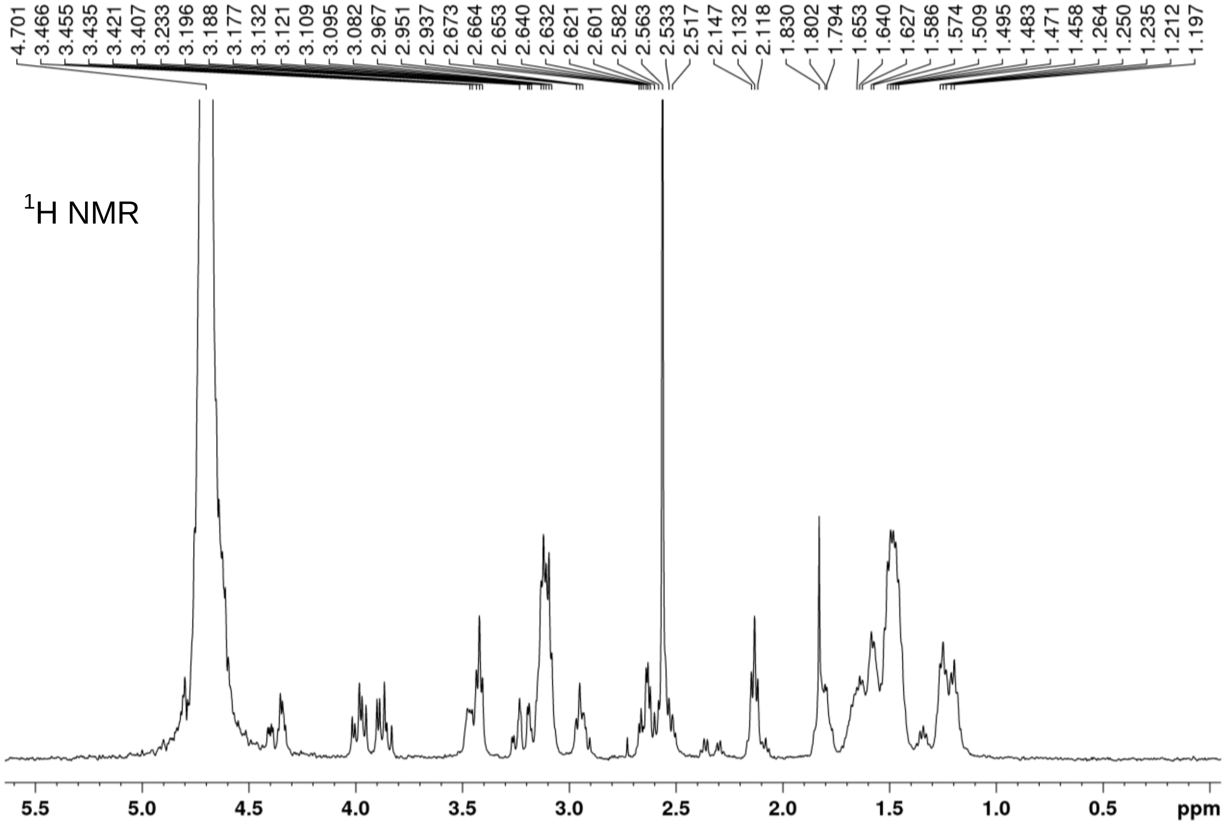

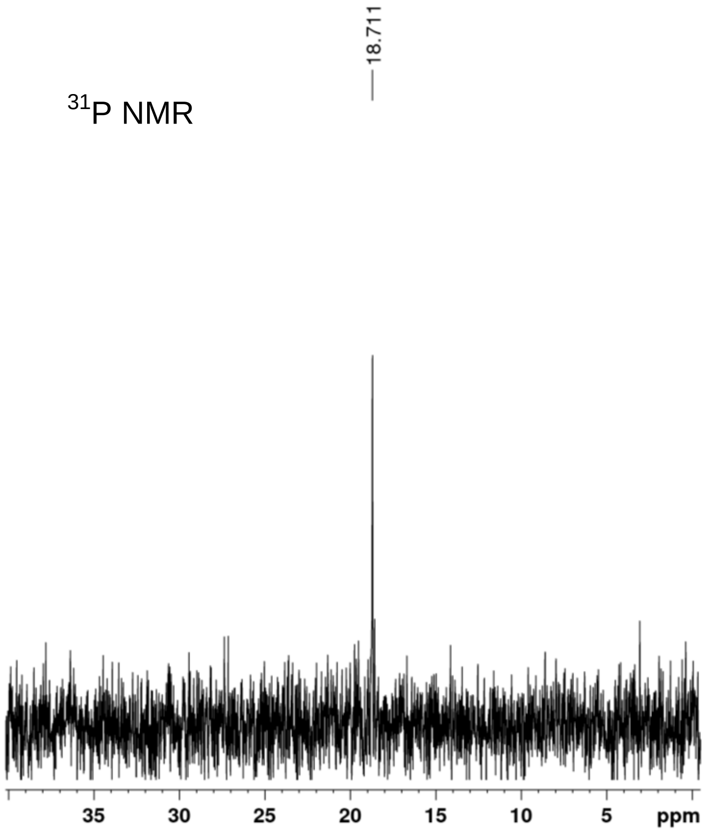

Figure S24. MALDI TOF/TOF of compound (**20**).

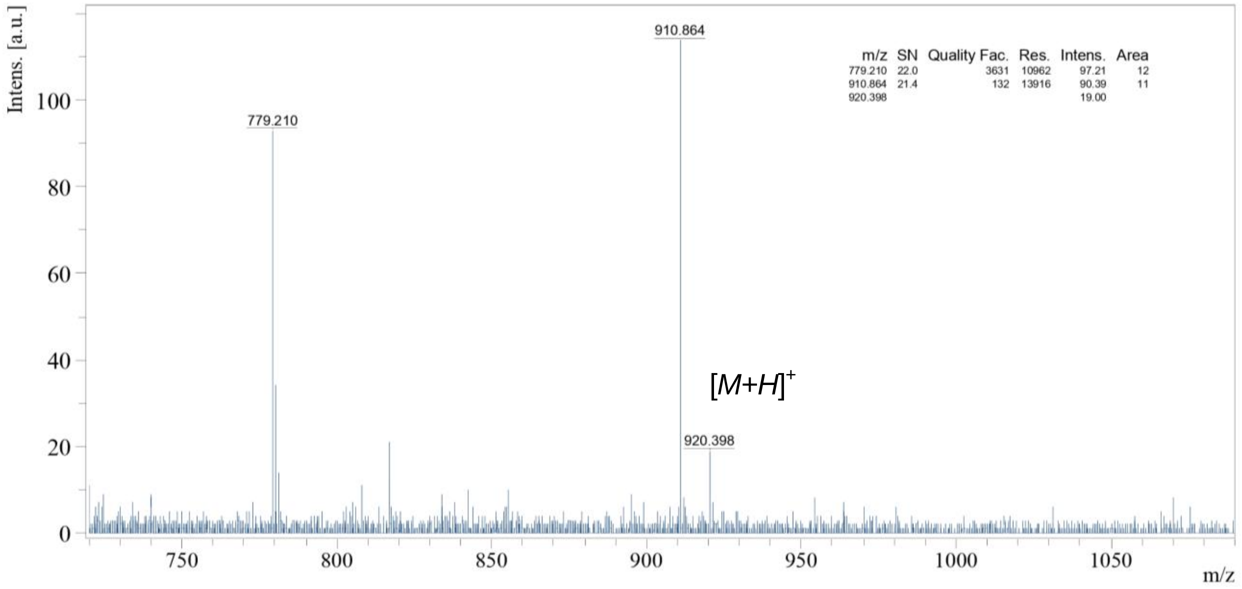

Figure S25. ^{1}H and ^{31}P NMR of compound (**21**).

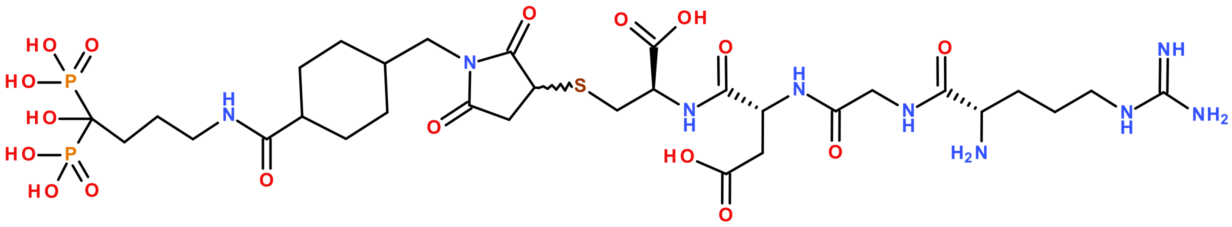

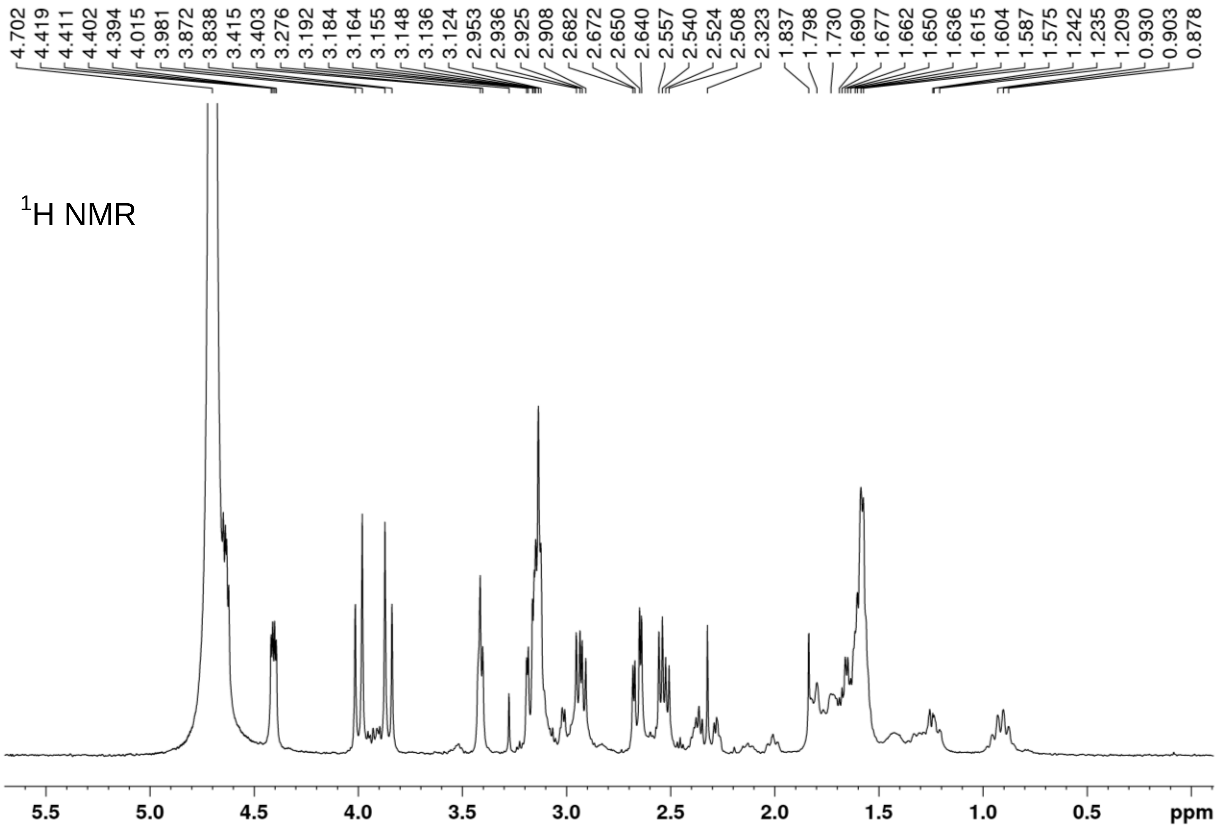

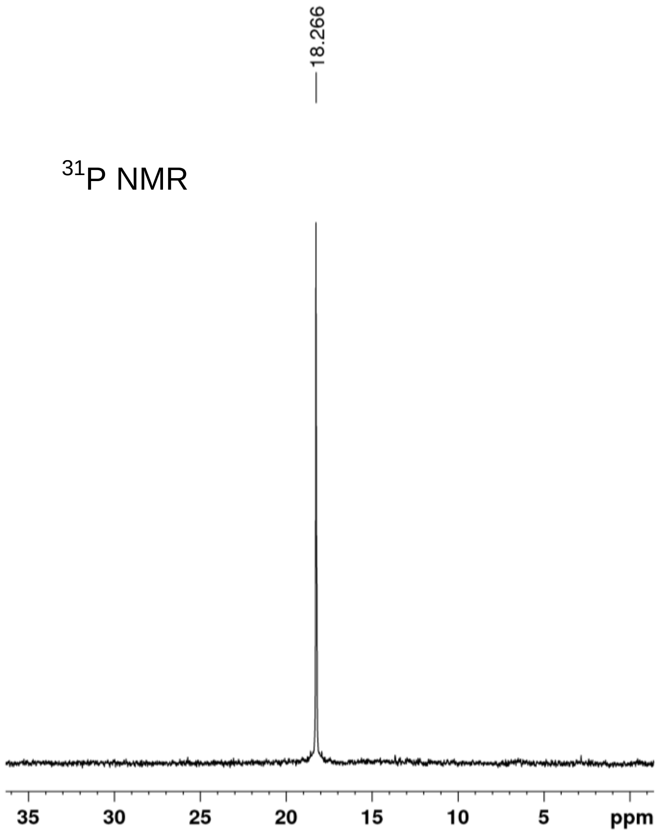

Figure S26. MALDI TOF/TOF of compound (**21**).

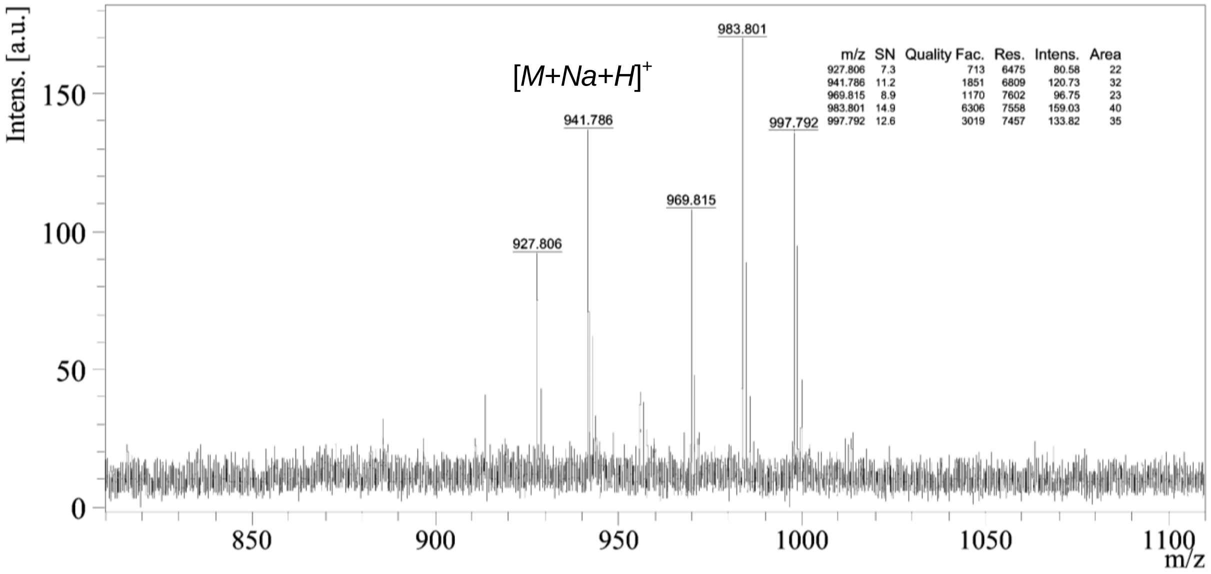

Figure S27. ^{1}H and ^{31}P NMR of compound (**22**).

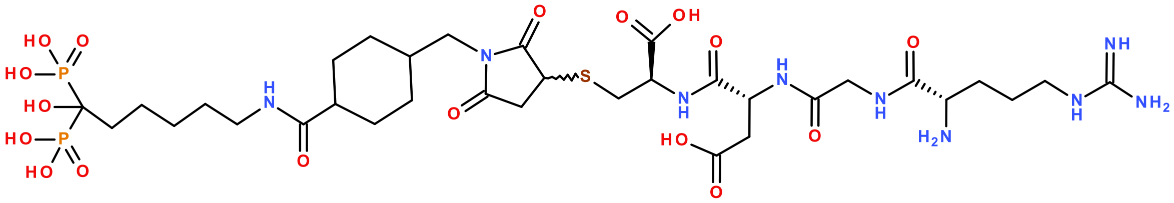

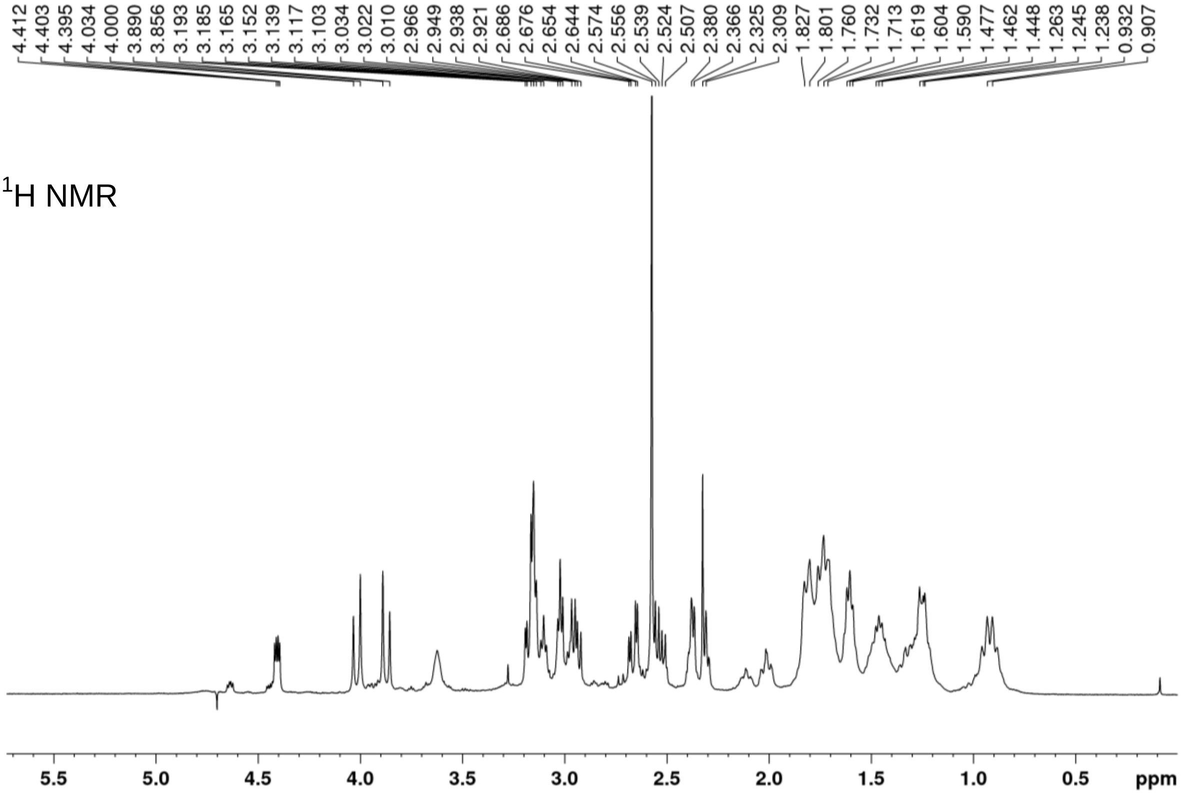

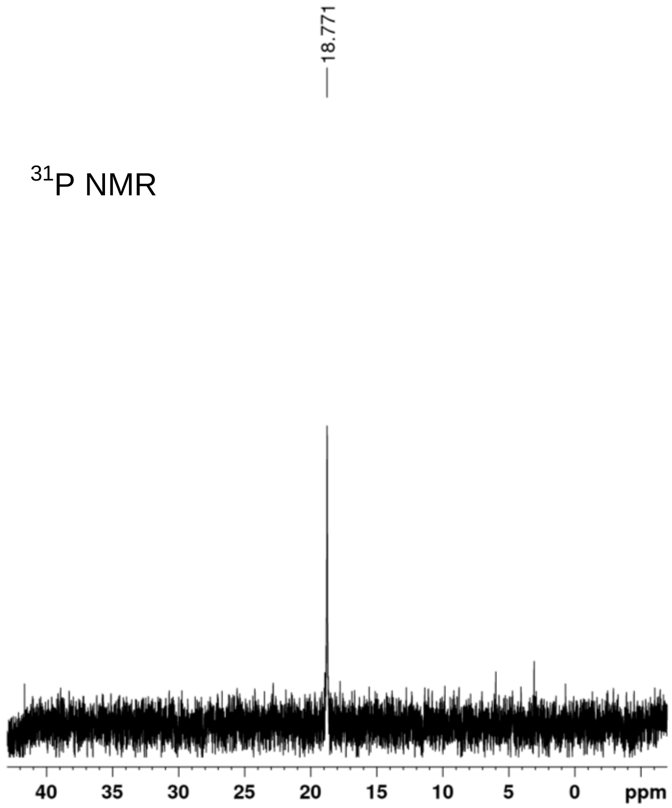
18.771
31P NMR
40
35
30
25
20
15
10
5
0
ppm

Figure S28. XPS spectra of Ti-PEO modified by compounds **15**-**20**.

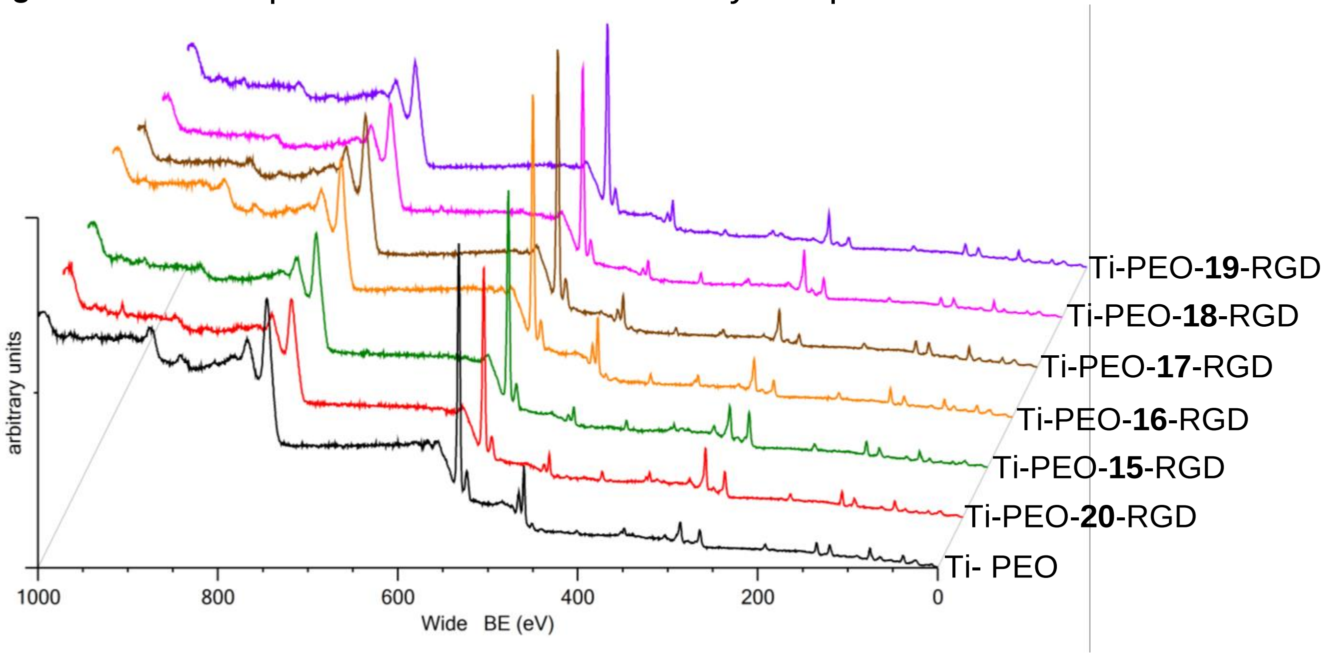

Supplement: Supplementary file 1 [file molecules-25-00229-s001.pdf]
